# Probing Glycan-Gold Nanoparticle Architectures: Glycan Type, Density, and Linker Length, Governing Multivalent Lectin Binding and Viral Inhibition

**DOI:** 10.1021/acsami.5c25677

**Published:** 2026-04-06

**Authors:** Maisie Holbrow-Wilshaw, Darshita Budhadev, Amy Madeleine Kempf, Inga Nehlmeier, Erin Tait, Stefan Pöhlmann, W. Bruce Turnbull, Yuan Guo, Dennis McGonagle, Dejian Zhou

**Affiliations:** † Leeds Institute of Rheumatic and Musculoskeletal Medicine, School of Medicine, 4468 University of Leeds, Leeds LS2 9JT, United Kingdom; ‡ School of Chemistry and Astbury Centre for Structural Molecular Biology, University of Leeds, Leeds LS2 9JT, United Kingdom; ∇ Infection Biology Unit, German Primate Center−Leibniz Institute for Primate Research, 37077 Göttingen, Germany; § Faculty of Biology and Psychology, University of Göttingen, 37073 Göttingen, Germany; ∥ School of Biomedical Sciences and Astbury Centre for Structural Molecular Biology, University of Leeds, Leeds LS2 9JT, United Kingdom; ⊥ School of Food Science and Nutrition and Astbury Centre for Structural Molecular Biology, University of Leeds, Leeds LS2 9JT, United Kingdom

**Keywords:** multivalent lectin−glycan interaction, DC-SIGN, glycoconjugate, gold nanoparticle, fluorescence quenching, thermodynamics, viral inhibition

## Abstract

Multivalent lectin-glycan interactions (MLGIs) are widespread and vital for pathogen infection, cell–cell communication, and immune regulation, making them attractive therapeutic targets. Despite significant efforts, research progress in MLGI targeting therapeutics remains limited, due to our incomplete understanding of the structural and biophysical mechanisms of some key MLGIs, which has hampered the design of spatially matched multivalent therapeutics. Moreover, the overlapping glycan specificities of various lectins make it difficult to target MLGIs with high potency and selectivity. To address this challenge, we have recently developed polyvalent glycan nanoparticles (glycan-NPs) as biophysical probes for MLGIs. The NPs’ unique, size-dependent optical properties are exploited as sensitive readouts for quantifying MLGI affinities and thermodynamics, while their nanoscale size and high electron microscopy contrast are exploited for probing binding modes and binding site orientation. Despite this success, how design features such as glycan type, density, and linker flexibility govern glycan-NP MLGI properties remains underexplored. In this work, we coated gold nanoparticles (GNPs) with varying densities of a lipoic acid-oligo­(ethylene glycol)-α-manno-α-1,2-biose (DiMan) or fucose (Fuc) ligand of varying linker lengths and studied their MLGIs with DC-SIGN, an important tetrameric lectin viral receptor found on dendritic cells. Using our recently established GNP fluorescence quenching assay, we reveal that displaying DiMan or Fuc polyvalently on a GNP surface greatly enhances their DC-SIGN affinity, with low nanomolar apparent *K*_d_s, ∼480 000-fold tighter than the corresponding monovalent binding. Their binding is driven by enthalpy, with favorable enthalpic but unfavorable entropic terms, and their absolute values depend on linker flexibility and glycan density. At high glycan densities, a short and less flexible linker is favored by maximizing enthalpic gains while minimizing entropic penalties, whereas at low glycan densities, a long and flexible linker is favored by increasing the reach and adaptivity of terminal glycans to maximize favorable enthalpic gains. These results reveal a delicate balance between glycan density and flexibility in controlling glycan-NP MLGI properties and their underlying thermodynamic mechanisms. Finally, we demonstrate that GNP-glycans potently block DC-SIGN-augmented viral entry into host cells with subnanomolar IC_50_s, which are positively linked to their DC-SIGN MLGI affinity.

## Graphical abstract

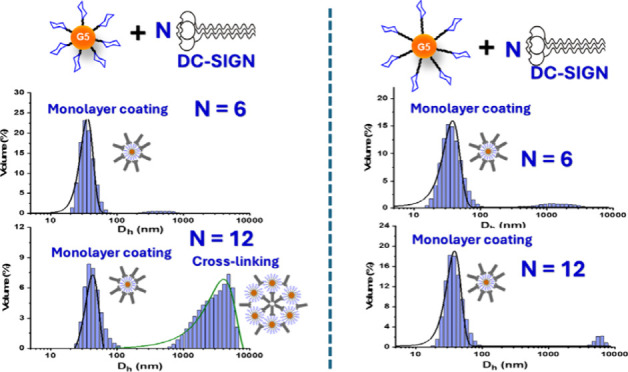

## Introduction

Lectin-glycan interactions (LGIs) are central to a wide range of biological processes, including pathogen recognition, immune regulation, and cell–cell communication.^1–4^ In nature, these interactions play critical roles in orchestrating immune responses, where lectins expressed on immune cells recognize specific glycan patterns on pathogens to trigger defense mechanisms, or conversely, where pathogens exploit host glycans or lectins to initiate infection.^5,6^ Due to the structural diversity of carbohydrates, LGIs are typically characterized by high specificity but relatively low affinity at the monovalent level, with equilibrium binding dissociation constant (*K*_d_) often in the millimolar range. Such level of binding is usually too weak to be considered biofunctional. Therefore, strong and bioactive interactions often rely on exploiting multivalency and spatial matches to enable the simultaneous engagement of multiple binding sites to drastically enhance affinities.^7^ The overall strength and specificity of multivalent LGIs (MLGIs) are thus determined not only by the monovalent binding affinity but also by the spatial arrangement, binding mode, valency, and flexibility of the interacting partners.^7^

In multivalent systems, the spatial match between the lectin’s carbohydrate recognition domains (CRDs) and the glycan presentation on the opposing surface determines whether binding occurs via simultaneous engagement of all binding sites or through less efficient cross-linking which leads to large scale assemblies. Spatially perfectly matched systems can maximize favorable enthalpic terms while minimize entropic penalties, leading to the formation of small, uniform lectin-ligand complexes with high affinities.^8–10^ In contrast, spatially mismatched systems often cross-link each other to maximize enthalpic terms and form extended networks or assemblies, but this typically yields large entropic penalties, giving rise to weaker overall affinities than the former.^7,11,12^ Understanding how molecular design parameters dictate these binding modes is therefore critical for rationally designing glycan-based therapeutics and probes to target specific lectin-mediated processes, such as viral attachment, immune signaling modulation, or cancer cell recognition.

Moreover, precise knowledge of the thermodynamic contributions to binding strength can further guide the design of agents to target specific MLGIs. Knowing whether the interactions are driven by entropic and/or enthalpic terms can direct choices based on scaffold size, shape, ligand density and flexibility to give the optimal agent for the desired applications. Despite their importance, information concerning the MLGI binding modes, and how these modes influence affinity and underlying binding thermodynamics, remains largely underexplored. This knowledge gap is primarily attributed to the limitations of current biophysical techniques in probing these inherently complex and flexible interactions. For instance, isothermal titration calorimetry (ITC) and surface plasmon resonance (SPR) are two of the most commonly employed techniques for studying the thermodynamics of binding interactions, including MLGIs.^13,14^ However, ITC faces challenges in determining the accurate affinities of cross-linking and/or very strong interactions, which can complicate the interpretation of ITC data.^13,15^ Similarly, SPR struggles to dissect the individual contributions of LGIs to the overall MLGI affinity and specificity, as these are heavily influenced by the density and orientation of the immobilized binding partner on the surface.^14^ Therefore, while these conventional biophysical techniques can provide some key kinetic and thermodynamic information, they cannot offer structural information such as binding modes, and binding site orientations, which are crucial for developing multivalent therapeutics against specific MLGIs.

Over the past two decades, a broad range of nanoscale scaffolds, such as polymers,^16,17^ dendrimers,^18–20^ liposomes,^21,22^ proteins,^23–25^ and inorganic nanoparticles (NPs),^26–28^ have been developed for displaying multivalent glycans to probe and manipulate MLGIs. Additional useful properties can also be offered by the inorganic NP cores, such as fluorescence, superparamagnetism, or photothermal properties.^28–31^ This affords these NPs extra capabilities which can be harnessed to provide further therapeutic benefit, or readout signals for probing binding behaviors. This multifunctionality is present in semiconductor quantum dots (QDs) and gold nanoparticles (GNPs), which have strong fluorescence (with QDs)^32^ or strong fluorescence quenching (with GNPs)^33,34^ properties, allowing them to partake in fluorescence resonance energy transfer (FRET, with QD) or nano surface energy transfer (NSET, with GNP) as readout signals for binding quantification.^27,35–38^ Among these scaffolds, inorganic NPs, especially GNPs, have emerged as particularly versatile scaffolds owing to their well-defined size and synthesis methods, ease of functionalization via robust gold–thiol chemistry, excellent biocompatibility, and unique optical properties. The high electron density and strong fluorescence quenching ability of GNPs make them powerful tools for both structural and quantitative studies of biomolecular interactions by combining electron microscopy imaging and fluorescence analysis.^37,38^

Recent studies have demonstrated that polyvalent glycan coated GNPs and QDs can amplify their MLGI affinities by >10^6^ fold over the corresponding monovalent bindings.^36,38,39^ Using tetrameric lectins such as DC-SIGN^40^ and its closely related homologue DC-SIGNR^41^ as model systems, our group has employed glycan-functionalized GNPs/QDs to reveal key structural determinants of glycan-NPs’ MLGI specificity and viral recognition.^27,37–39,42^ Despite sharing high sequence identity (∼80%), identical CRD-mannose monovalent binding motifs,^43^ and similar tetrameric architectures^44,45^ DC-SIGN and DC-SIGNR exhibit distinct multivalent glycan binding properties and biological functions. For example, DC-SIGN is more effective than DC-SIGNR in enhancing the cellular entry of viruses such as Ebola and HIV, whereas only DC-SIGNR, but not DC-SIGN, can promote West Nile Virus infection.^46–48^ Using glycan-NPs as new biophysical probes, we have previously revealed that DC-SIGN and DC-SIGNR bind glycan-NPs differentially, where DC-SIGN typically engages all four of its CRDs simultaneously in binding to a single glycan-NP,^36–38^ while DC-SIGNR tends to cross-link with multiple glycan-NPs, leading to the formation of large supramolecular assemblies with weaker overall MLGI affinities.^36–38^ These different MLGI binding behaviors may contribute to the different biological activities of these lectins.^46–48^

While our previous studies have highlighted the importance of nanoparticle size^38,39^ and shape^42^ in controlling glycan-NPs binding with multimeric lectins such as DC-SIGN, several critical molecular-level parameters remain poorly understood. First, our previous glycan-NP probes were all based on mannose/α-manno-α-1,2-biose (DiMan)-, but not fucose (Fuc)-, containing glycans. While both are natural glycan ligands for DC-SIGN, they exhibit different binding profiles with DC-SIGN CRD. Fuc exhibits relatively simple binding, by coordinating to a Ca^2+^ ion in the CRD primary binding via its 3, 4-OH groups.^49^ Whereas DiMan exhibits extended binding interactions, besides coordinating to the Ca^2+^ ion in the CRD primary binding site via 3, 4-OH groups and van der Waals interactions with Val351 via its first mannose unit, it also forms hydrogen bonding with Ser360 and Glu358 and van der Waals interactions with Phe313 via the second mannose unit.^11^ Hence how different glycan types with different CRD binding motifs control glycan-NP MLGI properties remain to be explored. Second, the length and flexibility of the linker connecting the glycan to the nanoparticle surface and the surface glycan density are expected to have profound effects on the spatial presentation and dynamic accessibility of the glycans. The linker acts as a molecular “spacer,” defining the effective reach and orientational freedom of the terminal glycan relative to the solid, nondeformable nanoparticle core. A short and relatively rigid linker may restrict glycan accessibility, hindering the optimal alignment with multiple CRDs, whereas a long and flexible linker may facilitate multivalent binding by affording glycans with extra accessibility and adaptivity, but it may also introduce extra entropic penalties, due to increased conformational restriction upon binding. Similarly, surface glycan density determines the average interglycan distance and steric crowding on the scaffold surface. Extremely high glycan densities can create a rigid glycan surface unable to adapt to the binding surface of target lectins, especially on cell surfaces where spatial movements of membrane bound lectins are constrained in ways that do not exist in solution, whereas an overly sparse glycan surface may limit simultaneous engagement of multiple CRDs from one lectin with one glycan-NP, both cases can lead to suboptimal multivalent binding. Despite their evident importance, systematic investigations of how glycan type, surface density and linker flexibility govern glycan-NP MLGI affinity, thermodynamics, and binding mode are still scarce.

To address these knowledge gaps, we have systematically designed and synthesized a series of lipoic acid-oligo­(ethylene glycol) (LA-EG_n_-) based ligands appending a terminal DiMan or Fuc as the target glycan because of their different binding profiles with DC-SIGN CRD. Using these glycan ligands, we have prepared a series of glycosylated GNPs (glycan-GNPs) with varying flexible linker lengths and surface glycan densities to elucidate how these parameters impact their multivalent binding with DC-SIGN. By employing dynamic light scattering (DLS) and GNP fluorescence quenching assays, we quantify both the binding strength and structural consequences of linker length and density variations on DC-SIGN MLGI properties with glycan-GNPs. Additionally, temperature-dependent binding affinity measurements in combination with van’t Hoff analysis allow us to extract binding enthalpy and entropy changes, providing new insights into how glycan flexibility and density modulate the thermodynamic driving forces of such MLGIs. Finally, we evaluate the potential of such glycan-GNPs as entry inhibitors to block augmentation of Ebola virus glycoprotein (EBOV-GP) driven viral entry by DC-SIGN at the cell surface, revealing that our glycan-GNPs have high antiviral potencies (sub-nM *IC*_*50*_ values), which are positively linked to their DC-SIGN binding affinities measured by the solution-based GNP fluorescence quenching assay.

## Results and Discussion

### Ligand Design and Synthesis

The GNP-glycans were designed with considerations such as core material, carbohydrate type, linker length, ligand density and scaffold size in mind. These design features are known to play critical roles in modulating the avidity and selectivity of MLGIs. Here, gold nanoparticles with an average core diameter of ∼ 5 nm (denoted as G5 hereafter, see SI, Figure S1B) were coated in multifunctional ligands containing three functional domains: a lipoic acid (LA) group for strong binding to the GNP surface via the formation of 2 strong Au–S bonds to impose high stability, a terminal α-manno-α-1,2-biose (DiMan) or α-L-fucose (Fuc) for specific binding to DC-SIGN, and to connect them, a flexible oligo­(ethylene glycol) linker of varying length to enhance water solubility, resist nonspecific interactions and explore terminal glycan flexibility.^50–52^ We chose DiMan and Fuc as the two target glycans here because of their different binding profiles with DC-SIGN CRD,^11,49^ allowing for probing their impact on glycan-NP MLGI properties. Three oligo­(ethylene glycol) linkers with EG repeat units (EG_n_) of n = 2, 6, and 12 were chosen. The reasons for choosing such flexible, hydrophilic EG_n_ linkers are 3-fold, first they enable observation of the role of ligand flexibility on binding strength; second, they form a design feature to protrude the terminal glycan head groups away from the GNP surface, minimizing any steric clashes that may hinder lectin binding; and third, they can enhance water solubility and colloidal stability of the GNPs and reduce nonspecific interactions,^50–52^ ensuring that all binding interactions observed are due to specific MLGIs only.

The schematic structures of the glycan-GNP conjugates and the chemical structures of the glycan ligands employed in this study are shown in Figure 1. The synthetic routes to the LA-EG_n_-glycan multifunctional ligands are shown in Scheme 1–2.

**Figure 1:**
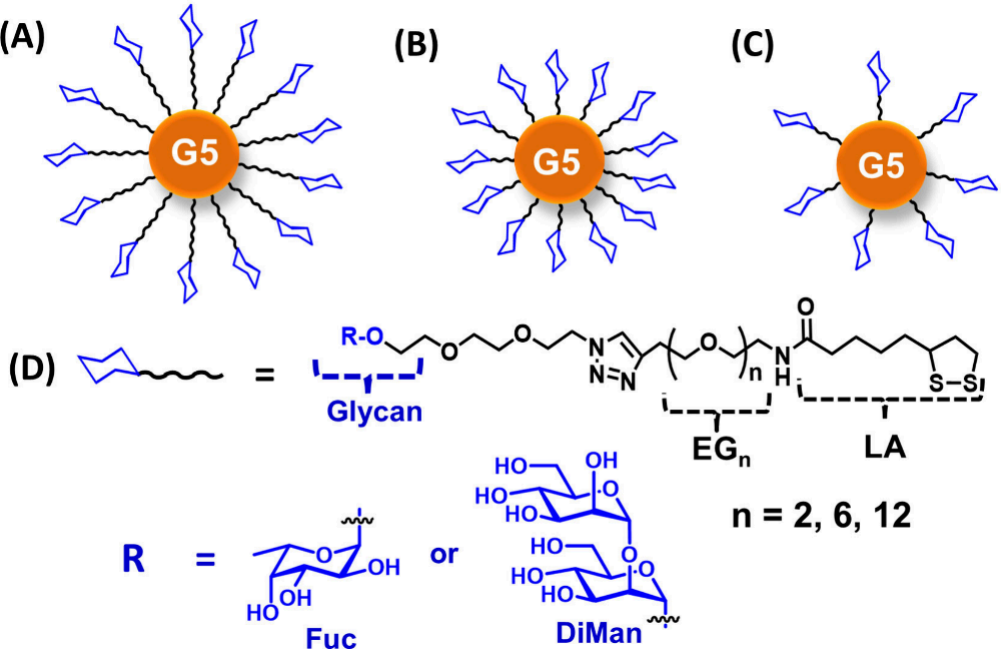
(A–C) Schematic structures of the GNP-glycan conjugates used in this study. The GNP with an average diameter of ∼5 nm (G5) is coated with LA-EG_n_-glycan ligands containing either a terminal DiMan or Fuc. The valency of the LA-EG_n_-glycan ligand (with three EG_n_ linker lengths of n = 2, 6, and 12) on each G5 is varied to probe their impacts on G5-glycan MLGI properties with DC-SIGN. (D) Chemical structures of the glycan ligands employed in this study.

**Scheme 1:**
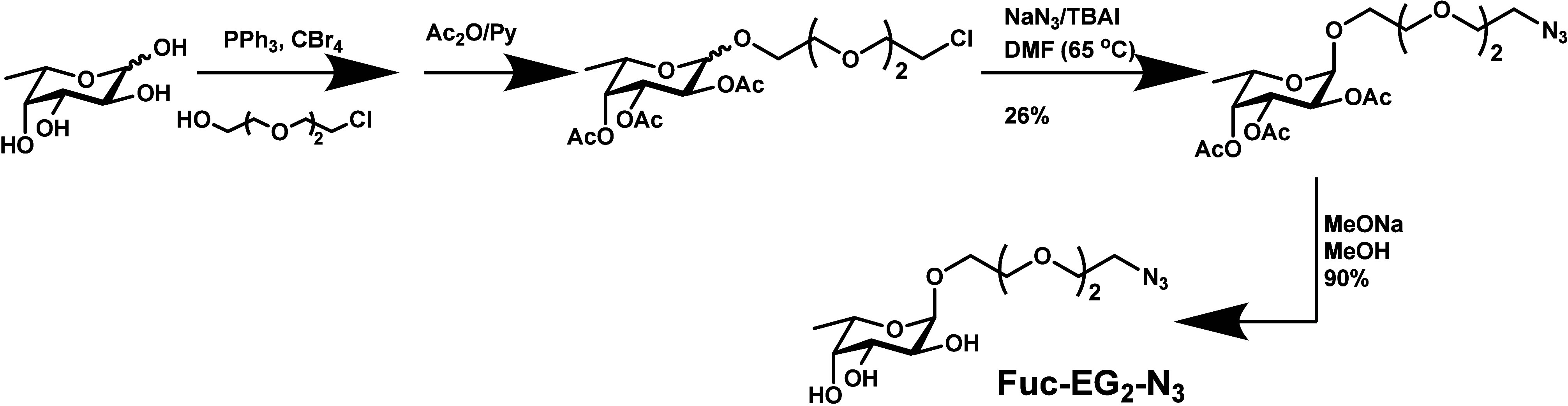
Synthetic Route to Azido-EG_2_-Modified Fucose (N_3_-EG_2_-Fuc)

**Scheme 2:**
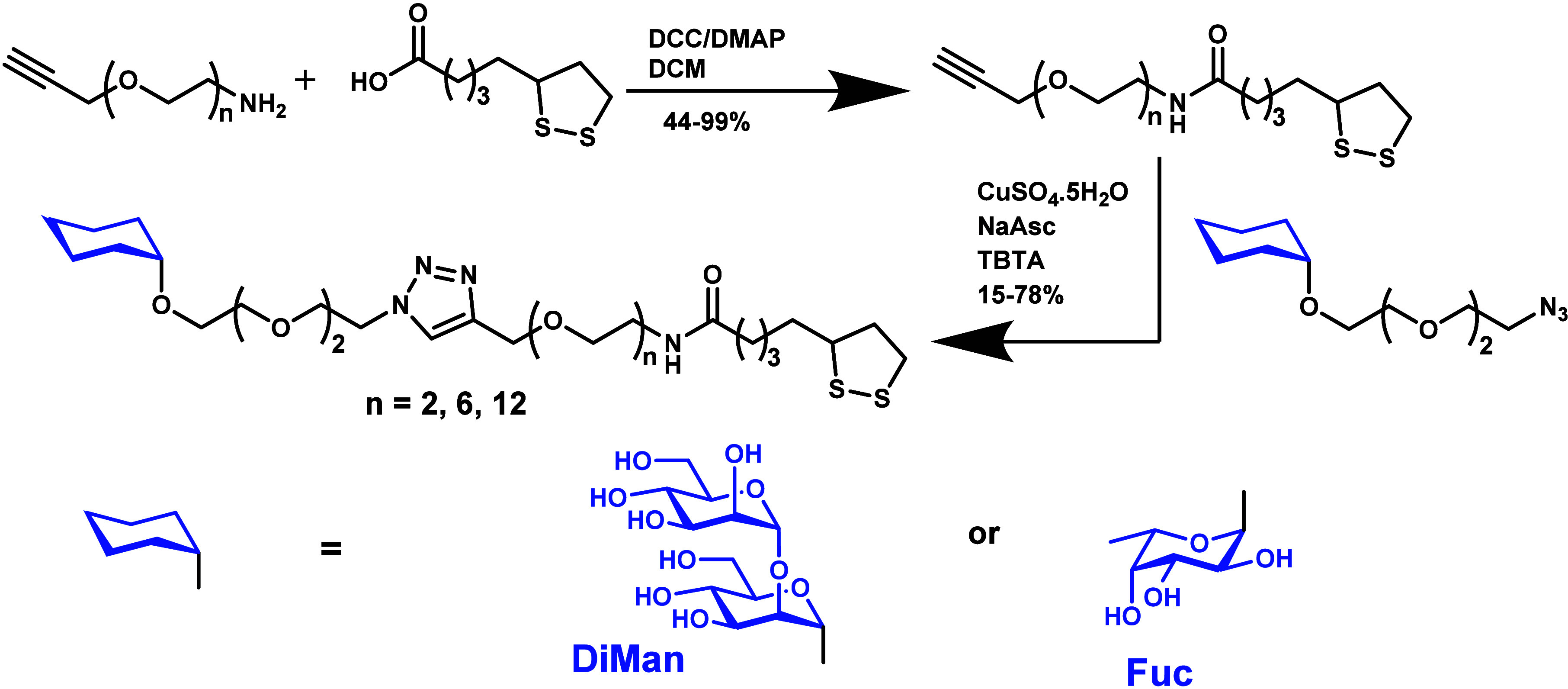
Synthetic Route to LA-EG_n_-DiMan and LA-EG_n_-Fuc Ligands (where n = 2, 6, or 12)

Briefly the azido-EG_2_ modified fucoside was prepared in a protecting group free Fisher glycosylation with L-fucose and the acceptor, monochlorinated triethylene glycol, in the presence of the Appel reagent (CBr_4_, PPh_3_). Acetylation followed by azidation afforded the protected α-anomer and deprotection gave the desired azido fucoside ligand (N_3_-EG_2_-Fuc). Detailed synthesis procedures and their spectroscopy characterization data are given in the Experimental section and also in the Supporting Information (SI). The azido-EG_2_-modified DiMan (N_3_-EG_2_-DiMan) was prepared via our previously reported procedures.^36,37^

LA-EG_n_ based linker molecules were prepared using a standard dicyclohexylcarbodiimide (DCC)/ N’N’-dimethylaminopyridine (DMAP) mediated amide coupling between commercial HC≡C-EG_n_-NH_2_ and lipoic acid (LA) to give LA-EG_n_-C≡CH linkers. They were then coupled efficiently to the N_3_-EG_2_-Fuc or N_3_-EG_2_-DiMan via Cu catalyzed click chemistry to give the desired LA-EG_n_-Fuc/DiMan as reported previously.^37,39^

### GNP-glycan Preparation

Gold nanoparticles (GNPs) were prepared via our previously established protocols by heating gold­(III) chloride trihydrate in water with trisodium citrate in the presence of potassium carbonate and tannic acid to ∼ 75 °C, forming citrate stabilized GNPs of roughly 5 nm in diameter (denoted as G5-citrate, SI, Figure S1).^39^ By exploiting the strong Au–S interactions, the G5-citrates were coated with the desired ligands in a simple one-pot self-assembly procedure by stirring air stable LA-EG_n_-ligands with the G5-citrate directly in water for 48 h. The disulfide bonds in the LA based ligands are cleaved upon binding to gold surfaces, and displace the weakly bound citrate ions, forming self-assembled monolayers on the G5 surfaces that are identical to their reduced dihydrolipoic acid counterparts.^37^ Three batches of each G5-glycan conjugates were prepared under three different LA-EG_n_-glycan ligand: G5 molar ratios (LGMRs) of 1000, 500, and 300. We have shown previously that 1000 LGMR with LA-EG_n_-glycan ligands can create a densely packed, saturated glycan coating on G5 surface.^37^ The lower LGMRs of 500 and 300 were used to reduce glycan valencies to investigate their impact on glycan-GNP MLGI properties. Any free unbound ligands were removed by ultrafiltration using 10K MWCO filter tubes and washing with pure water. Using the ligand amount difference between that used and that remained unbound in the supernatant and washup water after conjugation (measured by a phenol-sulfuric acid based carbohydrate quantifying assay),^36,37^ the numbers of glycan ligands bound on each G5 were estimated as ∼ 600, ∼ 360, ∼ 170 for G5-glycans prepared under the LGMR of 1000, 500, and 300 (SI, Table S2), translating into LA-EG_n_-glycan ligand conjugation efficiencies of ∼ 60%, ∼ 72% and ∼ 57%, respectively. Consistent with this result, the negative zeta potential of G5-ctriate (−25.5 ± 1.0 mV) was reduced progressively after treatment with increasing LGMRs of both LA-EG_2_-DiMan/Fuc ligands (SI, Figure S2), indicating that the negatively charged citrate ions on G5-citrate surface were increasingly displaced by neutral LA-EG_2_-glycan ligands. The reduction of negative zeta potential was especially pronounced for G5-glycans prepared at 1000 LGMR, at ∼ −7.5 mV for both ligands, indicating that almost all citrate ions were completely displaced. This result is fully consistent with the increasing glycan valencies for G5-glycans made at higher LGMRs described above.

The G5-glycans were characterized by UV–visible absorption spectroscopy. Compared to the G5-citrate, a small but discernible red shift (∼4–5 nm) in the characteristic SPR absorption peak of G5 was observed in all G5-glycans (SI, Figure S3). This result is fully consistent with literature for GNPs after coating with thiolated ligands, due to a change of GNP local refractive index after coating of the LA-glycan ligands.^37,39^ All G5-glycan conjugates were found to be highly stable, showing no visible color changes (an indication of aggregation)^37^ or precipitation after extended storage in a fridge at 4 °C for several months. All G5-glycans were found to form uniform and monodisperse particles in a binding buffer (20 mM HEPES, 100 mM NaCl, 10 mM CaCl_2_, pH 7.8, SI, Figures S4–S6) measured by dynamic light scattering (DLS). Their hydrodynamic diameters (*D*_h_s) were found to be in the range of 10.4 to 17.8 nm, depending on the glycan valency (LGMRs used in preparation), type and EG_n_ linker length (SI, Table S1). For G5-glycans prepared at the highest LGMR of 1000, those with the longest EG_12_- linkers tended to display largest *D*_h_s among all G5-glycans with the same terminal glycan, matching well to what expected for G5 surface being densely coated with LA-glycan ligands, allowing their terminal glycans being extended further away from G5 surface by the longest linkers. However, this trend does not apply for those prepared at the lowest LGMR of 300, where the *D*_h_ for G5-EG_6_-glycans and G5-EG_12_-glycans are almost identical. This result indicates that under such conditions, the density of LA-EG_n_-glycan ligands on the G5 surface is sparse enough to allow the EG_n_ linkers to fold up, adopting a collapsed conformation with compact *D*_h_s which can impact the availability of the terminal glycans for lectin binding. Using the *D*_h_ values, glycan valency (SI, Table S2) and the method reported by the Mirkin group,^53^ the average interglycan distances for all G5-glycans were calculated to be in the range of 0.85 – 1.9 nm, depending on the linker length, glycan valency and type. A general trend here is that increasing the EG_n_ linker length or decreasing glycan valency results in greater interglycan distances as expected (SI, Table S3). Interestingly, for G5-glycans prepared at 1000 and 500 LGMRs, their average interglycan distances (e.g., 0.85–1.46 nm) are comparable to the major interglycan sequon spaces (e.g., 0.7–1.3 nm) found on the HIV surface heavily glycosylated gp160 trimer,^54^ the glycoprotein that is responsible to DC-SIGN facilitated HIV infection. Therefore, such G5-glycans may act as good mimics for probing gp160-DC-SIGN interactions which facilitate the HIV infection.

#### Probing DC-SIGN-G5-EG_n_-glycan Binding Mode by Dynamic Light Scattering (DLS)

Previously, the binding mode between DC-SIGN and G5-EG_2_-DiMan was studied by monitoring the hydrodynamic diameter (*D*_h_) of the G5-DiMan-DC-SIGN complex as varying ratios of DC-SIGN molecules were added to a fixed concentration of G5-EG_2_-DiMan. This revealed that multiple copies of DC-SIGN can bind to a single G5-EG_2_-DiMan particle, forming small lectin-G5 assemblies of approximately 40 nm in *D*_h_ when saturated.^37^ This size is consistent with a single G5-EG_2_-DiMan particle coated with a monolayer of DC-SIGN molecules, suggesting that all four CRDs in each DC-SIGN molecule engage with a single G5-EG_2_-DiMan. To further extend our knowledge on the binding between G5-glycans and DC-SIGN, the binding modes of G5-glycans made in LGMRs of 1000, 1:500 and 1:300 were studied to determine if the EG_n_ linker length and/or glycan valency can impact DC-SIGN binding mode and if a lower saturation point may be reached for G5-glycans with the lower valencies. The concentration of G5-glycans was fixed at 20 nM and the concentration of DC-SIGN was increased from 20–320 nM (i.e., protein to G5 ratio (PGR) of 1 – 16). DC-SIGN alone displayed a single narrow Gaussian distribution with a *D*_h_ of ∼ 14.0 ± 2.0 nm (mean ± 1/2 fwhm; fwhm = full width at half-maximum of Gaussian fit, see SI, Figure S8B). Representative *D*_h_ histograms for G5-EG_2_-DiMan (LGMR = 1000) binding with varying PGRs of DC-SIGN are shown in Figures 2A1–4. The *D*_h_ histograms with Gaussian fits for all G5-glycan (LGMR = 1000)-DC-SIGN complexes under a variety of PGRs are given in SI, Figures S10–15. The corresponding *D*_h_-PGR relationships for DC-SIGN binding with G5-EG_n_-DiMan and G5-EG_n_-Fuc are shown in Figures 2B and 2C, respectively.

**Figure 2:**
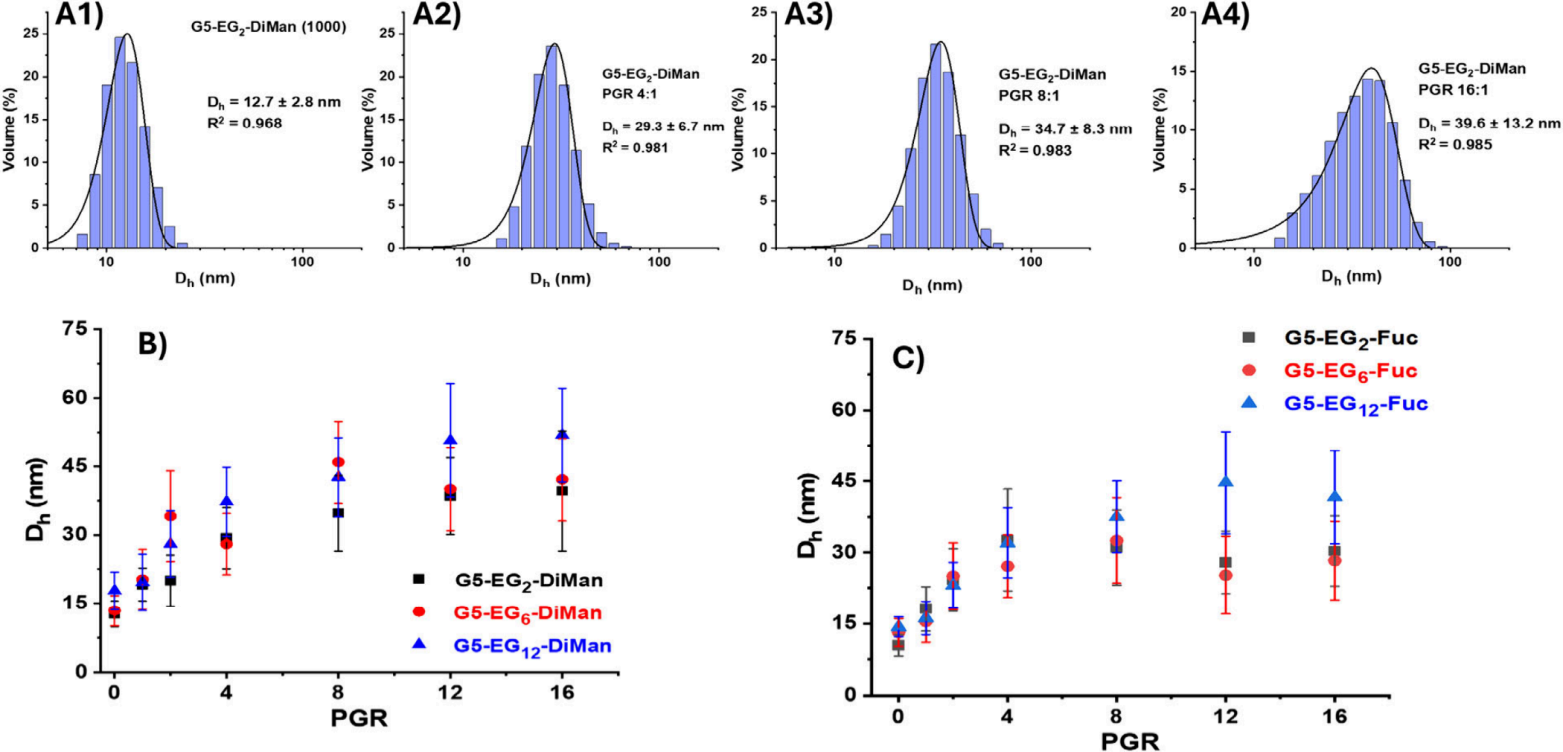
Representative *D*_h_ (volume population) histograms and Gaussian fits for G5-EG_2_-DiMan (LGMR = 1000) binding with DC-SIGN at varying protein:G5 ratios (PGRs): (A1) PGR = 0, (A2) PGR = 4, (A3) PGR = 8, and (A4) PGR = 16. Comparison of *D*_h_ (mean ± 1/2 fwhm)–PGR relationships for DC-SIGN binding with (B) G5-EG_n_-DiMan (n = 2, 6, and 12; all made at LGMR = 1000) and (C) G5-EG_n_-Fuc (n = 2, 6, and 12; all made at LGMR = 1000).

In general, the *D*_h_ of the G5-EG_n_-glycan (LGMR = 1000) + DC-SIGN mixtures increased gradually with the increasing PGR, reaching a plateau at a PGR of around 8–12, after which the *D*_h_ values remained roughly constant. This behavior is similar to that observed previously for DC-SIGN binding with G5-EG_2_-DiMan. The saturated *D*_h_s for G5-glycans with the longest EG_12_-linkers were generally larger than those with the shorter EG_2_ linkers (maximal *D*_h_: 52 ± 10 nm vs. 40 ± 13 for n = 12 and 2, respectively). In the absence of DC-SIGN, the *D*_h_s of G5-EG_12_-glycans are ∼ 5 nm larger than their G5-EG_2_-glycan counterparts, but the *D*_h_ differences of their DC-SIGN complexes at saturation are ∼ 12 nm, suggesting more DC-SIGN molecules are bound to each G5-EG_12_-glycans than to G5-EG_2_-glycans. This result is consistent with the larger total glycan surface areas (i.e., larger *D*_h_s, see SI, Table S1) of G5-EG_12_-glycans over G5-EG_2_-glycans, allowing the former to accommodate more DC-SIGN molecules before surface saturation and hence *D*_h_ plateauing at higher PGRs.

Although the *D*_h_s of the complexes appear to plateau at PGRs of 8–12, this does not necessarily reflect the true saturation of the G5-EG_n_-glycans with DC-SIGN. Using a surface binding footprint of ∼ 35 nm^2^ per DC-SIGN tetramer,^36^ and the surface areas of G5-EG_n_-glycans calculated from their *D*_h_s (∼510, ∼ 540, ∼ 995 nm^2^, for EG_2_-, EG_6_-, and EG_12_- DiMan respectively, and ∼ 410, ∼ 540, and ∼ 640 nm^2^ for EG_2_-, EG_6_-, and EG_12_- Fuc, respectively) then PGRs of ∼ 12–30 with DC-SIGN were estimated to be able to fully saturate the surface of G5-EG_n_-glycans, depending on linker lengths. For G5-EG_12_-glycans prepared at LGMR of 1000, saturation binding with 30 DC-SIGN molecules on each G5-EG_12_-glycan should be possible. This would require 120 surface glycans which is considerably fewer than the 550+ surface glycans found on each G5 surface as described in the earlier section and SI, Table S2. Consequently, the *D*_h_ plateauing at 12 DC-SIGN molecules per G5-EG_12_-glycan may be a result of an even distribution of these DC-SIGN molecules on the G5 surface, forming a roughly spherical G5-DC-SIGN core–satellite like complex with relatively large gaps between bound DC-SIGN molecules. Further binding of DC-SIGN molecules only occupies the gaps between bound DC-SIGN molecules without increasing the overall complex size. The *D*_h_ plateauing at a lower PGR of 8 for the G5-EG_2_-glycans may be a combination of the same effect and steric hindrance, as crowding at near-saturation prevents further DC-SIGN molecules from binding.

Repeating the DC-SIGN binding studies with G5-EG_n_-glycans made in LGMRs of 500 and 300 largely followed the same trend, with the *D*_h_ of the species corresponding to single G5-glycan particles coated with a monolayer of DC-SIGN molecules plateauing at PGRs of around 6:1. The *D*_h_s of the DC-SIGN monolayer coated G5-glycan species at each PGR along with their Gaussian fits are shown in SI, Figures S16–29. Less variations in the saturated *D*_h_ values were observed for the 500 and 300 LGMR G5-glycans than the 1000 LGMR ones with the three different EG_n_ linker lengths, consolidating the assumption that the G5-glycans made at lower LGMRs are less sterically hindered than those at 1000. Therefore, they have sufficient space to fold up upon the linkers, giving a smaller *D*_h_ even after binding with DC-SIGN. Representative *D*_h_ histograms for G5-EG_n_-DiMans (n = 2, 6, and 12, LGMR = 300) binding with DC-SIGN at three different PGRs of 0, 6, and 12 are shown in Figure 3.

**Figure 3:**
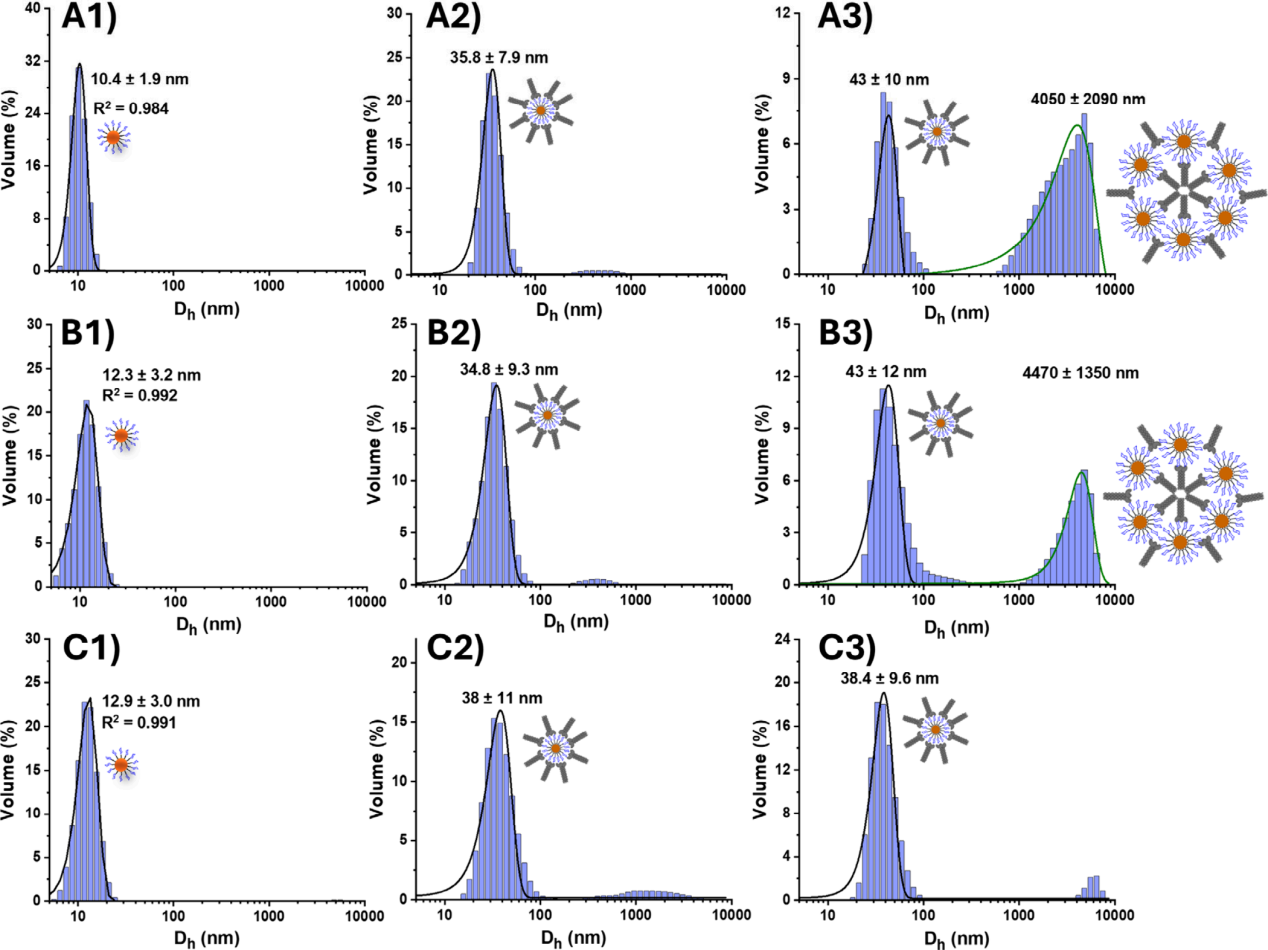
Hydrodynamic diameter (*D*_h_, volume population) distribution histograms and Gaussian fits for G5-EG_2_-DiMan (LGMR 300) (A1) before and after mixing with DC-SIGN at PGRs of (A2) 6 and (A3) 12, G5-EG_6_-DiMan (LGMR 300) (B1) before and after mixing with DC-SIGN at PGRs of (B2) 6 and (B3) 12, and G5-EG_12_-DiMan (LGMR 300) (C1) before and after mixing with DC-SIGN at PGRs of (C2) 6 and (C3) 12. The *D*_h_ peaks corresponding to single G5-glycan particles (A1–C1), single G5-glycan particles coated with a monolayer of DC-SIGN molecules (A2–C2), and large scale G5-glycan-DC-SIGN cross-linked assemblies (A3–B3) are depicted next to the corresponding peaks.

All samples showed the presence of *D*_h_ values corresponding to the DC-SIGN monolayer coated single G5-glycan particles, especially at the low PGRs of ≤ 8, which are often the only or the dominant species. Interestingly, for the G5-EG_2/6_-DiMan prepared at LGMR of 300, some much larger structures with *D*_h_s of ∼ 4000 nm were also observed at a high PGR of 12, indicating that a second cross-linking mode of binding occurred. Such large cross-linked species were almost absent at the lower PGR of 6. This result is likely due to a spatial mismatch between the binding surface of the DC-SIGN tetramers and the G5-glycans under such low surface glycan valencies and large interglycan distances, rendering it less likely that glycans on one G5 surface can bridge all four CRDs for every bound DC-SIGN tetramer, especially at the high PGR of 12. If one or more DC-SIGN CRDs remain unbound by one G5-glycan, a second G5-glycan may bind to such unoccupied CRDs to maximize the favorable binding enthalpy term, creating a large, cross-linking network. The relative abundance of the cross-linked to monolayer DC-SIGN coated G5-glycan species is reduced as the linker length increases from EG_2_ to EG_12_ (Figure 3, **A3-C3**, especially for G5-EG_12_-DiMan where the cross-linked species is almost diminished), indicating that the increased flexibility of the longer linkers affords G5-EG_12_-glycans better ability to bridge all four CRDs in one DC-SIGN tetramer, making it more likely to form small monolayer DC-SIGN coated single G5-glycan particles. This result highlights the importance of linker length and flexibility on G5-glycans in controlling the spatial constraints of the DC-SIGN CRDs and hence its binding mode.

#### Quantifying G5-EG_n_-glycan-DC-SIGN Binding Affinity

To investigate how glycan type, density and linker length affect their MLGI with DC-SIGN, we quantified their binding affinities using GNP’s strong fluorescence quenching properties.^37,55,56^ We have demonstrated previously that our GNP fluorescence quenching assay is not only sensitive but also robust in quantifying DC-SIGN based MLGI biophysical data: it gave effectively the same ΔH° values as those by isothermal titration calorimetry (ITC).^39^ G5 has a relatively low molar absorption extinction coefficient (1.1 × 10^7^ M^–1^ cm^–1^), it can offer a relatively wide concentration range (e.g., 1–100 nM) for affinity quantification without introducing significant inner-filter effects.^37,38^ Here, varying concentrations (3–100 nM) of Atto643 labeled DC-SIGN were mixed with 1 mol equiv of G5-glycans (prepared under three different LGMRs of 1000, 500 and 300) in a HEPES binding buffer (20 mM HEPES, 100 mM NaCl, 10 mM CaCl_2_, pH 7.8) containing large excess of a nontarget serum protein, bovine serum albumin (BSA, 1 mg/mL).^37^ BSA serves to minimize any possible nonspecific interactions and reduce nonspecific adsorption of proteins and/or GNPs on surfaces, which can be a major source of experimental errors for binding assays performed at low concentrations (≤10 nM).^57^ Moreover, serum proteins are of high abundance *in vivo*, therefore, this also makes the binding environments resemble real biological situations more closely. The G5-glycans + labeled DC-SIGN samples were incubated in the binding buffer for 20 min at room temperature before their fluorescence spectra (650 to 800 nm) were recorded under a fixed excitation wavelength, λ_ex_, of 630 nm. Atto643, a strongly hydrophilic, far-red emitting fluorophore, is employed here to minimize any interference to fluorescence measurement by GNP’s inner filter effect (GNP has low absorption in the far-red region of the visible spectra).^38^ Binding of labeled DC-SIGN to G5-glycans will bring the labeled Atto-643 fluorophores on DC-SIGN to the proximity to the G5 surface, leading to efficient quenching of Atto-643 fluorescence via GNP based NSET quenching mechanism, whereas unbound free DC-SIGN molecules are separated far away from G5 surface, leading to no fluorescence quenching (Figure 4A). Labeled DC-SIGN only samples (without G5-glycans) were also recorded under identical conditions, which serve as controls to determine the quenching efficiency (QE) at each concentration via eq 1, where IF_0_ and IF are the integrated fluorescence of labeled DC-SIGN in the absence and presence of 1 mol equiv of G5-glycans, respectively.^37^

$$\mathrm{QE (}\%)=\frac{IF_{0}-IF}{IF_{0}}\times 100$$ 1

**Figure 4:**
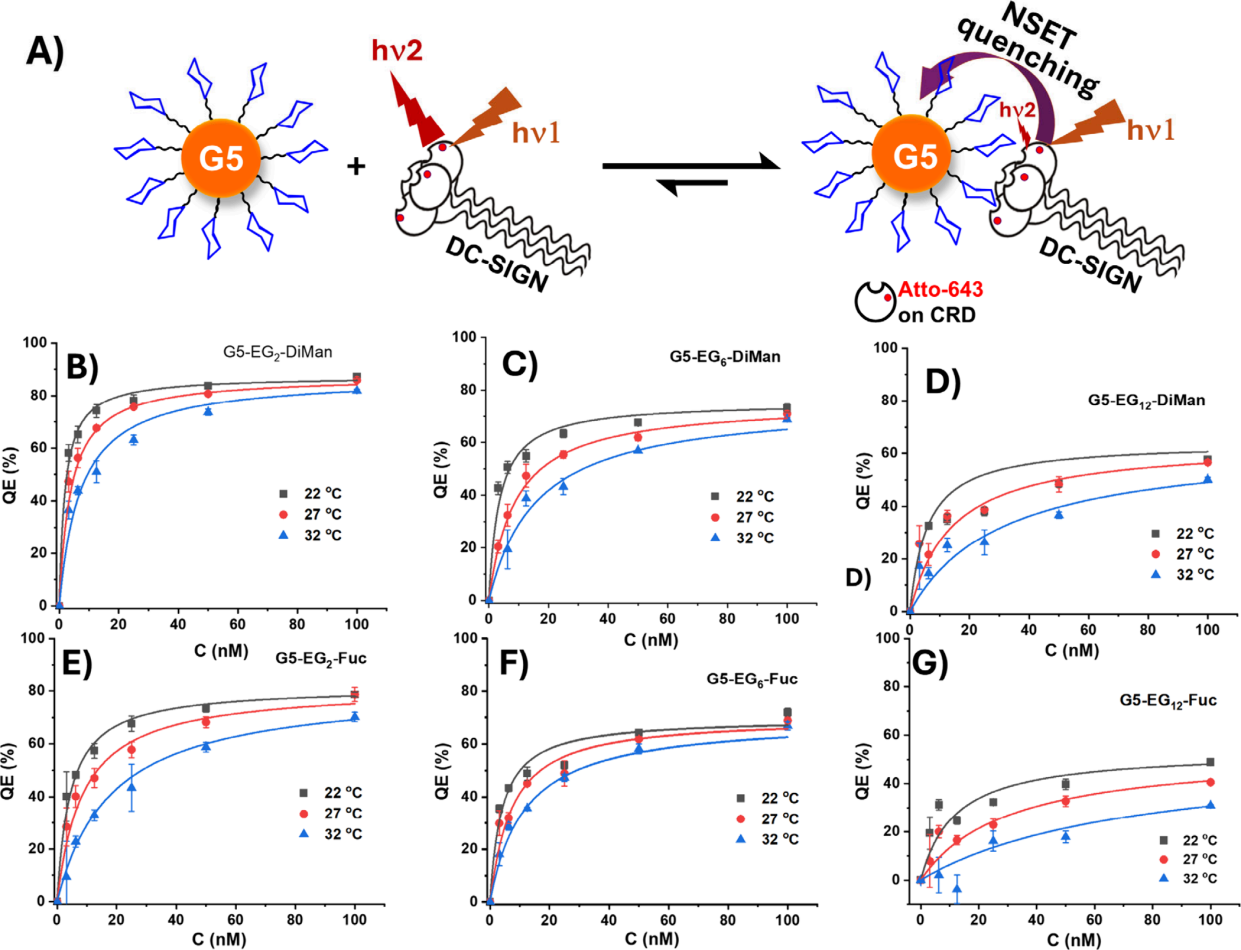
(A) Schematic principle of the GNP fluorescence quenching assay for quantifying DC-SIGN-G5-glycan binding. Before binding, the Atto-643 labels on free DC-SIGN are separated far from the G5 surface, giving off strong fluorescence upon excitation. After binding, the Atto-643 labels on DC-SIGN are brought into the proximity of the G5 surface, leading to efficient quenching of Atto-643 fluorescence by G5 in the proximity via the NSET mechanism. Therefore, the quenching efficiency (QE) is positively linked to the portion of DC-SIGN molecules bound to G5-glycans, making it a reliable readout for binding quantification. (B–F) QE–C relationships for DC-SIGN binding to various G5-glycans prepared at LGMR of 1000: (B) G5-EG_2_-DiMan, (C) G5-EG_6_-DiMan, (D) G5-EG_12_-DiMan, (E) G5-EG_2_-Fuc, (F) G5-EG_6_-Fuc, and (G) G5-EG_12_-Fuc at three different temperatures (22, 27, and 32 °C) fitted by Hill’s equation (eq 2). The fitting parameters are given in Table 1.

From this, *K*_d_ was calculated by fitting with Hill’s Equation (eq 2; where QE_max_ is the maximum quenching efficiency, [P] is the protein concentration, *K*_d_ is the apparent equilibrium binding dissociation constant (or effective concentration for 50% of binding/maximal quenching) and *n* is the Hill coefficient. Here, *n* = 1 was assumed for all fittings as no cooperativity was expected to occur since the affinity assays were performed under a PGR of 1. Where each G5-glycan should be bound with just one lectin, hence no intermolecular lectin–lectin interactions were expected to inhibit or promote further lectin-G5-glycan binding.^37–39^

2 $$\mathrm{QE}=\frac{QE_{\max}{\left[P\right]}^{n}}{{K_{d}}^{n}+{\left[P\right]}^{n}}$$

Representative fluorescence spectra of labeled DC-SIGN before and after mixing with 1 mol equiv of G5-EG_2_-DiMan (LGMR 1000) under varying concentrations (*C*s) are shown in SI, Figure S30. It reveals that in the absence of G5-EG_2_-glycan, DC-SIGN fluorescence increases linearly with *C*, whereas in the presence of 1 mol equiv of G5-EG_2_-DiMan, its fluorescence is greatly quenched and deviates increasingly more from the linear relationship with the increasing *C*, suggesting an increasingly higher portion of the added DC-SIGN molecules are bound to G5-EG_2_-DiMan and get quenched. The QE - *C* relationships and their best fits with eq 2 for DC-SIGN binding to G5-EG_n_-DiMan and G5-EG_n_-Fuc (LGMR = 1000, n = 2, 6, and 12) under three different temperatures, 22°, 27° and 32°, are given in Figures 4B-4D and 4E-4G, respectively.

It is apparent that the maximal quenching (QE_max_) is reduced with the increasing EG_n_ linker length and temperature, signifying weakened binding affinity. The resulting apparent binding *K*_d_s and QE_max_ values derived from fitting the QE-*C* plots are summarized in Table 1. The QE - *C* relationships and fittings for DC-SIGN binding to other G5-glycans (LGMRs = 500 and 300), and their fitting parameters are shown in SI, Figures S31–33 and Tables S4–S6, respectively. Based on the apparent *K*_d_ and QE_max_ values shown in Table 1 above, five conclusions can be drawn:

(1) The binding interactions between DC-SIGN and all G5-glycans are strong, with low nM apparent *K*_d_s (e.g., 1.9–14 nM), which translates into up to ∼ 480,000-fold tighter binding than the corresponding monovalent binding between DiMan and DC-SIGN CRD (*K*_d_ = 0.9 mM) measured by ITC.^58^ This result suggests that a polyvalent display of the glycan ligands on a G5 scaffold surface greatly enhances their MLGI affinity for DC-SIGN.
(2) QE_max_ generally decreases with the increasing EG_n_ linker length. Although GNPs can quench fluorescence almost completely when they are in close proximity,^55^ the QE decays rapidly with the increasing distance (inversely proportional to fourth power of distance).^33,55^ Since the G5 coated with glycan ligands of longer linkers have larger hydrodynamic radii, *R*_h_ (*R*_h_ = 1/2 *D*_h_, SI, Table S1) than shorter linkers, the bound DC-SIGN molecules (hence fluorophores) are placed further away from the GNP surface, leading to reduced QE_max_ as expected. For example, the QE_max_ obtained with G5-EG_12_-DiMan (LGMR 1000, *R*_h_ ∼ 8.9 nm) was ∼ 64%, which is considerably lower than ∼ 88% obtained with G5-EG_2_-DiMan (LGMR 1000, *R*_h_ ∼ 6.4 nm). The same trend was also observed for the G5-EG_n_-Fuc prepared at the highest LGMR of 1000. Although some deviations from this trend were observed for G5-glycans prepared at the lower LGMRs, especially at the lowest LGMR of 300. This is likely due to that, under such sparse ligand coating conditions, the long EG_n_-linkers can fold up, affording the G5-EG_n_-glycans with almost the same *R*_h_ values, despite variations in the EG_n_ linker length. As a result, the fluorophores on DC-SIGN molecules are placed at almost a constant distance from the G5 surface after binding, leading to similar QEs.
(3) DC-SIGN binds more strongly to G5-DiMans than to their G5-Fuc counterparts. This is likely due to DiMan’s more extended binding profiles over Fuc with DC-SIGN CRD, allowing DiMan to exploit not only the CRD’s primary (via coordination to the Ca^2+^ ion via its 3,4-OH groups, the same as Fuc)^11,49^ but also secondary binding sites (via hydrogen bonding to Ser360 and Glu358),^11^ alongside the ability of forming further van der Waals interactions with residues lying outside the primary binding pocket, such as Phe313, giving rise to a greater MLGI affinity enhancement.^11^ While Fuc has previously been identified to be a binding ligand for DC-SIGN, most previous studies have been based on DC-SIGN binding to Fuc containing oligosaccharides, such as the Lewis glycans and blood group A/B antigens,^44,49,59–61^ not the monosaccharide Fuc itself. Here, we have demonstrated that a polyvalent display of the monosaccharide Fuc alone can produce a strong DC-SIGN binder with apparent *K*_d_s as low as 3.9 nM. This affinity is considerably stronger (∼9 fold) than that of G5-EG_2_-Man (apparent *K*_d_ = 33 ± 2 nM),^37^ its monosaccharide mannose ligand coated G5 counterpart with identical LA-anchoring group and EG_2_ linker length. This finding is consistent to the literature result that DC-SIGN binds more strongly to Lewis-glycans than to oligomannose in glycan microarrays.^44^
(4) Increasing the EG_n_ linker length generally weakens G5-glycan’s MLGI affinity for DC-SIGN (e.g., *K*_d_ = 1.9 vs. 3.5 vs. 5.8 nM for G5-/EG_2_-, EG_6_- and EG_12_- DiMan, LGMR = 1000, respectively). The decrease in apparent binding affinity with the increasing linker length is not unexpected, due to the longer EG_n_ linkers causing a more flexible and disordered display of terminal glycans which have more conformational and rotational degrees of freedom. Hence there is a greater entropic penalty upon DC-SIGN binding,^36^ reducing the overall favorable Gibbs free energy change (ΔG). The only exceptions are G5-DiMans prepared at the lowest LGMR of 300, where their DC-SIGN affinities are almost independent of EG_n_ linker length. This result might be due to the presence of both simultaneous tetravalent binding and cross-linking interactions for G5-EG_2_/EG_6_-DiMan, but mainly simultaneous tetravalent binding for G5-EG_12_-DiMan as indicated by the *D*_h_ histograms of the formed complexes (see Figure 3).
(5) Varying glycan density (within the range studied here) does not significantly impact G5-glycans’ binding affinity with DC-SIGN. This is likely due to the fact that the glycan densities in all G5-glycans here (from 0.57 to 2.9 nm^2^/glycan, see SI, Table S2) are above the minimal glycan density threshold (∼7 nm^2^/per glycan, assuming all flexibly presented glycans beneath the footprint of a 3 nm spherical CRD are available for binding)^39^ required to form multivalent binding with DC-SIGN CRDs, making their DC-SIGN binding strong and comparable. While this assumption may not be true for G5-EG_2_-glycans which have the shortest linker, in that case, their suboptimal binding ΔH terms (due to <4 CRDs engaged in binding) are compensated by a notable reduction of entropic penalty (i.e., less negative ΔS), leading to comparable overall binding ΔG values and hence affinities (ΔG = RT ln­(*K*_*d*_), see later thermodynamic section, Table 2).

**Table 1:** Summary of the Apparent Binding *K*_d_ and QE_max_ of DC-SIGN Binding to Various G5-EG_n_-glycan Conjugates at 22 °C^a^

	*K*_d_ (nM)			QE_max_ (%)		
	LGMR = 1000	LGMR = 500	LGMR = 300	LGMR = 1000	LGMR = 500	LGMR = 300
G5-EG_2_-DiMan	1.9 ± 0.4	2.0 ± 0.5	4.3 ± 0.8	87.5 ± 0.5	71.6 ± 1.3	70.1 ± 1.0
G5-EG_6_-DiMan	3.5 ± 0.7	2.1 ± 0.7	3.8 ± 0.5	75.3 ± 1.6	70.2 ± 1.4	67.8 ± 1.1
G5-EG_12_-DiMan	5.8 ± 1.0	3.7 ± 0.8	4.7 ± 0.4	64.2 ± 3.4	50.9 ± 1.2	68.5 ± 0.9
G5-EG_2_-Fuc	4.4 ± 0.2	5.6 ± 0.4	5.4 ± 1.0	81.6 ± 0.7	77.0 ± 1.2	72.3 ± 0.8
G5-EG_6_-Fuc	3.9 ± 0.6	4.1 ± 0.6	6.0 ± 0.6	69.8 ± 2.1	79.4 ± 1.2	67.8 ± 1.1
G5-EG_12_-Fuc	13.9 ± 3.4	7.9 ± 1.9	11.6 ± 2.6	57.6 ± 3.0	59.4 ± 3.2	53.7 ± 0.3

a Data were derived from Figure 4 and Figures S31–S33.

**Table 2:** Summary of the Standard MLGI Thermodynamic Parameters for DC-SIGN Binding with G5-glycans of Varying EG_n_ Linker Lengths Prepared at Different LGMRs (1000, 500, and 300) at 298 K^a^

	Δ*H*° (kJ mol^–1^)			Δ*S*° (J K^–1^ mol^–1^)			Δ*G*° (kJ mol^–1^)		
	LGMR = 1000	LGMR = 500	LGMR = 300	LGMR = 1000	LGMR = 500	LGMR = 300	LGMR = 1000	LGMR = 500	LGMR = 300
G5-EG_2_-DiMan	–97 ± 3	–95 ± 3	–71 ± 1	–162 ± 8	–158 ± 100	–82 ± 5	–49 ± 4	–48 ± 30	–47 ± 2
G5-EG_6_- DiMan	–109 ± 12	–112 ± 2	–109 ± 8	–209 ± 41	–211 ± 77	–210 ± 25	–47 ± 17	–49 ± 23	–47 ± 11
G5-EG_12_- DiMan	–126 ± 2	–118 ± 1	–109 ± 6	–271 ± 8	–239 ± 36	–210 ± 18	–46 ± 3	–47 ± 11	–46 ± 8
G5-EG_2_-Fuc	–105 ± 3	–86 ± 17	–48 ± 4	–195 ± 10	–132 ± 58	–5 ± 13	–47 ± 4	–46 ± 25	–47 ± 5
G5-EG_6_-Fuc	–77 ± 4	–111 ± 10	–96 ± 13	–101 ± 13	–210 ± 33	–168 ± 42	–47 ± 6	–49 ± 14	–46 ± 18
G5-EG_12_-Fuc	–137 ± 1	–125 ± 15	–139 ± 1	–314 ± 4	–269 ± 40	–318 ± 3	–44 ± 2	–45 ± 21	–44 ± 1

a SDs represent fitting errors.

Previously, we have probed the MLGI thermodynamics of DC-SIGN binding with QD-DiMan (∼4 nm CdSe/ZnS core/shell quantum dots coated with DHLA-EG_11_-DiMan)^27^ or Gx-DiMan (x = 5, 13, and 27 nm GNPs coated with the LA-EG_4_-DiMan ligand)^38^ by measuring their temperature-dependent *K*_d_s via a QD-FRET (Förster resonance energy transfer) or GNP based NSET (nano surface energy transfer) readout followed by van’t Hoff analysis of the ln­(*K*_d_)–(1/T) plots.^27,38^ We have revealed that DC-SIGN binding with QD/Gx-DiMan is enthalpy-driven with a standard binding enthalpy change, ΔH°, of ∼ – 100 kJ/mol, approximately four times that of the monovalent binding (ΔH_mono_° = – 25.8 kJ/mol),^58^ indicating that all four CRDs in each DC-SIGN molecule are engaged in binding to the QD/GNP-DiMan.^27,38^ Furthermore, the unfavorable binding entropic change, ΔS°, was found to decrease with the increasing GNP size, attributed to larger scaffolds having a higher proportion of unbound free surface ligands that have retained their conformational and rotational degrees of freedom.^38^ The lesser entropic penalty (less negative ΔS°) can explain DC-SIGN’s stronger affinity for the larger GNPs over the smaller ones bearing the same glycan.^38^

To investigate the impact of linker length and glycan density on DC-SIGN MLGI thermodynamic parameters, we further measured the apparent *K*_d_s for DC-SIGN binding with G5-EG_n_-glycans (prepared at LGMRs of 1000, 500 and 300) under three different temperatures (e.g., 22, 27, and 32 °C). We then applied the van’t Hoff analysis (eq 5) to derive their binding thermodynamics by combining the two Gibbs free energy eqs (eqs 3 and 4):^27,38^

3 $$\Delta G^{{}^{\circ}}=\Delta H^{{}^{\circ}}-T\Delta S^{{}^{\circ}}$$

4 $$\Delta G^{{}^{\circ}}=-RT\;\ln\left(K_{a}\right)=RT\;\ln\left(K_{d}\right)$$

5 $$\ln\left(K_{d}\right)=\frac{\Delta H^{{}^{\circ}}}{R}\frac{1}{T}-\frac{\Delta S^{{}^{\circ}}}{R}$$

where ΔG° is the change of the binding Gibbs free energy, *K*_a_ is the equilibrium association constant, *K*_d_ is the equilibrium dissociation constant (where *K*_a_ = 1/*K*_d_), T is the absolute temperature in degrees Kelvin, and R is the ideal gas constant.

Figure 5A-B show the van’t Hoff plots and linear fits of the ln­(*K*_d_) - (1/T) relationships for the DC-SIGN binding with G5-glycan (LGMR = 1000). The ln­(*K*_d_) - (1/T) plots for DC-SIGN binding with G5-glycans made at LGMRs of 500 and 300 are given in SI, Figures S34–35. The slope and intercept obtained from the linear fits correspond to the (ΔH°/R) and (-ΔS°/R) terms, respectively, allowing us the derive the ΔH° and ΔS° values of DC-SIGN binding with various G5-glycans. The MLGI thermodynamic parameters obtained for DC-SIGN binding to G5-glycans (LGMR = 1000) are shown in Figure 5C–D. The detailed thermodynamic parameters for DC-SIGN binding with G5-EG_n_-glycans prepared at all three LGMRs are summarized in Table 2.

**Figure 5:**
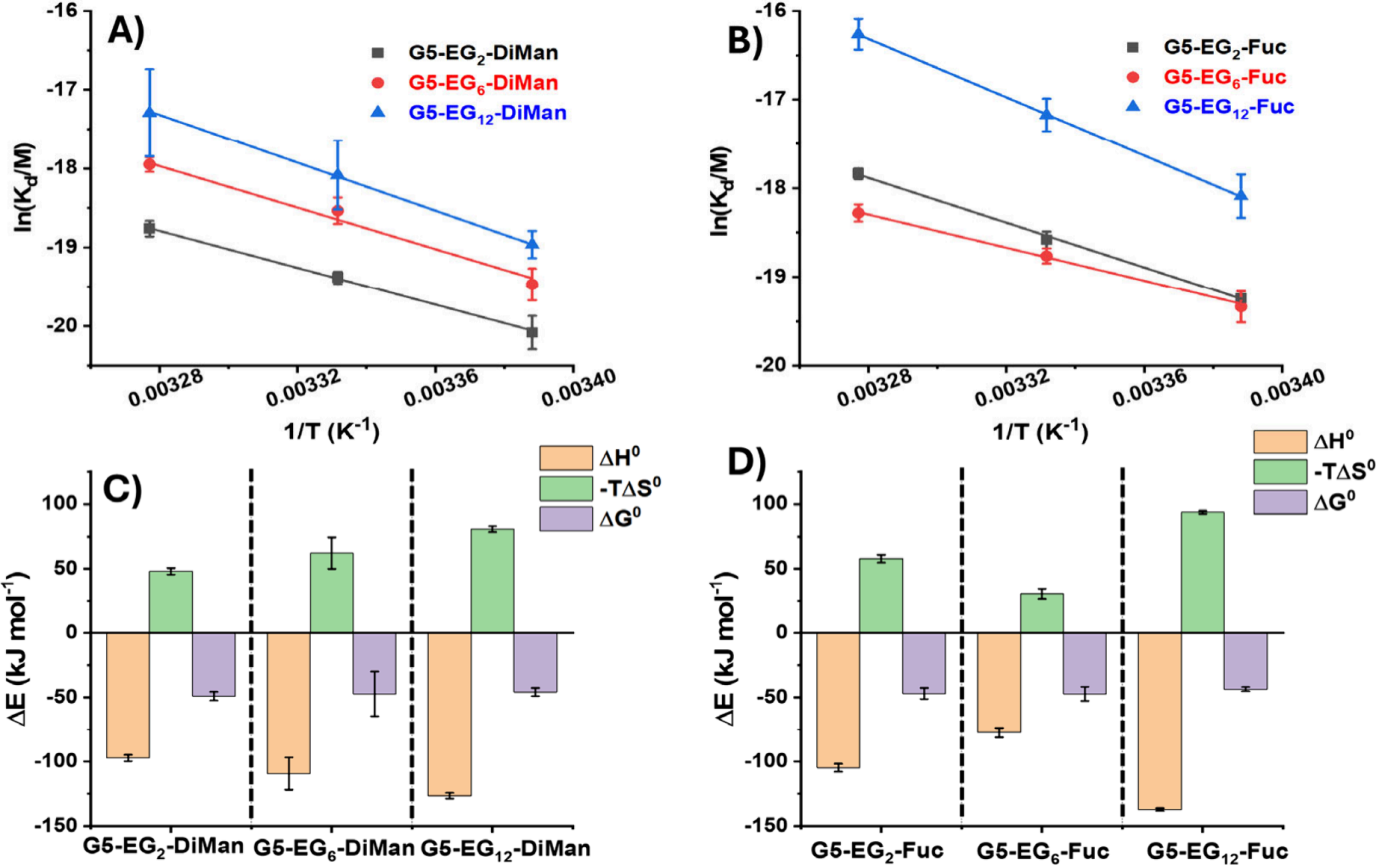
van’t Hoff analyses of the ln­(*K*_d_)–(1/*T*) relationships for DC-SIGN binding with G5-EG_n_-glycans prepared at a LGMR of 1000: (A) G5-EG_2_-DiMan (gray), G5-EG_6_-DiMan (red), and G5-EG_12_-DiMan (blue) or (B) G5-EG_2_-Fuc (gray), G5-EG_6_-Fuc (red), and G5-EG_12_-Fuc (blue). (C) Comparison of the standard enthalpy (orange), entropy (green), and Gibbs free energy (purple) changes of G5-EG_n_-DiMan (LGMR = 1000) binding with DC-SIGN at 298 K. (D) Comparison of the standard enthalpy (orange), entropy (green), and Gibbs free energy (purple) changes of G5-EG_n_-Fuc (LGMR = 1000) binding with DC-SIGN at 298 K. SDs represent fitting errors.

Three notable conclusions can be drawn from the results shown in Table 2.

(1) G5-EG_n_-glycan conjugates binding with DC-SIGN were found to be thermo-dynamically favorable (with large negative binding ΔG° values) and exhibited both negative binding ΔH° and ΔS° values, indicating favorable binding enthalpy but unfavorable entropy terms. Therefore, their bindings are all enthalpically driven. Their binding ΔG° values are similar, all within error of each other, indicating the overall energy release from binding is not drastically affected by linker length, glycan density or glycan identity under our experimental conditions.
(2) Most of the G5-EG_n_-glycans binding with DC-SIGN display a ΔH° value of around 4-fold that of the corresponding monovalent binding measured by ITC (−25.6 kJ mol^–1^),^58^ indicating all four CRDs in each DC-SIGN molecule are engaged in binding to G5-glycans. At LGMR of 300, however, the G5-EG_2_-glycans binding with DC-SIGN display a ΔH° value of −71 or −48 kJ mol^–1^ for DiMan and Fuc, respectively, implying that fewer than four DC-SIGN CRDs might be engaged in binding. Here, the density of the glycans (with the shortest EG_2_ linker) on the G5 surface may be too sparse to effectively engage all four CRDs in one DC-SIGN molecule simultaneously. For G5-EG_n_-glycans at LGMR 300 with longer linkers (EG_6_ and EG_12_), their DC-SIGN binding ΔH° values returned to ∼ −100–140 kJ mol^–1^, suggesting that the longer and more flexible linker can adapt well to bind all four CRDs of one DC-SIGN successfully, even allowing for enthalpically enhanced binding where NΔH°_mono_ < ΔH°_poly_ (where N is the number of interactions). This result matches that of the binding mode studies described in the previous section, where G5-EG_2_-DiMan (LGMR 300) was found to cross-link with DC-SIGN molecules to form large scale G5-lectin assemblies, indicating the existence of bi- or tri- valent binding for DC-SIGN molecules under such conditions. Moreover, the relative abundance of the cross-linked to monolayer species is reduced with the increasing EG_n_-linker length (see Figure 3).
(3) For G5-glycan based MLGIs engaging DC-SIGN’s all four CRDs, their binding ΔS° values generally became increasingly more negative with the increasing EG_n_ linker length, indicating the increased entropic penalty in constraining such more flexible and hence more disordered linkers upon binding to DC-SIGN. This result is consistent with, and likely the cause of, the weaker affinities observed for G5-glycans with the longer EG_12_-linkers over the shorter, less flexible EG_2_-linker, as their ΔH° values are similar across the different linker lengths. When fewer than four CRDs in one DC-SIGN are engaged in binding as indicated by their ΔH° values, their ΔS° value is less negative than those engaging all 4 CRDs, due to the lower entropic penalty of constraining only 2 or 3 linkers per DC-SIGN molecule.

Relating to the last two points, the weaker binding ΔH° observed for the G5-EG_2_-glycans at the lowest glycan densities was offset by the lower entropic penalty associated with binding fewer linkers per G5-EG_2_-glycan. As a result, the overall binding affinities remained relatively constant across all G5-glycans prepared under different LGMRs, despite their distinct underlying thermodynamic profiles. This highlights the importance of detailed investigation into the mechanisms behind MLGIs, as the binding affinity alone would suggest little impact of changing valency. This result also shed light on how binding affinity is a fine balance between the binding ΔH° and ΔS° terms which are affected by both glycan density and linker flexibility.

Overall, G5-EG_2_-glycans made in LGMRs of 1000 and 500 show great consistency between apparent binding affinity, maximum enthalpy enhancement and minimum entropic cost, indicating that glycans with short linkers and a valency of ≥ 350 per G5 provides an optimal combination of spatial topology, linker entropy and local concentration for statistical rebinding to generate strong MLGI to DC-SIGN. To improve the potency of drugs and/or therapeutic interventions, it is important to enhance the drug–target binding affinity. This can be achieved by maximizing their favorable binding enthalpy terms while reducing unfavorable entropic terms. Our results show that creating a suitable polyvalent display of glycans with a suitable short linker which is well spatially matched to target lectin’s binding sites could be a suitable solution.

#### Blocking DC-SIGN/R-Mediated Augmentation of Ebola Virus Glycoprotein-Driven Transduction

The above MLGI studies between G5-glycans and DC-SIGN were all performed in a buffer solution, which is different from DC-SIGN’s native environment on dendritic cell membranes. To investigate how DC-SIGN-G5-glycan solution binding behaviors are correlated to those on cell membranes, a cellular viral inhibition assay was conducted. The strong affinities of the G5-glycans with DC-SIGN suggests that they should be able to bind strongly to DC-SIGN molecules on cell surfaces, effectively blocking DC-SIGN from binding to viral glycoproteins and augmentation of viral entry into host cells. Here, a second tetrameric lectin viral receptor, DC-SIGNR,^41^ was also employed as a control lectin to investigate their antiviral selectivity. DC-SIGN and DC-SIGNR (collectively referred to as DC-SIGN/R) are closely related, sharing 77% amino acid homology, and have almost identical tetrameric architecture and monovalent CRD-mannose binding motifs.^44,62^ Despite close similarity in binding to mannose containing glycans, only DC-SIGN, but not DC-SIGNR, was found to bind strongly to fucose containing glycans in glycan microarrays.^44^

To investigate whether G5-glycans (prepared at a LGMR of 500 as they show the highest consistency in DC-SIGN binding behaviors) can effectively block DC-SIGN/R- mediated viral entry into host cells, human embryonic kidney 293T cells were transfected to express DC-SIGN or DC-SIGNR on their surface. Single cycle vesicular stomatitis virus (VSV) particles encoding the luciferase gene and bearing the Ebola virus surface glycoprotein (EBOV-GP) were used to model Ebola virus entry into the transfected cells. VSV particles bearing the vesicular stomatitis virus glycoprotein (VSV-G) were employed as a specificity control.^37–39^ The specific binding of the EBOV-GP to cell surface DC-SIGN/R receptors should promote viral attachment and entry into host cells, as indicated by enhanced luciferase signals. Binding high-affinity G5-glycans to cell surface DC-SIGN/R receptors should prevent them from being able to bind EBOV-GP, thereby blocking virus entry into host cells. We have previously demonstrated that this cellular model for viral entry is highly robust.^37–39^

VSV/EBOV-GP particles were added to mock treated cells (293T cells transfected with empty plasmid) or 293T cells transfected with either DC-SIGN or DC-SIGNR encoding plasmid. As expected, cells transfected to express DC-SIGN/R showed a 10+ fold increase in luciferase activity compared to the mock cells, indicating VSV/EBOV-GP entry into cells was significantly enhanced by DC-SIGN/R. Mock cells still showed a small increase in luciferase activity compared to those not exposed to VSV/EBOV-GP particles, as expected since DC-SIGN augments but is not essential for EBOV-GP-driven entry into 293T cells. Moreover, VSV/VSV-G particles, which cannot employ DC-SIGN/R for augmentation of cell entry, were unaffected by DC-SIGN/R transfection as expected (see SI, Figure S36).

The raw viral inhibition data are presented in SI, Figures S37–S38. All G5-glycans significantly and dose-dependently inhibited DC-SIGN-dependent augmentation of VSV/EBOV-GP entry, while only G5-DiMan, but not G5-Fuc, significantly inhibited DC-SIGNR-dependent viral entry. This is not unexpected, because G5-EG_2_-Fuc (prepared at a LGMR of 500) gives no measurable specific interactions with DC-SIGNR assessed by the GNP fluorescence quenching assay. It gives the same low levels of QEs as the G5-EG_2_–OH negative control (G5 coated with the LA-EG_2_–OH ligand) showing no specific binding with DC-SIGN/R,^39^ across the whole range of concentrations studied (SI, Figure S39). This result is consistent with literature that Fuc (presented in multivalent glycan microarrays) does not bind to DC-SIGNR, while DiMan binds to both DC-SIGN and DC-SIGNR.^44^ The normalized viral inhibition data were well fitted by a logarithmic dose response model (eq 6) and shown in Figure 6.

6 $$\mathrm{NA}=\frac{1}{1+10^{\left(\log IC_{50}-\log C\right)p}}$$

where NA is the luciferase activity normalized to the corresponding control collected in the absence of the G5-glycans, *IC*_*50*_ is the concentration giving 50% inhibition, *C* is the G5-glycan concentration, and *p* is the Hill slope.

**Figure 6:**
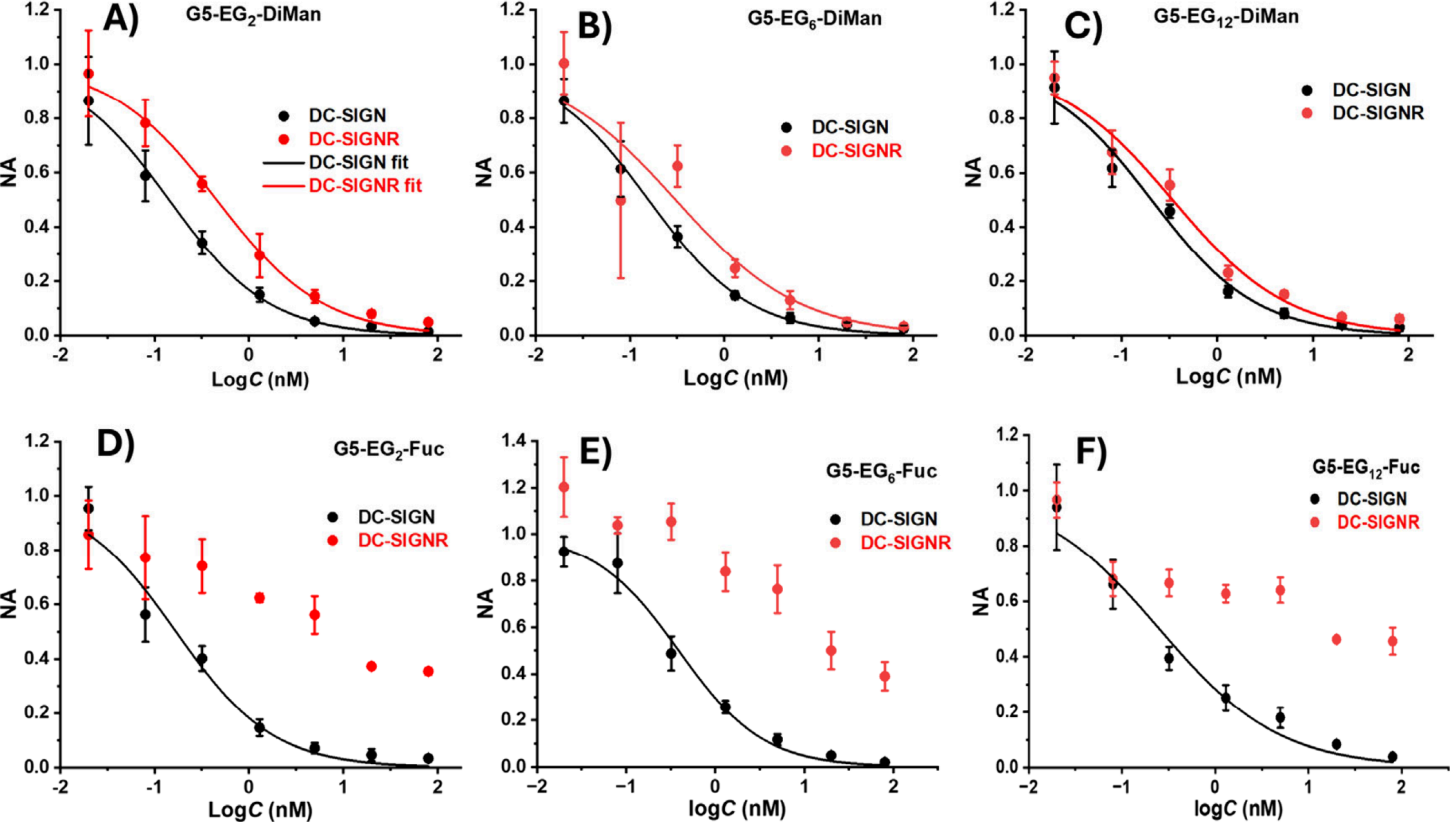
Plot of normalized luciferase activities of DC-SIGN- or DC-SIGNR-expressing 293T cells after treatment with varying concentrations of (A) G5-EG_2_-DiMan, (B) G5-EG_6_-DiMan, (C) G5-EG_12_-DiMan, (D) G5-EG_2_-Fuc, (E) G5-EG_6_-Fuc, and (F) G5-EG_12_-Fuc as inhibitors to block VSV/EBOV-GP cell entry. Graphs were fitted by eq 6, and error bars represent the standard deviations (SDs) of technical quadruplicates at each concentration. Experiments were repeated twice, and data from one representative experiment are shown.

While the *IC*_*50*_ value is important in determining the efficacy of viral inhibition, the Hill slope *p* is also of great importance: it determines how quickly an inhibitor can achieve complete inhibition with the increasing *C*. For example, if two inhibitors have the same *IC*_*50*_ but different *p* values of 0.5 or 1, respectively, then the inhibitor concentration required to achieve 99% inhibition will be 9801- or 99- times the *IC*_*50*_ value, respectively. Therefore, an inhibitor with *p* = 0.5 would be much less effective than that with *p* = 1, requiring almost 100-fold higher concentrations to achieve the same 99% inhibition despite having the same *IC*_*50*_ value.

G5-DiMan were able to effectively block both DC-SIGN and DC-SIGNR mediated viral entry while G5-Fuc only effectively blocked DC-SIGN-, but not DC SIGNR-, dependent augmentation of viral entry, even at high concentrations. This result is fully consistent with literature that DC-SIGN binds strongly to both mannose- and fucose- containing glycans, DC-SIGNR however, only binds strongly to mannose-, but not fucose-, containing glycans.^44^ Importantly, compared to VSV/EBOV-GP, luciferase activities of the control VSV/VSV-G particles which do not use DC-SIGN/R for cell entry, were not significantly affected by G5-glycan treatment, confirming that the observed inhibitory effects were specific (SI, Figures S37/38). Furthermore, all G5-glycans were found to exhibit no measurable cytotoxicity against 293 T cells across the concentration range used in antiviral studies, confirming that the observed viral inhibition effects are not artifacts of cellular toxicity (SI, Figure S40). The *IC*_*50*_, *p*, and *R* ^*2*^ values from the best fits for each G5-glycan are summarized in Table 3.

**Table 3:** Summary of the Inhibition Data for G5-glycans (LGMR = 500) against DC-SIGN/R-Mediated EBOV-GP-Driven Infection of 293T Cells^a^

	DC-SIGN			DC-SIGNR		
G5-EG_n_-glycan	IC_50_ (nM)	*p*	*R* ^2^	*IC*_50_ (nM)	*p*	*R* ^2^
EG_2_-DiMan	0.14 ± 0.008	0.82 ± 0.04	0.940	0.46 ± 0.04	0.77 ± 0.05	0.985
EG_6_-DiMan	0.16 ± 0.009	0.81 ± 0.04	0.973	0.31 ± 0.13	0.66 ± 0.17	0.959
EG_12_-DiMan	0.21 ± 0.03	0.79 ± 0.08	0.974	0.34 ± 0.06	0.72 ± 0.08	0.967
EG_2_-Fuc	0.17 ± 0.03	0.84 ± 0.13	0.957	–	–	–
EG_6_-Fuc	0.39 ± 0.05	0.92 ± 0.09	0.994	–	–	–
EG_12_-Fuc	0.25 ± 0.05	0.67 ± 0.09	0.982	–	–	–

a Errors represent the SDs of the fitting.

While G5-EG_n_-DiMan were able to effectively block both DC-SIGN and DC-SIGNR-dependent cell entry, their activity against DC-SIGNR-dependent viral entry was weaker (indicated by higher *IC*_*50*_ values). These results are consistent with our previous data showing that mannose based G5-glycans bind more strongly to DC-SIGN than to DC-SIGNR,^36–38^ due to different orientations of their four CRDs.^36^ In DC-SIGN, all four CRDs face upwardly, hence one G5-glycan can simultaneously bind to all 4 CRDs in each DC-SIGN, completely blocking their ability to interact with EBOV-GPs on the virion surface to mediate viral entry. In contrast, the four outwardly positioned CRDs in each DC-SIGNR molecule only allow each G5-DiMan to bind divalently via two outward facing binding sites, making it difficult to fully block all cell surface DC-SIGNR receptors from binding to viral surface EBOV-GPs to achieve complete inhibition.^37^

Interestingly, the inhibition potency of G5-EG_n_-DiMan against DC-SIGN-dependent viral entry showed a degree of dependence on the linker length, where their *IC*_*50*_ values were found to increase (become less potent) with the increasing EG_n_ linker length. This result correlates well with their relative DC-SIGN affinities measured by GNP based NSET quenching experiment described in the previous section, where increasing the EG_n_ linker length in G5-EG_n_-DiMan generally results in a weaker DC-SIGN binding affinity. Alongside this, a decrease of the Hill slope *p* was also observed for G5-EG_n_-DiMan with the increasing EG_n_ linker length, indicating markedly higher inhibitor concentration is required to completely block viral inhibition.

All G5-Fuc glycans strongly inhibited viral entry mediated by DC-SIGN as expected from their strong DC-SIGN binding affinity described in the previous section. Unexpectedly, the *IC*_*50*_ for G5-EG_6_-Fuc was found to be slightly higher (less potent) than that of G5-EG_12_-Fuc (0.39 ± 0.05 vs. 0.25 ± 0.05 nM) despite a stronger apparent DC-SIGN binding affinity (*K*_d_ 4.1 ± 0.6 vs. 7.9 ± 1.9 nM). A careful examination revealed that the lack of inhibition at the two lowest concentrations for G5-EG_6_-Fuc (Figure 6E) was responsible for this discrepancy. However, after considering the Hill slope, the concentrations required to achieve a 99% inhibition for G5-EG_n_-Fuc were calculated to follow a single pattern with the increasing EG_n_ linker length (IC_99_ = 40, 58, and 238 nM for EG_2_, EG_6_ and EG_12_, respectively). This result further highlights the importance of considering both the IC_50_ and the Hill slope *p*. Importantly, Fuc coated G5s showed minimal inhibition to DC-SIGNR mediated viral transduction (Figure 6D-F). This result is not unexpected because G5-EG_2_-Fuc shows no measurable specific interactions with DC-SIGNR (SI, Figure S39) and is fully consistent with the previous literature results.^44^

While *IC*_*50*_ and *K*_*d*_ values cannot be compared directly as they do not describe the same thing and were obtained under different binding environments, on cell membrane vs. in buffer solution. Nevertheless, a positive correlation between *IC*_50_ and *K*_d_ was observed, where a lower *K*_d_ generally equaled a lower *IC*_*50*_, and their relative values for the shorter (EG_2_, EG_6_) and longer (EG_12_) linkers showed the same fold differences. For example, G5-EG_2_/_6_-DiMan exhibited 1.4–2-fold stronger affinity with DC-SIGN (lower *K*_d_) than G5-EG_12_-DiMan and the IC_50_ of EG_2/6_-DiMan is ∼ 1.5-fold lower (stronger inhibitor) than G5-EG_12_-DiMan. Furthermore, G5-EG_n_-DiMans were found to be both stronger DC-SIGN binders and better inhibitors against DC-SIGN mediated viral infection than their G5-EG_n_-Fuc counterparts. Overall, the excellent potency of the G5-EG_n_-glycans, especially for the ones with the shortest EG_2_ linker, in blocking DC-SIGN mediated augmentation of EBOV-GP-dependent viral entry is highly promising. Their impressive, sub-nM *IC*_*50*_ values (0.14 ± 0.01 and 0.17 ± 0.03 nM for G5-EG_2_-DiMan and Fuc, respectively) are comparable or even better than some of the most potent glycoconjugate inhibitors against DC-SIGN-mediated pseudo-Ebola virus entry of host cells reported in literature, including the giant globular multivalent glycofullerenes (*IC*_50_: 0.67 nM),^63^ the virus-like glycodendrinanoparticles (*IC*_50_: 0.91 nM)^64^ and the QD-EG_3_-DiMan reported by our group (*IC*_50_: 0.70 ± 0.2 nM).^36^ This is important because glycoconjugate based viral entry inhibitors can offer two key advantages over other antiviral approaches. First, they can prevent viral mutations by blocking the virus from entering host cells. Second, their antiviral effectiveness is less likely to be affected by viral mutations than other antiviral strategies, since viral surface glycans are mostly conserved across different viral variants.^65^ Therefore, the G5-EG_n_-glycans reported herein, with their excellent potency in blocking DC-SIGN-mediated EBOV-GP driven viral infection of host cells, appear to be a potential promising antiviral agent against a range of viruses exploiting DC-SIGN for host cell entry.

## Conclusions

In summary, by exploiting the versatile robust gold–thiol chemistry, tunable architecture and powerful fluorescence quenching properties of GNPs, we have developed polyvalent glycan-GNPs as biophysical probes to investigate how glycan type, linker lengths and surface glycan densities control their MLGI behaviors with DC-SIGN. Using the GNP based NSET fluorescence quenching assay, we have shown that binding of DC-SIGN with G5-EG_n_-glycans is enthalpy driven, with large negative binding Δ*H* ^0^ values of ∼ 4 times that of the corresponding monovalent binding, suggesting that all four CRDs in each DC-SIGN molecule are engaged in binding to one G5-glycan at relatively high glycan valencies (i.e., ≥ 350 per G5). Moreover, we have revealed that increasing EG_n_ linker length (from EG_2_ to EG_12_) weakens G5-glycan’s MLGI affinities with DC-SIGN, due to an increased entropic penalty. Interestingly, at lowest glycan densities (≤180 glycans per G5), G5-glycans with the shortest EG_2_ linkers give significantly lower negative ΔH° values, being ∼ 2–3 times that of the corresponding monovalent binding, suggesting that this G5-glycan architecture presents a mismatch with the binding surface of tetrameric DC-SIGN. Nevertheless, their binding ΔH° value was returned to ∼ 4 times that of the monovalent binding as linker length is increased to EG_12_, highlighting how linker flexibility can allow for deviation from spatial constraints of the DC-SIGN CRDs. This result agrees well with the binding mode studies, where dominant cross-linking binding (indicative of <4 DC-SIGN CRDs engaged in binding to one G5-glycan) was observed for G5-DiMan with the lowest density and shortest linker length, while dominant DC-SIGN monolayer binding species (indicative of all 4 CRDs binding to one G5-glycan) were observed for all other G5-EG_n_-glycans. Interestingly, a reduction of favorable binding ΔH° term was found to be compensated by a reduction of unfavorable binding ΔS° term (and vice versa), giving rise to a very similar overall binding ΔG° value (hence overall *K*_d_, as ΔG° = RT ln­(*K*_*d*_)). Overall, this work has revealed useful rules for designing glycoconjugates for targeting multivalent lectins. First, reducing glycan flexibility (via short, less flexible linkers) can reduce binding entropic penalty, thereby enhancing affinity. However, to retain optimal binding enthalpy, glycans presented on rigid linkers must match closely the spatial organization of lectin’s binding sites. Second, lowering glycan density reduces the ability of glycans to simultaneously bind to all CRDs in one lectin, but this can be reinstalled by introducing long flexible linkers. Therefore, finding the right balance between enthalpic and entropic factors is vital.

Finally, we have demonstrated that G5-EG_n_-DiMans are highly potent inhibitors against both DC-SIGN and DC-SIGNR mediated pseudo-Ebola virus cellular infection with sub-nM level *IC*_*50*_ values, whereas G5-EG_n_-Fucs are efficient only against DC-SIGN, but not DC-SIGNR, mediated viral cellular entry. Their viral inhibition data showed good consistency with their solution MLGI affinities, where stronger binding (lower apparent *K*_d_) equated to a stronger inhibitor (lower *IC*_*50*_). This trend, however, was reversed for inhibiting DC-SIGNR-mediated viral infection where G5-glycans with a longer linker tended to display a stronger inhibition. This is likely due to the longer and more flexible linkers afford the terminal glycans better adaptivity, allowing them to bind simultaneously to four outwardly facing CRDs in one DC-SIGNR, making it a more effective antiviral agent. This result highlights the importance of tailoring nanoparticle design for targeting specific lectins.

## Experimental Section

### 8-Azido-3,6-dioxaoctyl-2,3,4-tri-*O*-acetyl-α-l-fucopyranoside (N_3_-EG_2_-FucOAc)

L-Fuc (1.0 g, 6.08 mmol), 2-[2-(2-chloroethoxy)­ethoxy]­ethanol (4.4 mL, 30.4 mmol), PPh_3_ (158 mg, 0.6 mmol) and CBr_4_ (200 mg, 0.6 mmol) were placed in a 10 mL round-bottom flask. The mixture was heated at 65 °C for 4 h. The reaction was then cooled to room temperature (RT) and loaded on a short silica gel column to remove the excess of acceptor (EtOAc, then EtOAc:MeOH = 10:1). The crude fucoside was subjected to acetylation with Ac_2_O (10 mL) and pyridine (10 mL). The reaction was stirred at RT overnight then concentrated in vacuo and dissolved in EtOAc (80 mL). The organic layer was washed with H_2_O (40 mL), 1 M HCl (40 mL), saturated NaHCO_3_ (40 mL) and brine (40 mL). The organic layer was dried over Na_2_SO_4_, filtered and concentrated in vacuo. The crude mixture (1.5 g, 3.41 mmol) was then subjected to azidation by adding NaN_3_ (1.11 g, 17.0 mmol), nBu_4_N^+^ I^–^ (125 mg, 0.34 mmol) and DMF (12 mL) and stirring at 65 °C overnight. The reaction mixture was concentrated in vacuo to remove the DMF then diluted with EtOAc (80 mL) and washed with saturated NaHCO_3_ (40 mL) and the aqueous layer was extracted with EtOAc (3 × 20 mL). The combined organic extracts were dried over Na_2_SO_4_, filtered and concentrated in vacuo. The crude product was then purified by flash column chromatography (silica, PE: CHCl_3_: CH_3_(C = O)–CH_3_ = 7:2:1.2) to afford the desired N_3_-EG_2_-α-fucosideOAc (750 mg, 1.60 mmol, 26% overall yield after three synthetic steps): ^1^H NMR (501 MHz, CDCl3) (α anomer) δ 5.41 – 5.33 (m, 1H, H-2), 5.29 (dd, *J* = 3.4, 1.3 Hz, 1H, H-4), 5.14 – 5.07 (m, 2H, H-3, H-1), 4.22 (qd, *J* = 6.5, 1.3 Hz, 1H, H-5), 3.85 – 3.72 (m, 1H, -OCH_2_−), 3.70 – 3.60 (m, 9H, -OCH_2_−), 3.39 (t, *J* = 5.4 Hz, 2H, CH2N3), 2.15 (s, 3H, COCH_3_), 2.06 (s, 3H, COCH3), 1.97 (s, 3H, COCH3), 1.13 (d, *J* = 6.6 Hz, 3H, CH3). ^13^C NMR (126 MHz, CDCl3) δ 170.78­(C = O), 170.58 (C = O), 170.20 (C = O), 96.38­(C-1), 71.34­(C-4), 70.89 (OCH_2_), 70.86­(OCH_2_), 70.38­(OCH_2_), 70.24­(OCH_2_) 68.30­(C-3), 68.15­(C-2), 67.61­(OCH_2_), 64.45­(C-5) 50.80­(CH_2_N_3_) 29.98­(COCH_3_), 20.85­(COCH_3_), 20.80­(COCH_3_), 15.97­(C-6). MS: calcd *m*/*z* for C_18_H_29_N_3_O_10_Na (M + Na)^+^ 470.18; found 469.93.

### 8-Azido-3,6-dioxaoctyl-*O*-α-l-fucopyranoside (Fuc-EG_2_-N_3_)

To a solution of α-azido fucosideOAc (370 mg, 0.83 mmol) in MeOH was added NaOMe and the reaction stirred at RT for 1 h. The reaction was then neutralized with amberlist H+ resin, filtered and concentrated in vacuo. Purification by flash column chromatography (silica, CH_2_Cl_2_:MeOH = 20:1 then 10:1) afforded the desired N_3_-EG_2_-α-Fuc (240 mg, 0.75 mmol, 90%) as a clear oil: ^1^H NMR (400 MHz, CDCl_3_) δ 4.90 (d, *J* = 3.3 Hz, 1H, H-1), 4.00 (q, *J* = 6.6 Hz, 1H, H-5), 3.90 – 3.75 (m, 4H, H-2, H-3, H-4, -OCH_2_−), 3.71 – 3.62 (m, 9H, -OCH_2_−), 3.40 (t, *J* = 5.2 Hz, 2H, CH_2_N_3_), 1.28 (d, *J* = 6.6 Hz, 3H, CH_3_). ^13^C NMR (101 MHz, CDCl_3_) δ 99.15­(C-1), 71.87, 71.53, 70.80­(OCH_2_), 70.63­(OCH_2_), 70.33­(OCH_2_), 70.18­(OCH_2_), 69.58, 67.64­(OCH_2_), 66.26­(C-5), 50.80­(CH_2_N_3_), 16.37­(C-6). HRMS: calculated *m*/*z* for C_12_H_23_N_3_O_7_Na (M+Na)^+^ 344.1428; found 344.1420

### General Protocol for Preparing LA-EG_n_-C≡CH Linkers by Amide Coupling:^37,65^

A solution of H_2_N-EG_n_-C≡CH (1 equiv), lipoic acid (1 equiv) and DMAP (0.2 equiv) in dry CH_2_Cl_2_ was cooled to 0 °C under an N_2_ atmosphere and stirred for 10 min. DCC (1.3 equiv) in dry CH_2_Cl_2_ was added dropwise and the reaction was stirred at 0 °C for another hour and then left to gradually return to RT and stirred for 24 h. The reaction mixture was filtered through Celite and the solid washed with CHCl_3_. The combined filtrate and washings were concentrated in vacuo and purification by flash column chromatography (silica, CH_2_Cl_2_:MeOH = 20:1) afforded the desired products as a yellow oils.

### Lipoamide-hexa­(ethylene glycol)-propargyl (LA-EG_6_-C≡CH)

Yield: 1.57g, 3.09 mmol, 99%; ^1^H NMR (400 MHz, CDCl_3_) δ 6.28 (s, 1H, NH), 4.20 (d, *J* = 2.4 Hz, 2H, CH_2_C≡CH), 3.72 – 3.58 (m, 18H), 3.58 – 3.52 (m, 3H), 3.44 (td, *J* = 5.6, 4.4 Hz, 2H), 3.22 – 3.06 (m, 2H), 2.50 – 2.41 (m, 2H), 2.22 – 2.16 (m, 2H), 1.96 – 1.84 (m, 4H), 1.77 – 1.61 (m, 4H), 1.52 – 1.39 (m, 2H). ^13^C NMR (101 MHz, CDCl_3_) δ 172.94­(C = O), 106.6­(CCH), 74.69­(CCH), 70.72, 70.56, 70.36, 70.11, 69.25, 58.55 (CH_2_CCH), 56.58­(CH), 40.38, 39.31, 38.61, 36.47, 34.82, 29.08, 25.53. MS: calcd *m*/*z* for C_23_H_42_NO_7_S_2_ (M+H)^+^ 508.24; found 508.21.

### Lipoamide-dodeca­(ethylene glycol)-propargyl (LA-EG_12_-C≡CH)

Yield: 262 mg, 0.34 mmol, 40%; ^1^H NMR (400 MHz, CDCl_3_) δ 6.37 (s, 1H, NH), 4.19 (d, *J* = 2.4 Hz, 2H), 3.71 – 3.57 (m, 44H), 3.54 (dd, *J* = 5.6, 4.4 Hz, 3H), 3.43 (q, *J* = 4.4 Hz, 2H), 3.21 – 3.05 (m, 2H), 2.49 – 2.40 (m, 2H), 2.22 – 2.16 (m, 2H), 1.90 (dq, *J* = 12.7, 7.0 Hz, 1H), 1.76 – 1.58 (m, 4H), 1.53 – 1.38 (m, 2H). ^13^C NMR (101 MHz, CDCl_3_) δ 172.94­(C = O), 79.67, 74.56­(CCH), 70.57, 70.52, 70.41, 70.21, 69.92, 69.11, 58.40 (CH_2_CCH), 56.44­(CH), 40.24, 39.22, 38.47, 36.25, 34.67, 28.93, 25.41. MS: calcd *m*/*z* for C_35_H_66_NO_13_S_2_ (M+H)^+^ 772.39; found 772.62.

### General Protocol for Preparing LA-glycan Ligands via Click Chemistry

To a 1:1 (v:v) THF: H_2_O solution (2.0–5.0 mL) containing the N_3_-EG_2_-glycan (1.1 equiv) and LA-EG_n_-C**≡CH** linker (1 equiv), was added CuSO_4_.5H_2_O (0.036 equiv), Tris­(benzyltriazolylmethyl)­amine (TBTA, 0.063 equiv) followed by sodium ascorbate (0.135 equiv) and the resulting solution was stirred at RT. After 3 h, TLC confirmed the complete consumption of all starting materials. The solvent was then evaporated, and the crude product was purified by size exclusion chromatography using Biogel P2 column and 20 mM ammonium formate solution as the eluent to afford the pure products.^37,65^

### LA-EG_2_-α-l-fucopyranoside (LA-EG_2_-Fuc)

Yield: 9.2 mg, 0.014 mmol, 15%; ^1^H NMR (400 MHz, D_2_O) δ 8.41 (s, 1H, NH), 8.02 (s, 1H, triazole-H), 4.80 (d, *J* = 3.9 Hz, 1H, Fuc-H1), 4.62 (s, 2H), 4.57 (t, *J* = 5.1 Hz, 2H), 4.01 – 3.94 (m, 1H), 3.90 (t, *J* = 5.1 Hz, 2H), 3.81 – 3.48 (m, 19H), 3.30 (t, *J* = 5.4 Hz, 2H), 3.11 (qt, *J* = 11.4, 6.4 Hz, 2H), 2.39 (dq, *J* = 12.3, 6.1 Hz, 1H), 2.16 (t, *J* = 7.2 Hz, 2H), 1.88 (dd, *J* = 13.2, 6.7 Hz, 1H), 1.64 (dq, *J* = 13.8, 7.3 Hz, 1H), 1.52 (hept, *J* = 7.5, 6.8 Hz, 2H), 1.31 (p, *J* = 7.7 Hz, 2H), 1.12 (d, *J* = 6.8 Hz, 3H). ^13^C NMR (101 MHz, D_2_O) δ 176.68 (C = O), 125.44­(CCH), 98.61­(Fuc-C1), 71.80, 69.73, 69.66, 69.58, 69.52, 69.46, 69.02, 68.91, 68.79, 68.08, 66.82, 66.59, 63.15, 56.57, 50.05, 40.27, 38.92, 38.15, 35.48, 33.76, 27.88, 25.07, 15.36. HRMS: calcd *m*/*z* for C_27_H_49_N_4_O_10_S_2_ (M+Na)^+^ 675.2710; found 675.2707.

### LA-EG_6_-α-l-fucopyranoside (LA-EG_6_-Fuc)

Yield: 28.3 mg, 0.034 mmol, 58%; ^1^H NMR (400 MHz, D_2_O) δ 8.12 (s, 1H, triazole-H), 4.89 (d, *J* = 3.9 Hz, 1H, Fuc-H1), 4.72 (s, 2H), 4.66 (t, *J* = 5.0 Hz, 2H), 4.07 (q, *J* = 6.7 Hz, 1H), 4.00 (t, *J* = 5.0 Hz, 2H), 3.89 – 3.60 (m, 33H), 3.40 (t, *J* = 5.3 Hz, 2H), 3.22 (qt, *J* = 11.2, 6.4 Hz, 2H), 2.49 (dq, *J* = 12.0, 5.9 Hz, 1H), 2.27 (t, *J* = 7.2 Hz, 2H), 1.99 (dq, *J* = 13.5, 6.8 Hz, 1H), 1.76 (dq, *J* = 13.5, 7.2 Hz, 1H), 1.70 – 1.56 (m, 3H), 1.42 (p, *J* = 7.6 Hz, 2H), 1.21 (d, *J* = 6.5 Hz, 3H). ^13^CNMR (101 MHz, D_2_O) δ 176.80­(C = O), 125.48­(CCH), 98.60­(Fuc-C1), 71.80, 69.71, 69.66, 69.60, 69.56, 69.49, 69.44, 68.94, 68.89, 68.77, 68.07, 66.81, 66.59, 63.10, 56.54, 50.04, 40.27, 38.91, 38.10, 35.48, 33.74, 27.87, 25.04, 15.32. HRMS: calcd *m*/*z* for C_35_H_64_N_4_O_14_S_2_ (M+Na)^+^ 851.3758; found 851.3782.

### LA-EG_12_-α-l-fucopyranoside (LA-EG_12_-Fuc)

Yield: 29.5 mg, 0.027 mmol, 69%; ^1^H NMR (400 MHz, D_2_O) δ 8.12 (s, 1H), 4.89 (d, *J* = 3.8 Hz, 1H, Fuc-H1), 4.71 (s, 2H), 4.66 (t, *J* = 5.0 Hz, 2H), 4.07 (q, *J* = 6.6, 5.9 Hz, 1H), 4.00 (t, *J* = 5.0 Hz, 2H), 3.87 (dd, *J* = 10.4, 3.2 Hz, 2H), 3.79 (s, 3H), 3.77 – 3.58 (m, 54H), 3.40 (t, *J* = 5.3 Hz, 2H), 3.30 – 3.15 (m, 1H), 2.57 – 2.45 (m, 1H), 2.28 (t, *J* = 7.1 Hz, 2H), 2.07 – 1.94 (m, 1H), 1.82 – 1.72 (m, 1H), 1.71 – 1.57 (m, 3H), 1.49 – 1.38 (m, 2H), 1.21 (d, *J* = 6.5 Hz, 3H). ^13^C NMR (101 MHz, D_2_O) δ 98.60­(Fuc-C1), 71.80, 69.59, 68.91, 68.77, 68.07, 66.81, 66.59, 63.08, 56.54, 50.02, 40.27, 38.91, 38.10, 35.48, 33.75, 27.87, 25.04, 15.31. HRMS: calcd *m*/*z* for C_47_H_89_N_4_O_20_S_2_ (M+H)^+^ 1094.56014; found 1094.5605.

### LA-EG_2_-α-d-mannopyranosyl-(1→2)-α-d-mannopyranoside (LA-EG_2_-DiMan)

Yield: 64.8 mg, 0.078 mmol, 52%; ^1^H NMR (400 MHz, D_2_O) δ 8.03 (s, 1H, triazole-H), 5.03 (s, 1H, DiMan-H1), 4.95 (s, 1H, DiMan-H1’), 4.63 (s, 2H), 4.58 (t, *J* = 5.0 Hz, 2H), 3.99 (s, 1H), 3.94 – 3.46 (m, 28H), 3.30 (t, *J* = 5.3 Hz, 2H), 3.12 (qt, *J* = 11.3, 6.4 Hz, 2H), 2.39 (dq, *J* = 12.3, 6.1 Hz, 1H), 2.16 (t, *J* = 7.2 Hz, 2H), 1.88 (dq, *J* = 13.5, 6.9 Hz, 1H), 1.64 (dq, *J* = 13.8, 7.3 Hz, 1H), 1.52 (hept, *J* = 7.7, 6.9 Hz, 3H), 1.31 (p, *J* = 7.6 Hz, 2H). ^13^C NMR (101 MHz, D_2_O) δ 176.77­(C = O), 170.93­(CCH), 125.47­(CCH), 102.30­(DiMan-C1’), 98.36­(DiMan-C1), 78.64, 73.25, 72.76, 70.30, 70.17, 69.94, 69.69, 69.54, 69.45, 69.01, 68.89, 68.81, 66.91, 66.87, 66.51, 63.13, 61.13, 60.89, 56.55, 50.07, 40.27, 38.92, 38.12, 35.47, 33.72, 27.84, 25.05. HRMS: calcd *m*/*z* for C_33_H_59_N_4_O_16_S_2_ (M+H)^+^ 831.3367; found 831.3367.

### LA-EG_6_-α-d-mannopyranosyl-(1→2)-α-d-mannopyranose (LA-EG_6_-DiMan)

Yield: 35.0 mg, 0.035 mmol, 59%; ^1^H NMR (400 MHz, D_2_O) δ 8.12 (s, 1H, triazole-H), 5.12 (s, 1H, DiMan-H1), 5.03 (s, Hz, 1H, DiMan-H1’), 4.72 (s, 2H), 4.67 (t, *J* = 5.0 Hz, 2H), 4.08 (dd, *J* = 3.4, 1.8 Hz, 1H), 4.04 – 3.96 (m, 3H), 3.96 – 3.60 (m, 41H), 3.40 (t, *J* = 5.3 Hz, 2H), 3.21 (ddt, *J* = 17.8, 11.0, 6.0 Hz, 2H), 2.50 (dq, *J* = 12.3, 6.0 Hz, 1H), 2.28 (t, *J* = 7.2 Hz, 2H), 1.99 (dq, *J* = 13.5, 6.9 Hz, 1H), 1.82 – 1.71 (m, 1H), 1.68 – 1.56 (m, 3H), 1.42 (p, *J* = 7.4 Hz, 2H). ^13^C NMR (101 MHz, D_2_O) δ 176.82­(C = O), 125.47­(CCH), 102.29­(DiMan-C1’), 98.35­(DiMan-C1), 78.63, 73.25, 72.75, 70.29, 70.16, 69.93, 69.67, 69.60, 69.55, 69.53, 69.44, 68.93, 68.89, 68.80, 66.90, 66.87, 66.50, 63.09, 61.13, 60.89, 56.52, 50.05, 40.27, 38.91, 38.09, 35.48, 33.74, 27.86, 25.04. HRMS: calcd *m*/*z* for C_41_H_76_N_4_O_20_S_2_ (M+Na)^+^ 1029.4236; found 1029.4234.

### LA-EG_12_-α-d-mannopyranosyl-(1→2)-α-d-mannopyranose (LA-EG_12_-DiMan)

Yield: 27.5 mg, 0.022 mmol, 56%; ^1^H NMR (400 MHz, D_2_O) δ 8.11 (s, 1H, triazole-H), 5.12 (s, 1H, DiMan-H1), 5.03 (s, 1H, DiMan-H1’), 4.71 (s, 2H), 4.66 (t, *J* = 5.0 Hz, 2H), 4.08 (s, 1H), 4.04 – 3.96 (m, 3H), 3.95 – 3.82 (m, 5H), 3.82 – 3.59 (m, 61H), 3.40 (t, *J* = 5.3 Hz, 2H), 3.31 – 3.13 (m, 2H), 2.50 (dq, *J* = 11.1, 5.5, 4.9 Hz, 1H), 2.32 – 2.23 (m, 3H), 2.00 (dq, *J* = 14.0, 7.0 Hz, 1H), 1.84 – 1.70 (m, 1H), 1.69 – 1.56 (m, 3H), 1.43 (p, *J* = 7.7 Hz, 2H). ^13^C NMR (101 MHz, D_2_O) δ 176.82­(C = O), 125.46­(CCH), 102.29­(DiMan-C1’), 98.35­(DiMan-C1), 78.63, 73.25, 72.75, 70.29, 70.15, 69.93, 69.67, 69.58, 69.43, 68.91, 68.79, 66.88, 66.49, 63.06, 61.13, 60.89, 56.53, 50.03, 40.27, 38.91, 38.09, 35.48, 33.75, 27.87, 25.04. HRMS: calcd *m*/*z* for C_53_H_102_N_4_O_26_S_2_ (M+NH_4_)^+^ 1288.2654; found 1288.6264.

### Gold Nanoparticle Preparation

#### G5-citrate

Trisodium citrate (97 mg, 0.33 mmol) was dissolved in 150 mL ultrapure water. The solution was then transferred to a freshly cleaned 250 mL three-necked flask and stirred vigorously. Aqueous solutions of tannic acid (0.1 mL, 2.5 mM) and K_2_CO_3_ (1 mL, 150 mM) were added and the reaction heated to 75 °C for 30 min. HAuCl_4_.3H_2_O (1 mL, 25 mM) was added and the reaction stirred for a further 30 min. The heating bath was then removed, and the solution was allowed to cool to room temperature naturally. The prepared GNP solution was transferred to a clean glass container and stored at RT.^39^

#### G5-glycans

100 mL of G5-Citrate stock was concentrated to ∼ 2 μM using 10 kDa MWCO spin column and washed with H_2_O (3 x) to remove any impurities. The concentration of the resulting G5 stock solution was determined from its absorbance at 515 nm using the Beer–Lambert law using G5′s molar extinction coefficient of 1.1 × 10^7^ M^–1^·cm^–1^.^37^ Then LA-EG_n_-glycan ligands dissolved in H_2_O to form 5 or 10 mM stocks were added to the G5 solution in a ligand: G5 molar ratio (LGMR) of 1000, 500, or 300. The resulting solution was mixed and stirred at RT for 2 days to complete the self-assembly process. The resulting mixture was passed through a 10 kDa MWCO spin column by centrifugation at 10,000 g for 6 min and the obtained residues were washed with H_2_O (3 × 200 μL) to give the G5-glycan stock. The concentration of the G5-glycan stock was determined from its absorbance at 515 nm using the Beer–Lambert law and a G5 molar extinction coefficient of 1.1 × 10^7^ M^–1^·cm^–1^.^37^

#### Determination of Glycan Valency

All the filtrate and washing-through liquids from the above G5-glycan preparations were collected, combined, freeze-dried, and redissolved in pure water to determine the amount of unbound ligand using the phenol-sulfuric acid method as described previously.^35–37^ 35 μL of each solution was mixed with 25 μL of 5% phenol and 175 μL of H_2_SO_4_. Samples were vortexed immediately then incubated at 90 °C for 20 min. The absorbance of the solution was recorded at 490 nm, and the dilution factors were then corrected to calculate the total amount of unconjugated glycan ligand against a standard calibration curve obtained with pure ligand. The difference in ligand amount between that added and that remained in the supernatant was conserved to have conjugated onto the G5 surface, allowing us to calculate the glycan valency for each G5-glycan conjugates.^36^

#### Protein Production and Labeling^36,66^

The soluble extracellular segment of DC-SIGN which faithfully replicated the tetrameric structure and glycan binding properties of full-length lectin, was expressed as inclusion body in *E. coli* and purified by mannose-Sepharose affinity column chromatography as reported earlier.^27,35,36,66^ The mutant protein, DC-SIGN Q-274C was constructed by site-directed mutagenesis and labeled with Atto-643 maleimide as described previously.^27,36^ The dye label site is close to, but not directly inside, the CRD glycan binding pocket, and hence does not affect DC-SIGN glycan binding after labeling as confirmed previously.^37,39^ The labeled proteins were purified by mannose-Sepharose affinity columns. The proteins were characterized by high-resolution mass spectroscopy (HRMS, SI Figures S8A, S9A) and DLS (SI, Figures S8B, S9B). The dye labeling efficiency (per protein monomer) was determined to be ∼ 82% for DC-SIGN based on the relative peak areas of the labeled and unlabeled protein peaks measured by HR-MS (see SI, Figure 9A).^27,39^

#### Solution Binding Studies^37–39^

All fluorescence spectra were recorded on a Cary Eclipse Fluorescence Spectrophotometer using a 0.70 mL quartz cuvette under a fixed excitation wavelength (λ_ex_) of 630 nm over a range of 650–800 nm. All measurements were performed in HEPES binding buffer (20 mM HEPES, 100 mM NaCl, 10 mM CaCl_2_, pH 7.8) containing 1 mg/mL of BSA. For the apparent *K*_d_ measurement, the concentrations of labeled DC-SIGN and G5-glycans were varied from 3 to 100 nM in a fixed protein: G5-glycan molar ratio (PGR) of 1:1. The samples were incubated at the desired temperature for 20 min before recording their fluorescence spectra. The fluorescence intensity of the protein in the absence of the G5-glycans, recorded under identical experiment conditions, were used to determine the quenching efficiency. The excitation and emission slit widths and instrument PMT voltages were adjusted to compensate the low fluorescence signals at low concentrations. The instrument setup was identical for DC-SIGN without and with equal molar of G5-glycan at the same concentration, ensuring that the change of instrument setup did not affect quenching efficiency (QE) determination.^38,39^ QEs for DC-SIGN binding to each G5-glycan were calculated at each concentration and the resulting QE-concentration relationship was fitted by Hill’s Equation to derive the apparent binding *K*_d_ values.

Thermodynamic measurements were obtained by repeating these measurements at three different temperatures of 22, 27, and 32 °C. The cuvette temperature was maintained by a water pump system. Buffer and sample temperatures were controlled by incubation in dry bath.^27,39^ The standard binding enthalpy and entropy changes were obtained from the van’t Hoff analysis of the linear fit of ln­(*K*_d_) against (1/T), where the standard binding enthalpy and entropy changes can be extracted from the slope and intercept of the linear fits.

#### Dynamic Light Scattering (DLS) and Zeta Potential Measurement^37^

All DLS measurements were performed on a Malvern Zetasizer NanoZS DLS system using a sample volume of 400 μL in 1 mL disposable polystyrene cuvettes. The hydrodynamic diameters (*D*_h_, all volume populations) of wild-type DC-SIGN, G5-EG_n_-glycans or G5-EG_n_-glycans + DC-SIGN samples were measured in a binding buffer (20 mM HEPES, 100 mM NaCl, 10 mM CaCl_2_, pH 7.8). Three to five consecutive scans were performed for each sample and the mean volume *D*_h_ distribution histograms for each sample were obtained by fitting the averaged percentage volume hydrodynamic sizes with Gaussian distributions. The resulting *D*_h_ values are reported as the mean ± 1/2 full width at half-maximum (fwhm). All protein containing samples were performed by mixing G5-glycans with DC-SIGN and incubating at RT for 20 min before taking the measurement. Zeta potential measurements were performed using a Malvern Zetasizer Nano. Disposable folded capillary cells (Malvern DTS1070) were rinsed with ultrapure water and dried under nitrogen. 100 nM GNPs in pure water were injected into the cells until all air bubbles were fully displaced. Zeta potential of each sample was measured 3 times, each containing 10 converged runs, and their average value was calculated with errors representing the standard deviations of the 3 measurements.

#### Viral Inhibition Studies

The effects of G5-EG_n_-glycans (LGMR 500) on Ebola virus glycoprotein (EBOV-GP) driven entry into 293T cells were assessed using our previously established procedures.^36–39^ Briefly, 293T cells seeded in 96-well plates were transfected with plasmids encoding DC-SIGN or DC-SIGNR or control transfected with empty plasmid (pcDNA). The cells were washed at 16 h post transfection and further cultivated at 37 °C, 5% CO_2_ in Dulbecco’s modified eagle medium (DMEM) containing 10% fetal bovine serum (FBS). At 48 h post transfection, the cells were exposed to twice the final concentration of G5-EG_n_-glycan inhibitors in DMEM supplemented with 10% FBS for 30 min in total volume of 50 μL. Thereafter, the resulting cells were inoculated with 50 μL of preparations of VSV vector particles encoding the luciferase gene and bearing either EBOV-GP (which can use DC-SIGN/R for augmentation of host cell entry) or the vesicular stomatitis virus glycoprotein (VSV-G, which cannot use DC-SIGN/R for augmentation of host cell entry). At 24 h post infection, luciferase activities in cell lysates were determined using a commercially available kit (PJK), following the manufacturer’s instructions and normalized by the corresponding control collected in the absence of the G5-glycans. The normalized concentration-dependent inhibition data were fitted by a normalized variable slope four-parameter logistic (4PL) curve model using Origin 2019b.

#### Data Analysis and Fitting

All fluorescence and DLS data were analyzed using Origin software (version 2019b). The fluorescence spectra of lectins alone and lectin + G5-glycan samples were integrated and used to calculate the QEs and presented as mean ± standard errors (SEs). The QE vs C plots were fitted by Hill’s equation. The DLS histograms were fitted by the standard Gaussian function to obtain the *D*_h_, and full-width at half-maximum (fwhm). The results obtained from the best fits were listed in the relevant tables with the standard fitting errors. The unprocessed luciferase activity data (indicative of viral entry efficiency) of samples after treatment with varying doses of G5-EG_n_-glycans were compared with their respective control sample in the absence of G5-EG_n_-glycan inhibitors.

## Supplementary Material



## Data Availability

All data associated with this paper are contained within the manuscript and the Supporting Information. For the purpose of open access, the authors have applied a Creative Commons Attribution (CC BY) license to any Author Accepted Manuscript arising from this submission.

## Notes

Materials, instruments, and methods showing the synthesis and characterization of the LA-EG_n_-glycans by ^1^H and ^13^C NMR spectra; determination of glycan valency; surface footprint calculation; production, characterization, and labeling of DC-SIGN by HR-MS and DLS; UV–vis and DLS histograms of citrate-stabilized G5; zeta potentials of G5-citrate before and after cap exchange with LA-EG_2_-DiMan/Fuc ligands at LGMRs of 1000, 500, and 300; DLS histograms of G5-EG_n_-glycans and G5-EG_n_-glycan plus varying ratios of DC-SIGN; plots of QE versus *C* for G5-glycans binding with DC-SIGN fitted with Hill’s equation; van’t Hoff plots with linear fits of the ln­(*K*_d_)–(1/*T*) relationships for G5-glycans (LGMRs of 500 and 300) binding with DC-SIGN; and unprocessed virus inhibition data and cell cytotoxicity data showing the relevant cellular luciferase activities after treatment with varying concentrations of the G5-EG_n_-glycan inhibitors (PDF)

## Abbreviations

MLGI: multivalent lectin glycan interaction
CRD: carbohydrate recognition domain
GNP: gold nanoparticle
NSET: nanosurface energy transfer
DC-SIGN: dendritic cell-specific intercellular adhesion molecule-3-grabbing nonintegrin
DC-SIGNR: DC-SIGN-related endothelial cell surface lectin
fwhm: full width at half-maximum
BSA: bovine serum albumin
DLS: dynamic light scattering
QE: quenching efficiency
VSV: vesicular stomatis virus
