# Supplementary material for: Probing Glycan-Gold Nanoparticle Architectures: Glycan Type, Density, and Linker Length, Governing Multivalent Lectin Binding and Viral Inhibition
Source: ACS Appl Mater Interfaces. 2026 Apr 6;18(15):21543–61. doi: 10.1021/acsami.5c25677 (PMC13107371; doi:10.1021/acsami.5c25677)
Supplement: Supplementary file 1 [file am5c25677_si_001.pdf]

Supporting Information (SI)

Probing Glycan-Gold Nanoparticle Architectures: Glycan Type, Density and Linker Length, Governing Multivalent Lectin Binding and Viral Inhibition

Maisie Holbrow-Wilshaw [1,2], Darshita Budhadev [2] Amy Madeleine Kempf [3], Inga Nehlmeier [3], Erin Tait [4], Stefan Pöhlmann [3], W. Bruce Turnbull [2], Yuan Guo [5,*], Dennis McGonagle,[1] Dejian Zhou [2,*]

[1] Leeds Institute of Rheumatic and Musculoskeletal Medicine, School of Medicine, University of Leeds, Leeds LS2 9JT, United Kingdom.

[2] School of Chemistry and Astbury Centre for Structural Molecular Biology, University of Leeds, Leeds LS2 9JT, United Kingdom.

[3] Infection Biology Unit, German Primate Center–Leibniz Institute for Primate Research, 37077 Göttingen, and Faculty of Biology and Psychology, University of Göttingen, 37073 Göttingen, Germany.

[4] School of Biomedical Sciences and Astbury Centre for Structural Molecular Biology, University of Leeds, Leeds LS2 9JT, United Kingdom.

[5] School of Food Science and Nutrition, and Astbury Centre for Structural Molecular Biology, University of Leeds, Leeds LS2 9JT, United Kingdom.

Contents

1. Materials, instruments and methods S2
2. Synthesis of LA-EG_n-glycan ligands S3-S13
3. G5-glycan production and characterization S14-S18
4. Determination of G5 surface glycan valency S19-S21
5. Protein production and characterization S22-S23
6. Determination of D_h-PGR relationship for G5-EG_n-glycan-DC-SIGN S24-S34
7. QE vs C plots and data tables for G5-glycans binding to DC-SIGN S35-S37
8. Van't Hoff plots for G5-EG_n-glycans binding to DC-SIGN thermodynamics S38-S39
9. Unprocessed viral inhibition and cytotoxicity data S40-S43
10. Supporting references S44

1. Materials, instruments and methods

D-mannose was purchased from Carbosynth, L-fucose, triphenyl phosphine, sodium azide, tetra-n-butylammonium iodide (TBAI), N,N'-Dicyclohexylcarbodiimide (DCC), Tris[(1-benzyl-1H-1,2,3-triazol-4-yl)methyl]amine (TBTA), Gold(III) chloride trihydrate and phenol were purchased from Alfar Aesar. Carbon tetrabromide, acetic anhydride, pyridine, sodium methoxide, sodium ascorbate, guanidine HCl, triton-X 100, Silica 60A, copper sulfate, dimethylformamide, chloroform d4, and tri sodium citrate were purchased from Sigma Aldrich. DL-Lipioc acid, triethylamine, and FITC were purchased from Acros Organics. 4-Dimethyl-aminopyridine (DMAP) and 2-[2-(2-Chloroethoxy)-ethoxy]ethanol were purchased from Fluorochem. Sodium citrate, Sodium hydroxide, HEPES, calcium chloride, sodium chloride, EDTA, HCl, H_2SO4, BSA, methanol, DCM, THF, petroleum ether, hexane, acetonitrile, ethanol, amberlight® IR-120 (H^+), Tween20, Tris base and HNO_3 were purchased from Fisher Scientific. β-mercaptoethanol was purchased from Bio-Rad. BSA was purchased form BioServ UK. Maleimide-Atto-643 was purchased from ATTO-TEC. PEG linkers (H_2N-EG_{12}-C≡CH, H_2N-EG_6-C≡CH, H_2N-EG_2-C≡CH) were purchased from PurePEG. Dry solvents were obtained using an Innovative Technology Inc. PureSolv® solvent purification system. Ultrapure water (resistance >18.2 MΩ cm) was purified by an ELGA Purelab classic UVF system and was used for all experiments and making buffers.

All moisture-sensitive reactions were performed under nitrogen atmosphere using oven-dried glassware. Evaporations were performed under reduced pressure on a Buchi Rotavapor R-300. Concentration or washing was performed in Amicon ultra-S2 centrifugal filter tubes or Sartorius Stedim Lab vivaspin 500 with a molecular weight cut off (MWCO) of 10,000 or 30,000. Dialysis was performed using Thermofischer Scientific 14,000 MWCO BioDesign Dialysis Tubing. Flash column chromatography (FCC) was carried out by pre-absorption of the crude material onto silica gel 60A (Merk) or dissolving in the minimum volume of an appropriate eluent and loading on top. Solvents for FCC and TLC are listed in volume:volume percentages. Centrifugation was performed using either a Hettich Universal 320/320R, a Thermo Scientific Heraeus Fresco 17 or a Beckman Coulter Avanti JXN-30 centrifuge, depending on the speed and volume, at room temperature (r.t.) unless otherwise stated. Lyophilisation was performed using a Virtis Benchtop K freeze dryer.

^{1}H NMR spectra were measured at 25° C on a 500 or 400 MHz Bruker spectrometer using D_2O, Methanol-d4 or $CDCl_3$ with the residual solvent as the internal standard at room temperature. Chemical shifts are given in parts per million relative to tetramethylsilane (TMS) and the coupling constants (*J*) are given in Hertz. High resolution mass spectra (HR-MS) were obtained on a Bruker Impact QqTOF II mass spectrometer and m/z values are reported in Daltons to four decimal places. Liquid chromatography mass spectroscopy (LC-MS) was used to analyse all other samples and was performed using a Bruker AmaZon speed mass spectrometer and are quoted to two decimal places. UV-vis absorption spectra were recorded on a Varian Cary 50 bio UV-Visible Spectrophotometer over 200-800 nm using 1 mL quartz cuvette with an optical path length of 1 cm or on a Nanodrop 2000 spectrophotometer (Thermo scientific) over the range of 200-800nm using 1 drop of the solution with an optical path length of 1 mm. The hydrodynamic diameters were recorded on a Malvern ZETASizer-Nano using disposable polystyrene cuvettes. All fluorescence spectra were recorded on a Cary Eclipse Fluorescence Spectrophotometer using a 0.70 mL quartz cuvette. All numerical data were analysed and plotted using Origin 2019b.

2. Synthesis of LA-EG_n-glycan ligands

The α-D-mannopyranosyl-(1→2)-α-D-mannopyranose appending a flexible di(ethylene glycol) azide linker (N_3-EG_2-DiMan), and a lipoamide-di(ethylene glycol)-propargyl (LA-EG_2-C≡CH) were synthesized as per reported in the literature.[1, 2] The LA-EG_n-glycan ligands were synthesized by copper-catalyzed azide alkyne click interactions between LA-EG_n-C≡CH and N_3-EG_2-dimannose or N_3-EG_2-fucose as reported in the Experimental Section. All the glycans and linkers were synthesized in-house and purified using our established protocols.[2-4]

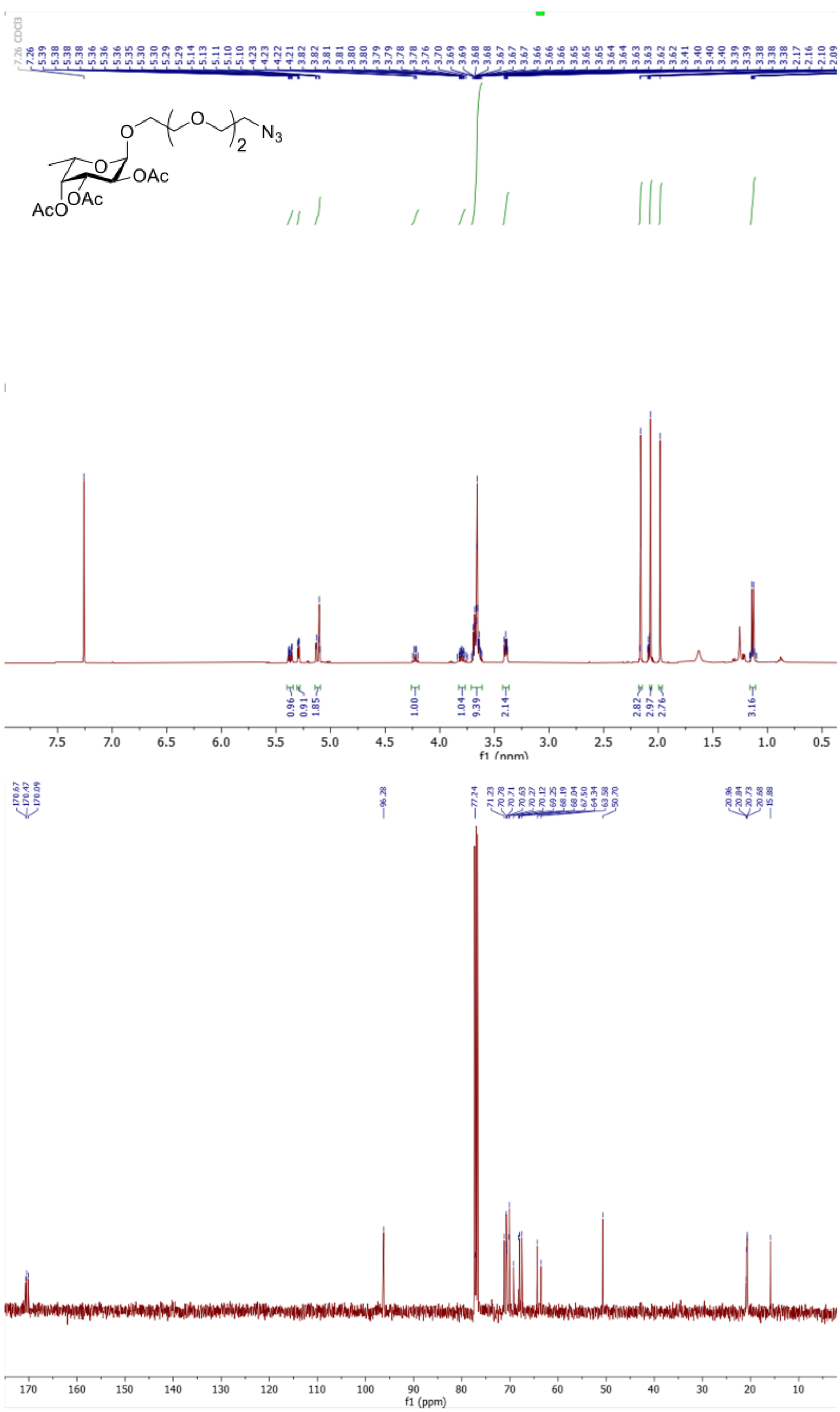

The ^{1}H- (top) and ^{13}C- (bottom) NMR spectra of 8-Azido-3,6-dioxaoctyl-2,3,4-tri-*O*-acetyl-α-L-fucopyranoside in $CDCl_3$

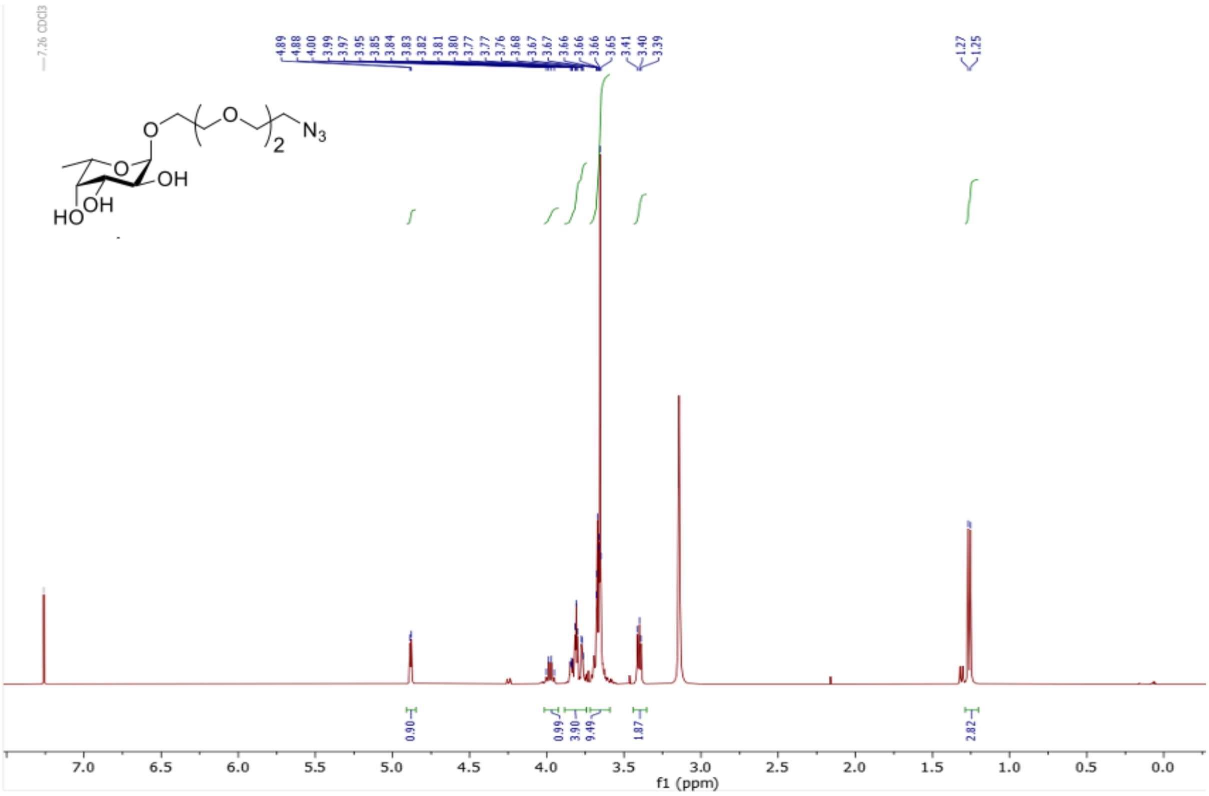

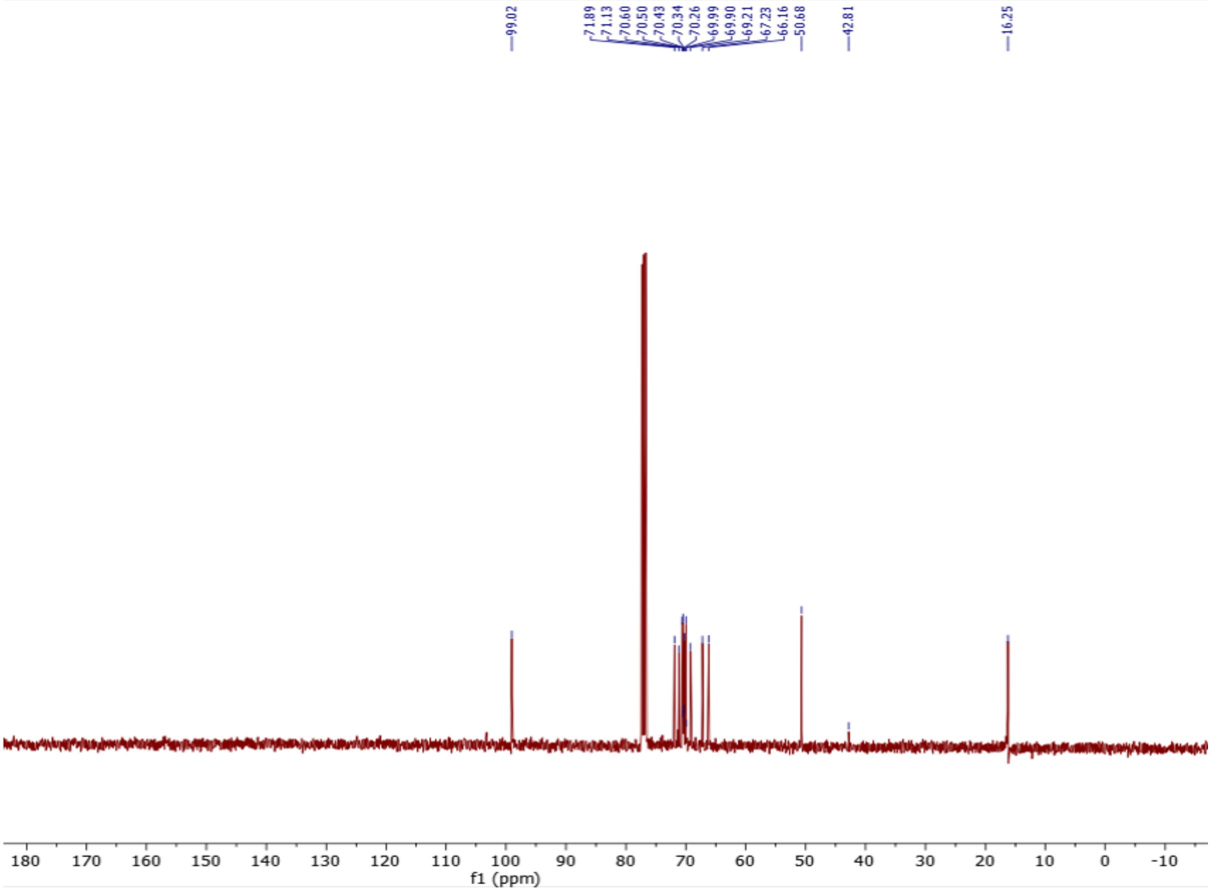

The ^{1}H- (top) and ^{13}C- (bottom) NMR spectra of 8 8-Azido-3,6-dioxaoctyl-*O*-α-L-fucopyranoside in $CDCl_3$

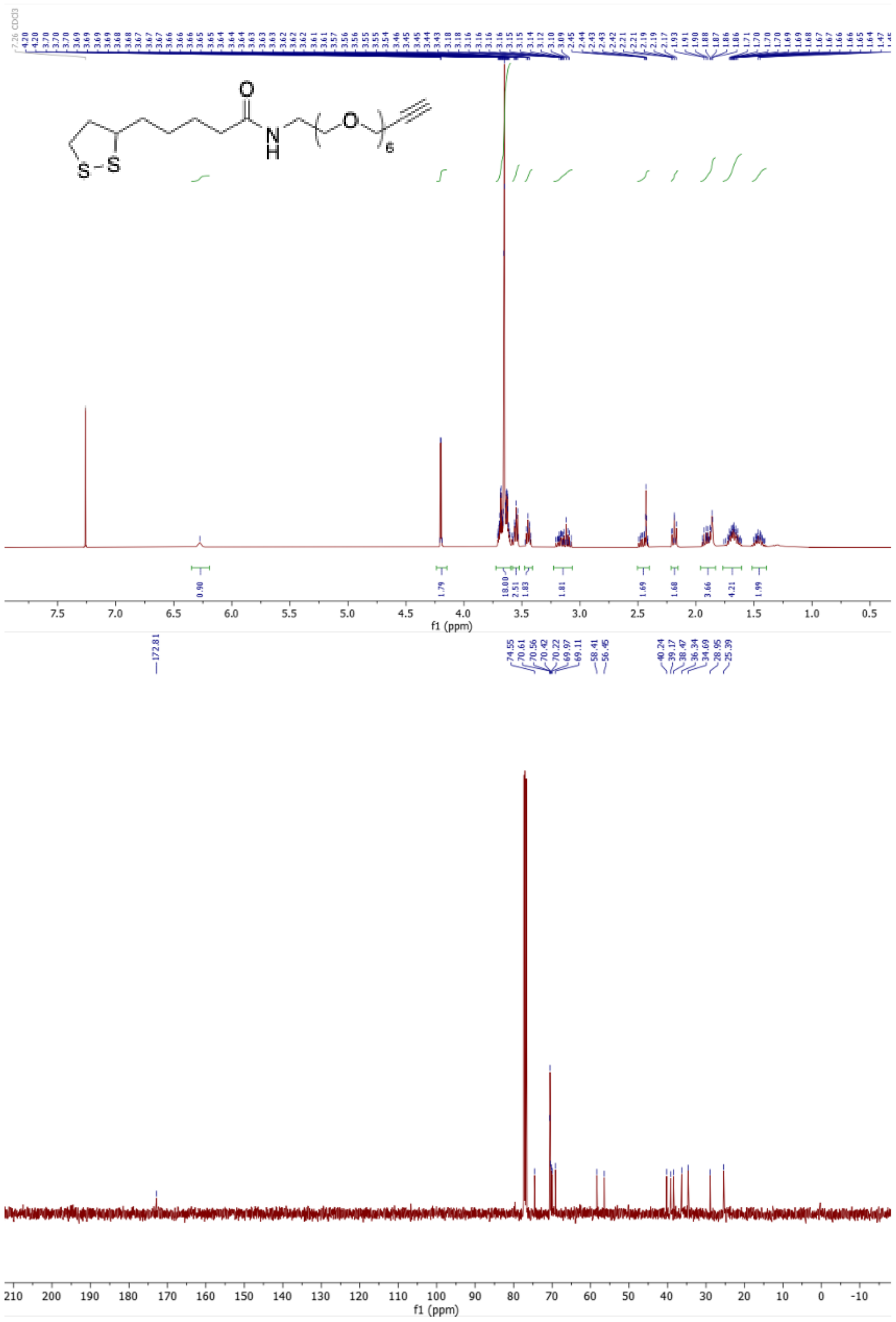

The ^{1}H- (top) and ^{13}C- (bottom) NMR spectra of LA-EG$_6$-C≡CH in $CDCl_3$.

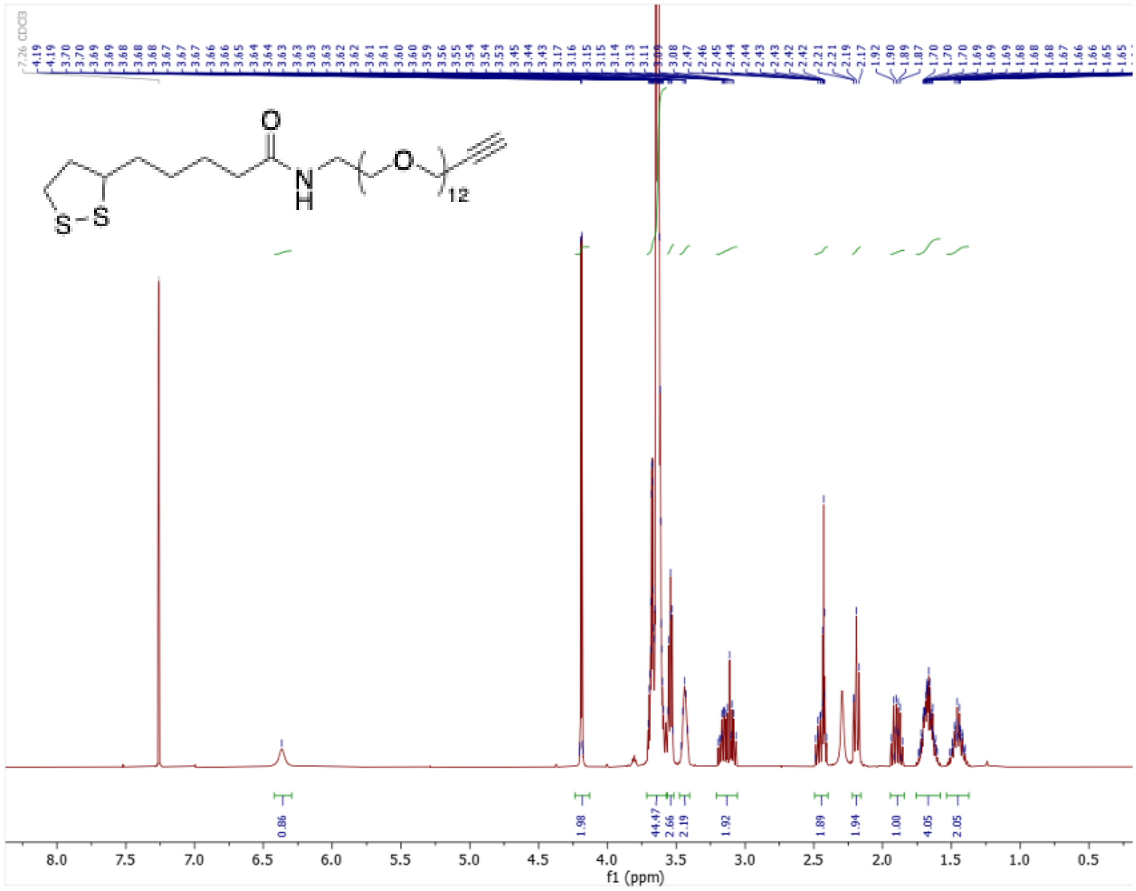

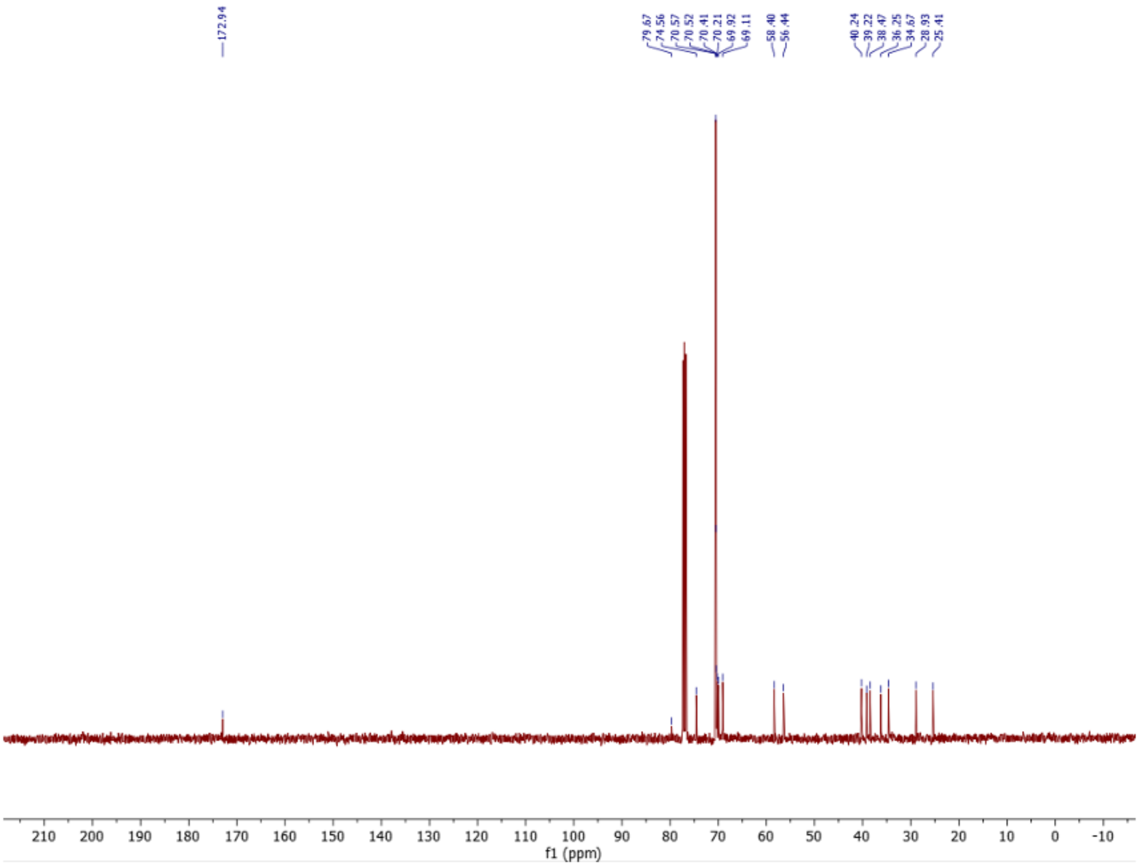

The ^{1}H- (top) and ^{13}C- (bottom) NMR spectra of LA-EG$_{12}$-C≡CH in $CDCl_3$.

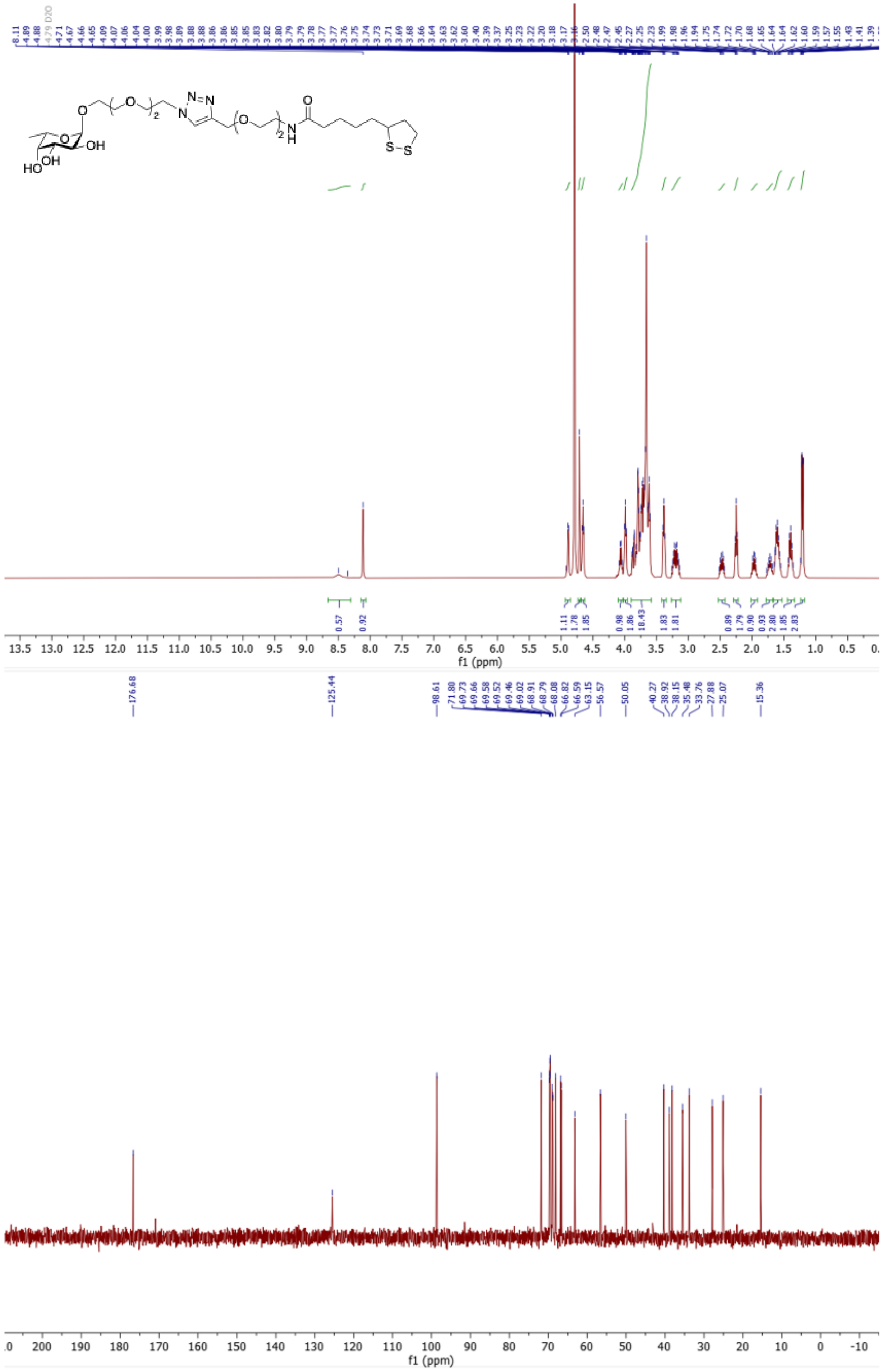

The ^{1}H- (top) and ^{13}C- (bottom) NMR spectra of LA-EG2-fucose in D_2O.

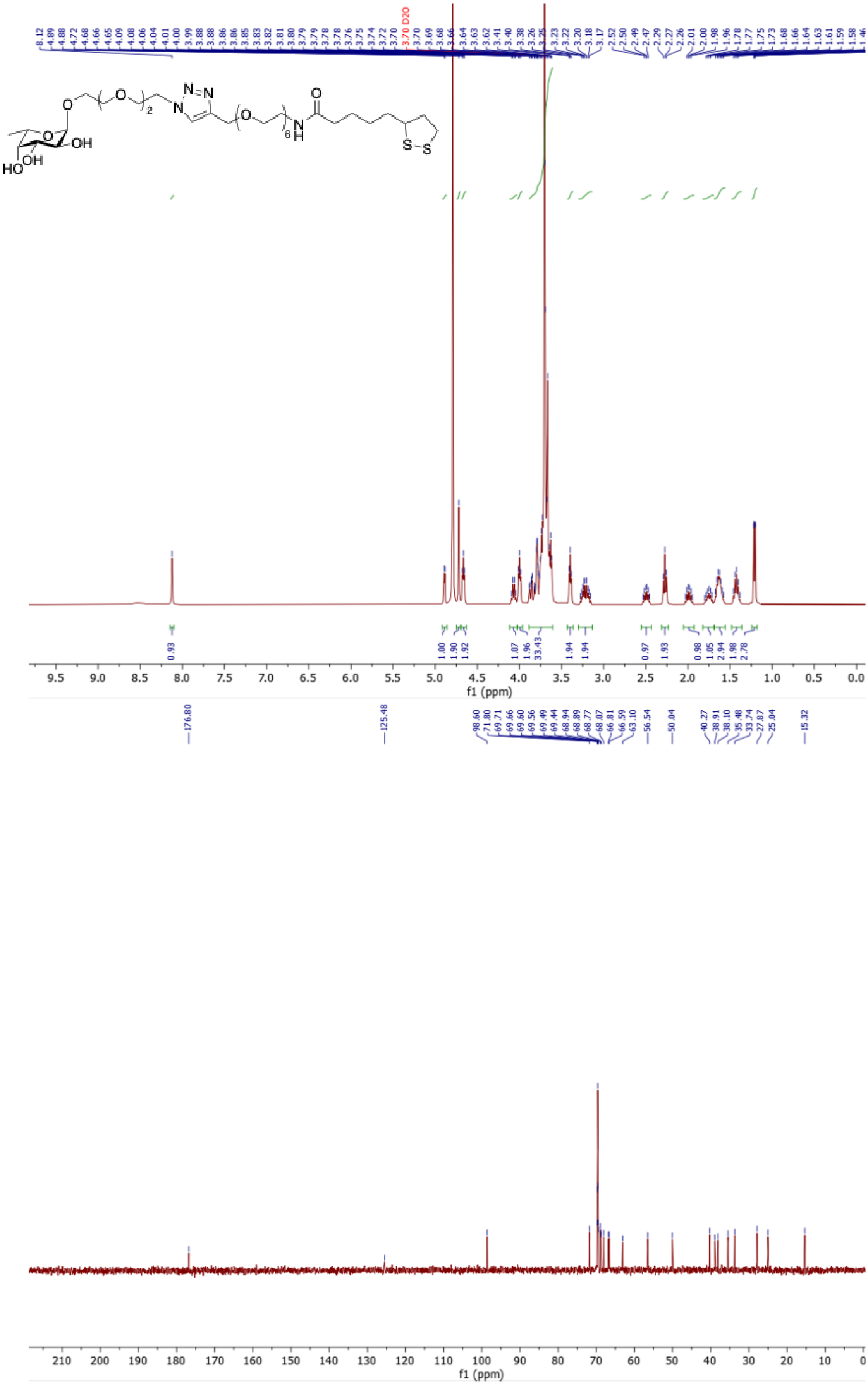

The ^{1}H- (top) and ^{13}C- (bottom) NMR spectra of LA-EG$_6$-fucose in D_2O.

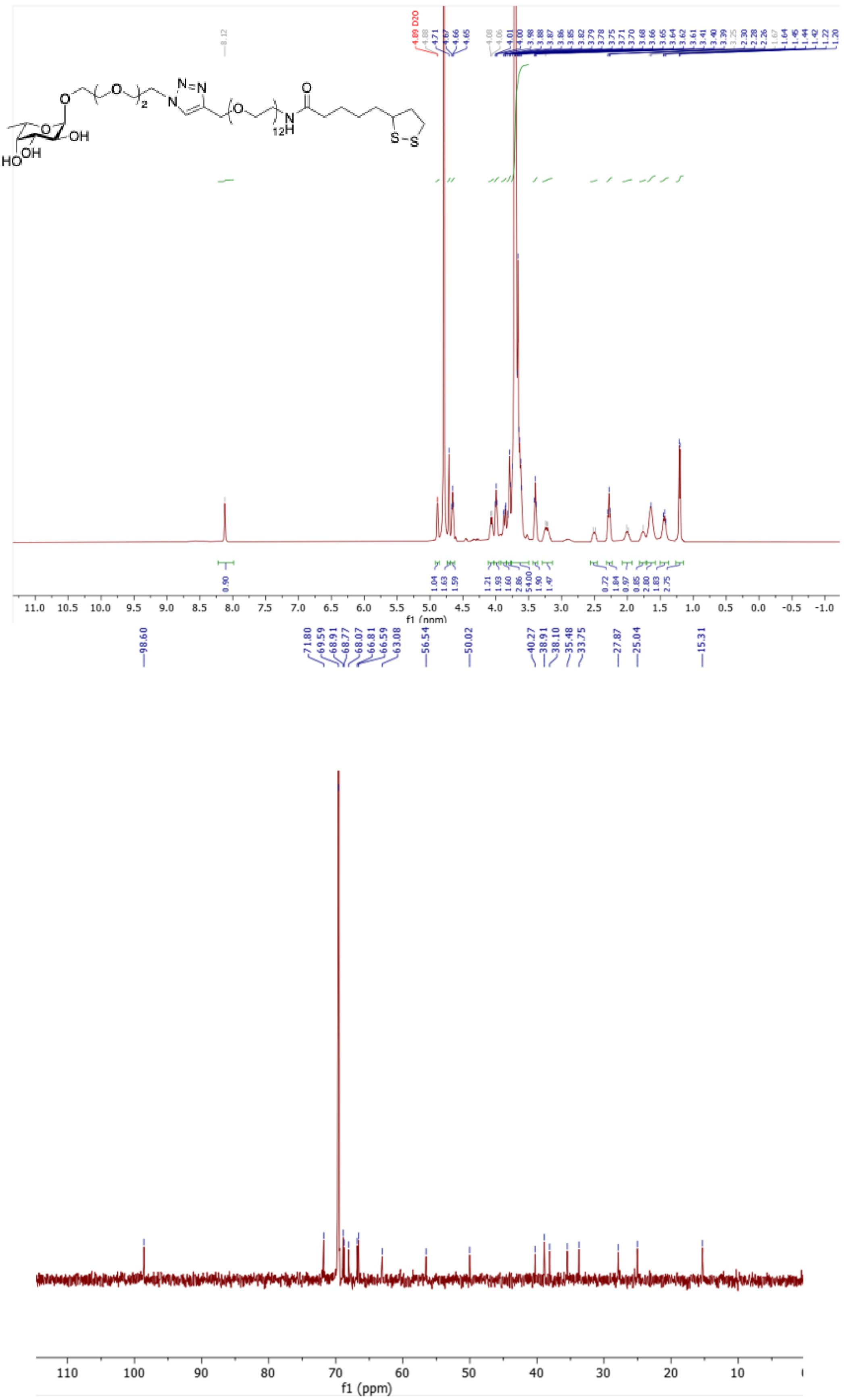

The ^{1}H- (top) and ^{13}C- (bottom) NMR spectra of LA-EG12-fucose in D_2O.

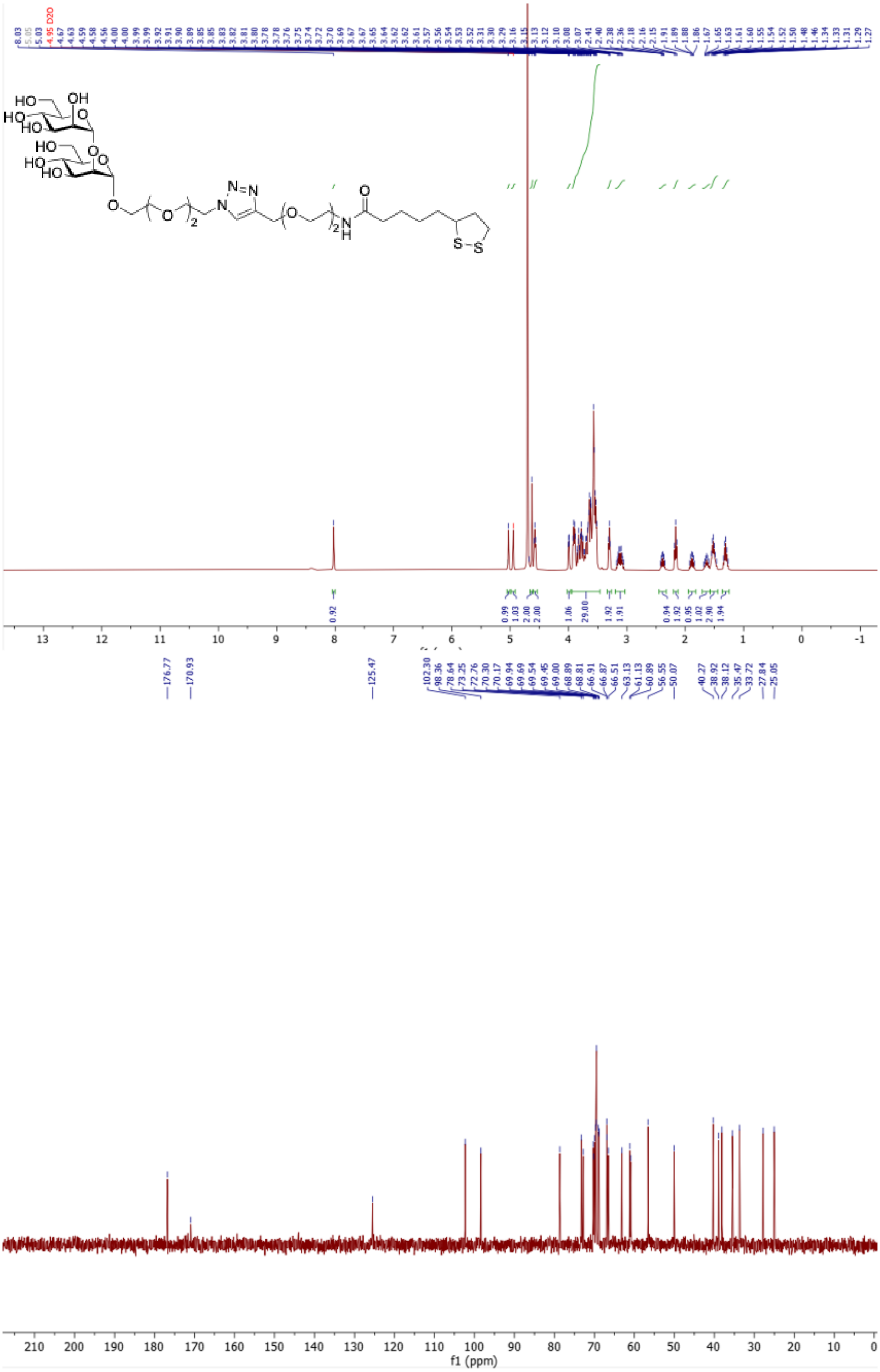

The ^{1}H- (top) and ^{13}C- (bottom) NMR spectra of LA-EG2-DiMan in D_2O.

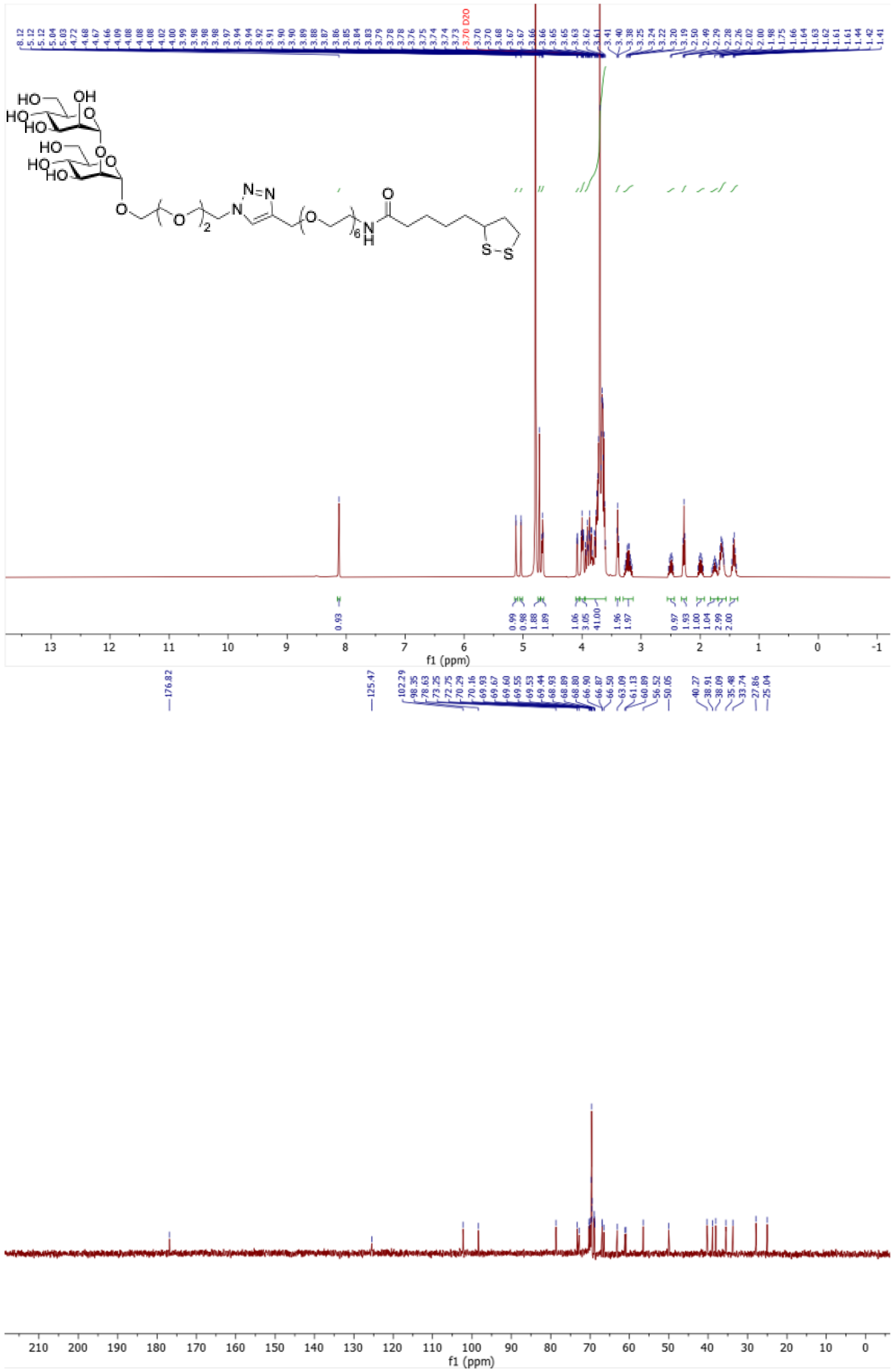

The ^{1}H- (top) and ^{13}C- (bottom) NMR spectra of LA-EG$_6$-DiMan in D_2O.

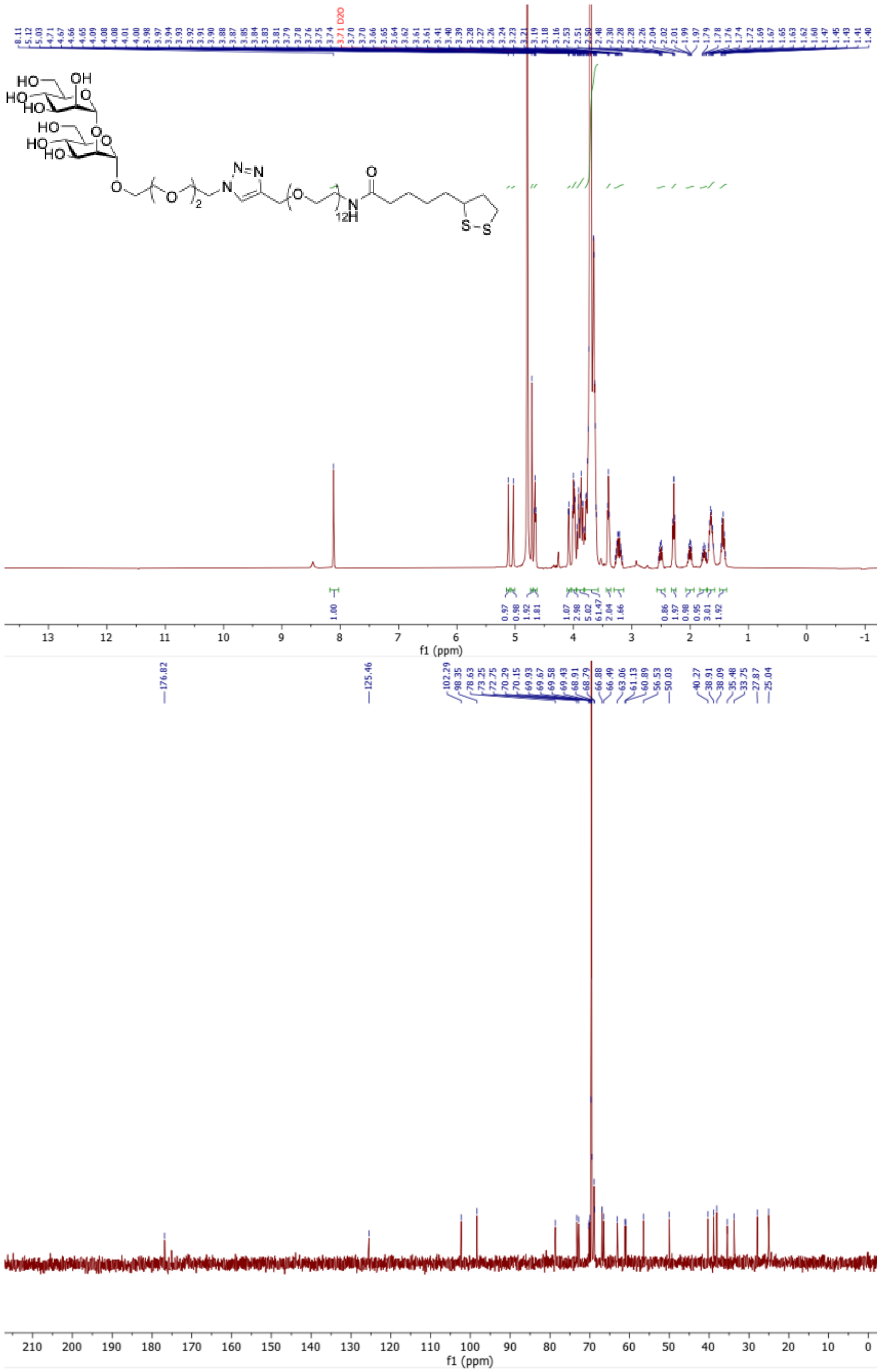

The ^{1}H- (top) and ^{13}C- (bottom) NMR spectra of LA-EG$_{12}$-DiMan in D_2O.

3. G5-glycan production and characterisation.[2, 4, 5]

Gold nanoparticles with average diameters of ~5 nm (abbreviated as G5) were synthesised in-house via our established protocols.[6] Details of the synthesis and purification procedures were given in the Experimental Section. The hydrodynamic diameter (D_h) histogram of the G5-citrate stock is given in Figure S1.

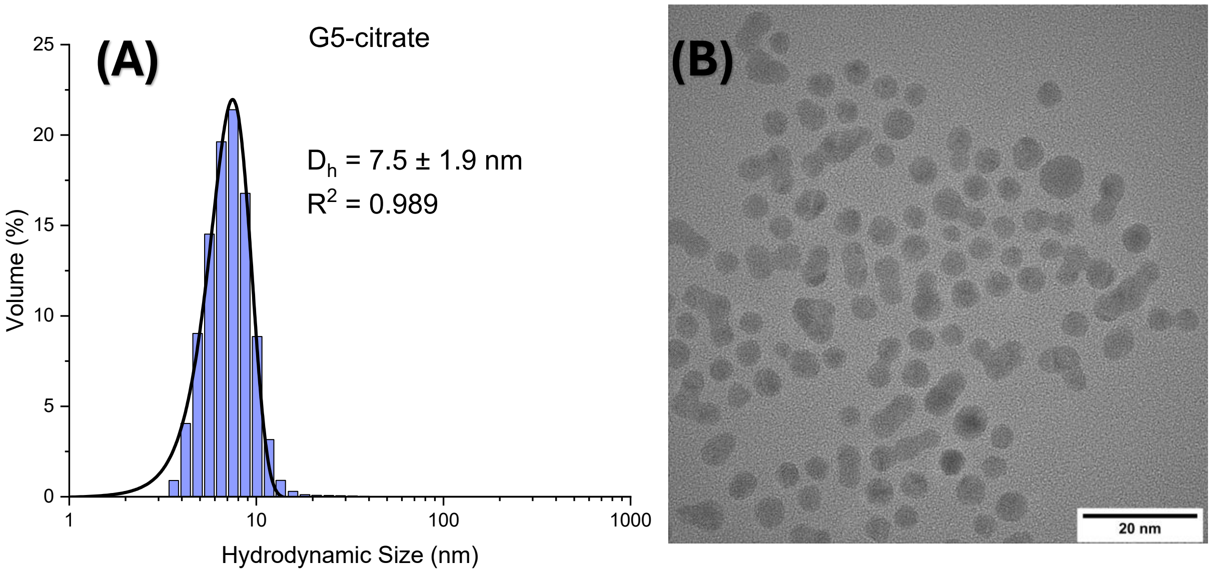

Figure S1. (A) Volume population hydrodynamic size distribution histogram of G5-citrate, fitted with a Gaussian distribution curve. (D_h value given as mean ± ½ FWHM). (**B**) A TEM image of the prepared G5-citrate, which shows that most particles are roughly spherical with a diameter of ~ 5 nm.

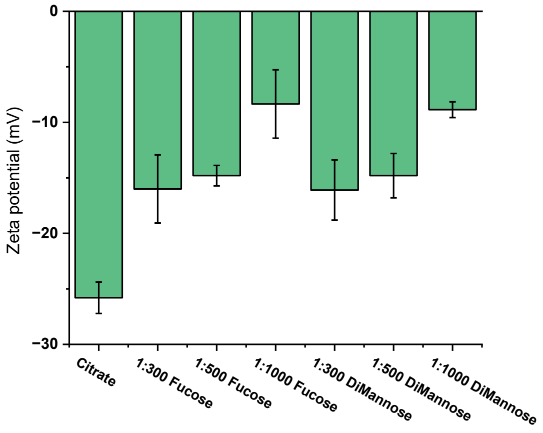

Figure S2. Zeta potentials of G5-citrate before (labeled as citrate) and after cap-exchange with the LA-EG$_2$-Fuc (labeled as fucose) and LA-EG$_2$-DiMan (labeled as DiMannose) ligands at varying G5: ligand molar ratios as indicated in graph. The negative zeta potential of G5-citrate (~-25.5 mV) is reduced significantly and progressively with the increasing ligand: G5 molar ratio (LGMR), suggesting that the negatively charged citrate ions on G5-citriate surfaces are increasingly displaced by neutral LA-EG$_2$-glycan ligands, especially at LGMR of 1000. The zeta potentials of G5-EG$_2$-DiMan and G5-EG$_2$-Fuc are effectively the same at each LGMR, suggesting the two ligands have the same ability to self-assemble on G5 surface and displace the original negatively charged citrate ions.

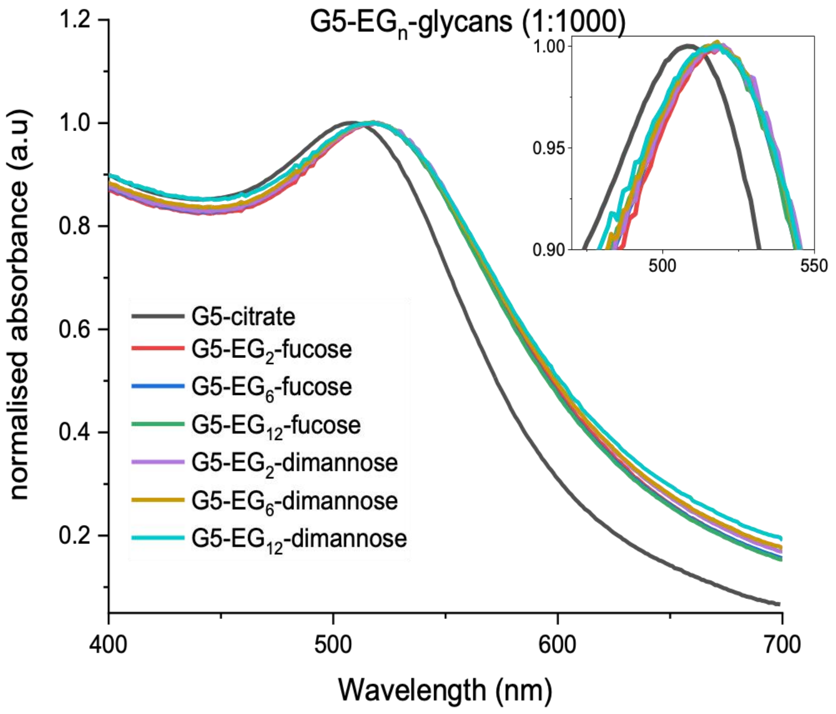

Figure S3. Normalised UV-Vis absorption spectra for G5-citrate before (dark grey) and after cap exchange with LA-EG$_n$-glycan ligands at an LGMR of 1000.

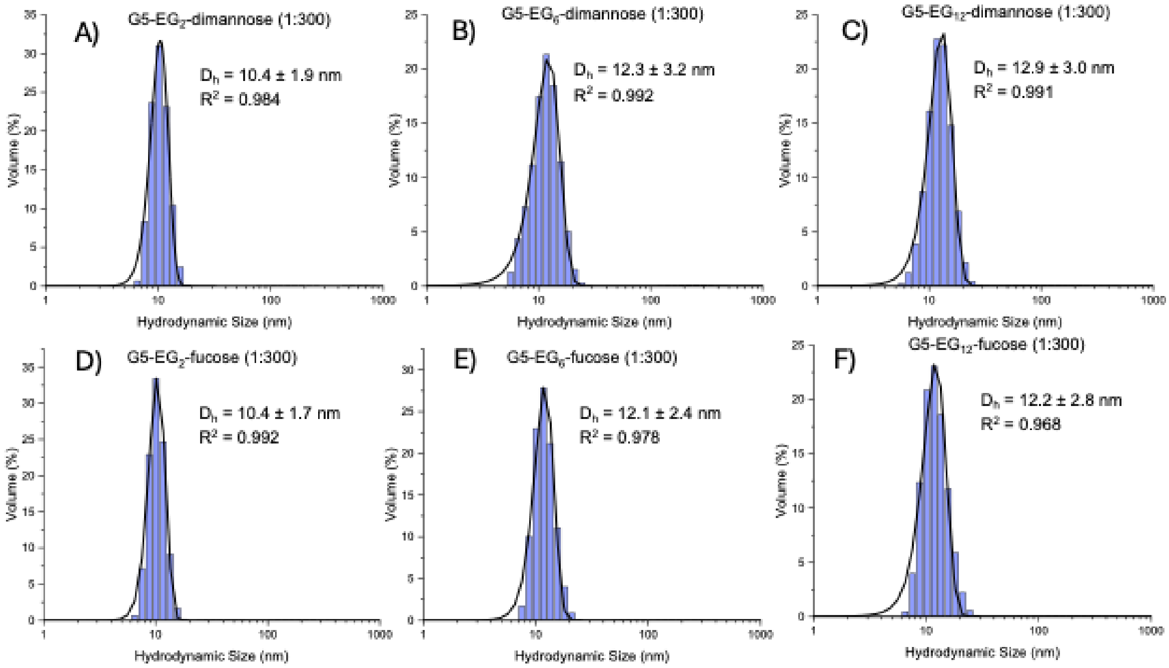

Figure S4. Volume population hydrodynamic size distribution histograms in a binding buffer fitted with a Gaussian distribution curve for A) G5-EG_2-dimannose, B) G5-EG_2-fucose, C) G5-EG_6-dimannose, D) G5-EG_6-fucose, E) G5-EG_{12}-dimannose, F) G5-EG_{12}-fucose, made in a ligand: G5 molar ratio (LGMR) of 300 (D_h values given as mean ± ½ FWHM).

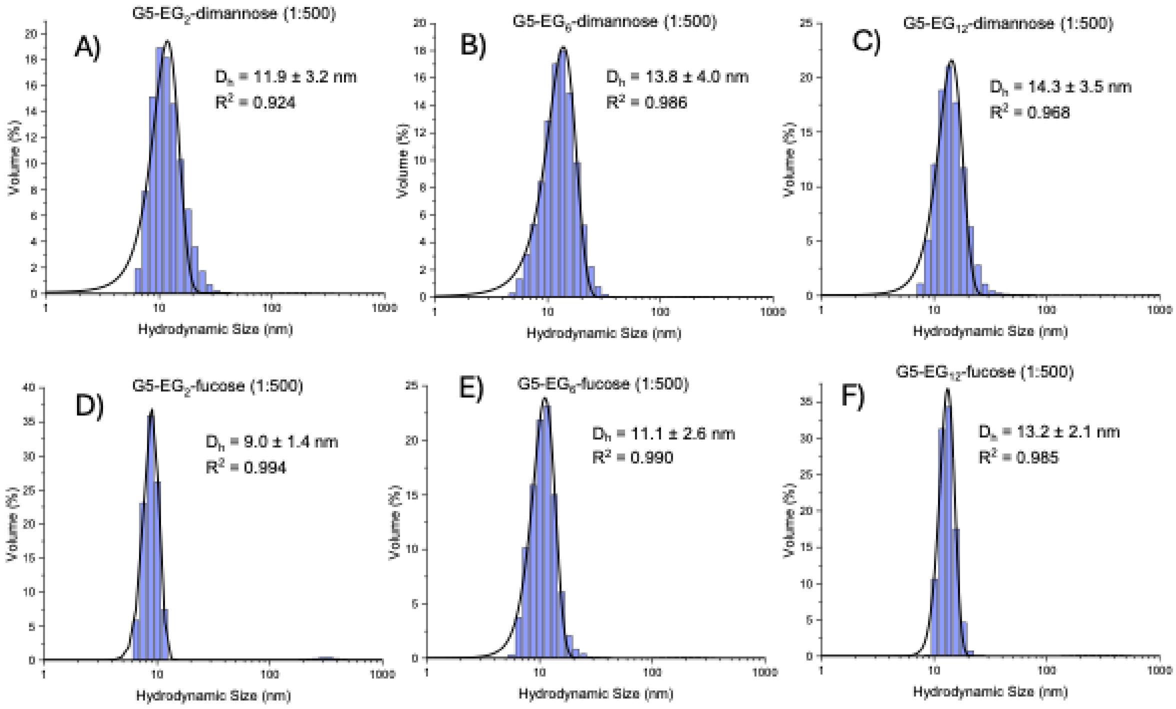

Figure S5. Volume population hydrodynamic size distribution histograms in a binding buffer fitted with a Gaussian distribution curve for A) G5-EG_2-dimannose, B) G5-EG_2-fucose, C) G5-EG_6-dimannose, D) G5-EG_6-fucose, E) G5-EG_{12}-dimannose, F) G5-EG_{12}-fucose, made in a ligand: G5 molar ratio (LGMR) of 500 (D_h values given as mean ± ½ FWHM).

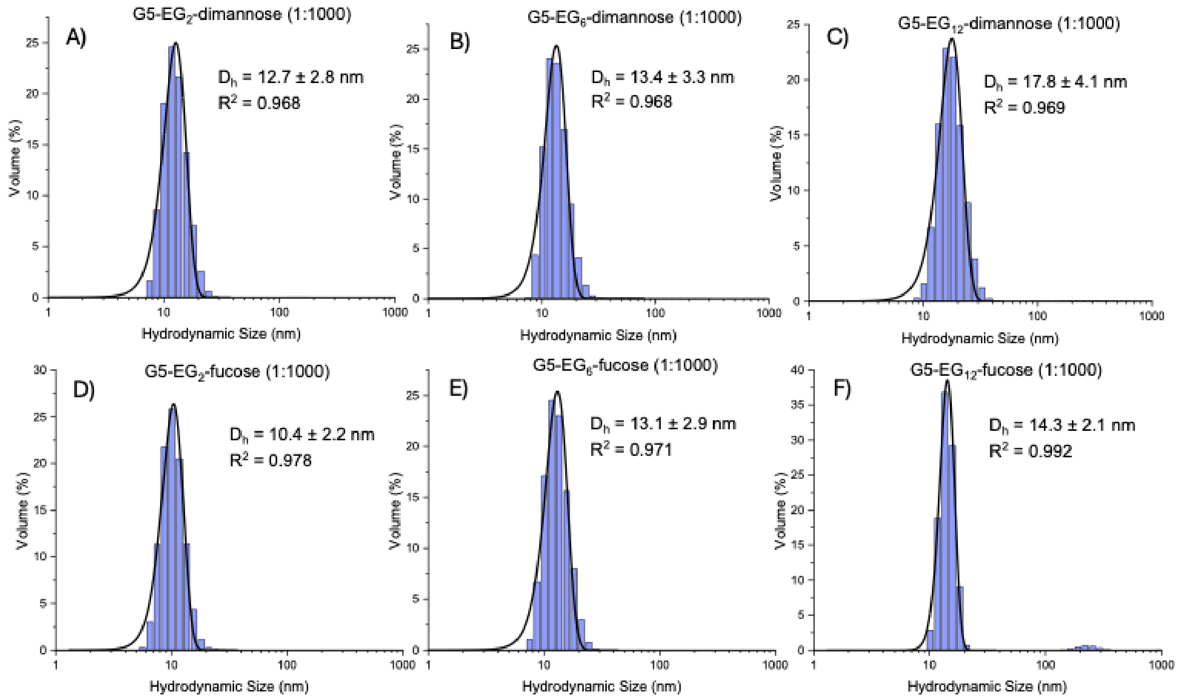

Figure S6. Volume population hydrodynamic size distribution histograms in a binding buffer fitted with a Gaussian distribution curve for A) G5-EG2-dimannose, B) G5-EG2-fucose, C) G5-EG6-dimannose, D) G5-EG6-fucose, E) G5-EG12-dimannose, F) G5-EG12-fucose, made in a ligand: G5 molar ratio (LGMR) of 1000 (D_h values given as mean ± ½ FWHM).

Table S1. Summary of the hydrodynamic diamters (D_hs) of G5-EGn-glycans prepared in three ligand: G5 molar ratios (LGMRs) of 1000, 500 and 300 in binding buffer. D_h values given as mean ± ½ FWHM.

	LGMR = 1000	***LGMR = 500***	***LGMR = 300***
G5-EGn-glycan	*D_h (nm)*	*D_h (nm)*	*D_h (nm)*
G5-EG2-DiMan	12.7 ± 2.8	11.9 ± 3.2	10.4 ± 1.9
G5-EG6-DiMan	13.4 ± 3.3	13.8 ± 4.0	12.3 ± 3.2
G5-EG12-DiMan	17.8 ± 4.1	14.3 ± 3.5	12.9 ± 3.0
G5-EG2-Fuc	10.4 ± 2.2	9.8 ± 2.7	10.4 ± 2.2
G5-EG6-Fuc	13.1 ± 2.9	11.1 ± 2.6	12.1 ± 1.7
G5-EG12-Fuc	14.3 ± 2.5	13.2 ± 2.1	12.2 ± 2.8

4. Determination of glycan valency on G5-glycans[2, 3]

The number of glycan ligands conjugated to each G5 was quantified using a phenol-sulfuric acid method of carbohydrate quantification.[7] Calibration curves were constructed by adding sulphuric acid (175 µL) and phenol (35 µL, 5% w/w H_2O) to a known amount of LA-EG_n-glycan dissolved in 35 µL H_2O. Samples were vortexed immediately then incubated at 90 °C for 20 minutes and their absorbance at 490 nm read on a Varian Cary 50 Bio UV-Visible Spectrophotometer in a disposable cuvette. A calibration curve of absorbance at 490 nm vs. ligand amount for each LA-EG_n-glycan was constructed which was fitted with a linear fit (Figure S8).

After cap exchange, G5-glycans were washed 3 x with dH_2O in a 10 kDa MWCO Amicon ultra-S2centrifugal filter. The flow through was combined, lyophilised and dissolved in H_2O. 35 µL of these samples were treated as described above for the calibration samples. The calibration curve was then used to determine the quantity of unbound LA-EG_n-glycan from their absorbance at 490 nm. The number of bound ligand could then be calculated by subtracting the unbound ligand was the total used initially. By comparing this to the mols of G5 used in the initial cap exchange, the quantity of glycan per nanoparticle was calculated. The average number of LA-glycan ligands conjugated to each G5 were given in Table S2 below.

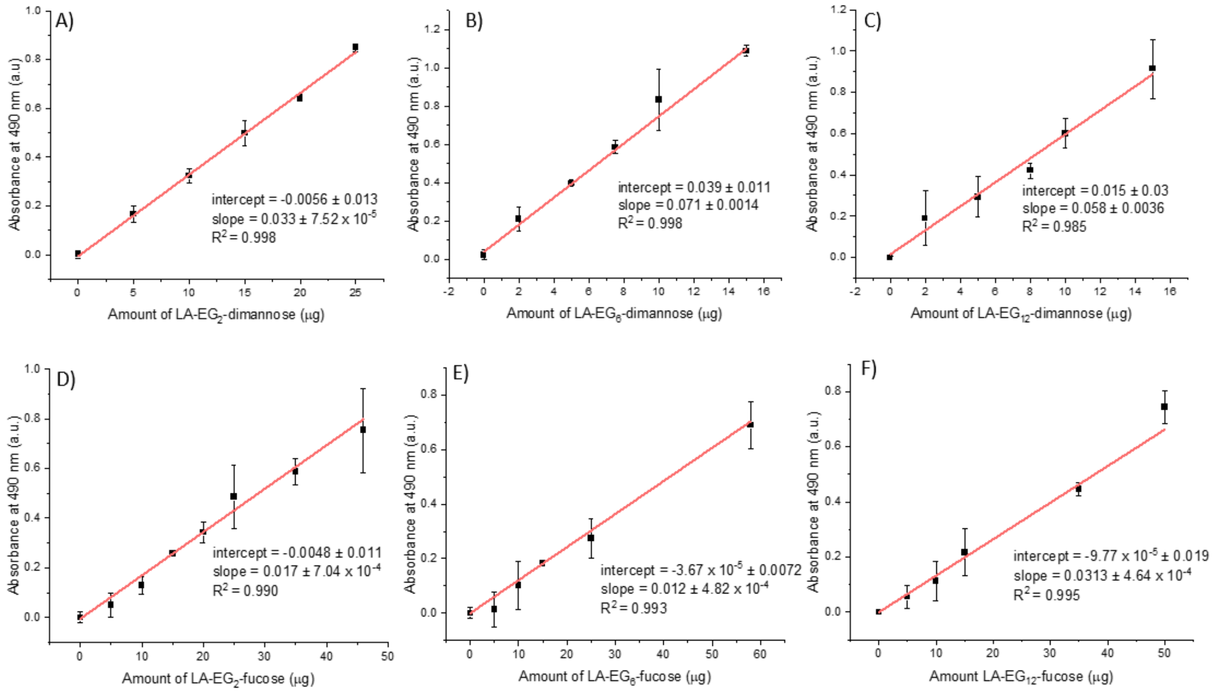

Figure S7. Plot of absorbance at 490 nm against amount of A) LA-EG_2-DiMan, B) LA-EG_6- DiMan, C) LA-EG_{12}-DiMan, D) LA-EG_2-fucose, E) LA-EG_6-fucose, F) LA-EG_{12}-fucose fitted by the linear relationship; y = mx + c.

Table S2. Summary of the glycan valency (N) and footprint of G5-EG_n-glycans prepared in three LGMRs (1000, 500 and 300). Valencies are given as an average of two repeat measurements with the error representing the standard deviations. Glycan footprint = π D_h^2/N, where D_h and N are the hydrodynamic diameter and glycan valency of each G5-EG_n-glycan, respectively.

	LGMR = 1000		*LGMR = 500*		*LGMR = 300*	
G5-EG$_n$-glycan	***Valency (N)***	**Footprint (nm^2)**	***Valency (N)***	**Footprint (nm^2)**	***Valency (N)***	**Footprint (nm^2)**
G5-EG_2-DiMan	659 ± 145	0.77 ± 0.17	355 ± 19	1.25 ± 0.07	164 ± 3	2.07 ± 0.04
G5-EG_6-DiMan	661 ± 22	0.85 ± 0.03	360 ± 15	1.66 ± 0.07	171 ± 16	2.78 ± 0.26
G5-EG_{12}-DiMan	622 ± 37	1.60 ± 0.10	379 ± 18	1.69 ± 0.08	181 ± 13	2.89 ± 0.21
G5-EG_2-Fuc	599 ± 6	0.57 ± 0.01	333 ± 23	0.91 ± 0.06	157 ± 45	2.16 ± 0.62
G5-EG_6-Fuc	519 ± 96	1.04 ± 0.19	380 ± 8	1.02 ± 0.02	174 ± 17	2.64 ± 0.26
G5-EG_{12}-Fuc	556 ± 45	1.15 ± 0.09	370 ± 13	1.48 ± 0.05	184 ± 9	2.54 ± 0.12

4.1. Calculation of the average inter-glycan distance on G5

The average inter-glycan distance (X) of each G5-EG_n-glycan (n = 2, 6 or 12) was calculated from their D_h and glycan valency, based on the method reported by Hill et al.[8] For a G5-glycan with a hydrodynamic radius of r (r = 1/2 D_h) covered with N ligand, the footprint of each ligand (k) on the G5-glycan surface is:

$$k = \frac{4\pi r^2}{N}$$

The average deflection angle of each ligand on the G5 surface (θ, in degrees) was calculated via the equation below.

$$\theta = \frac{360\sqrt{\frac{k}{\pi}}}{r\pi} = \frac{229.3}{\sqrt{N}}$$

Using these data, the average inter-glycan distance on G5-EG_n-glycans (X) was then calculated via the following equation:

$$X = 2r\sin\frac{\theta}{2}$$

Table S3. Summary of the deflection angle (θ) and inter-glycan distance (X) of G5-EG_n-glycans prepared under three different ligand: G5 molar ratios (LGMRs) of 1000, 500 and 300.

	LGMR = 1000		***LGMR = 500***		***LGMR = 300***	
G5-EG_n-glycan	**θ (°)**	**X (nm)**	**θ (°)**	**X (nm)**	**θ (°)**	**X (nm)**
G5-EG_2-DiMan	8.9 ± 1.0	0.99 ± 0.25	12.2 ± 0.3	1.26 ± 0.34	17.9 ± 0.2	1.62 ± 0.30
G5-EG_6-DiMan	8.9 ± 0.2	1.04 ± 0.26	12.1 ± 0.2	1.45 ± 0.42	17.5 ± 0.8	1.87 ± 0.50
G5-EG_{12}-DiMan	9.2 ± 0.3	1.42 ± 0.33	11.8 ± 0.3	1.46 ± 0.36	17.0 ± 0.6	1.91 ± 0.45
G5-EG_2-fucose	9.4 ± 0.05	0.85 ± 0.18	12.6 ± 0.4	1.07 ± 0.30	18.3 ± 2.8	1.65 ± 0.43
G5-EG_6-fucose	10.1 ± 1.0	1.15 ± 0.28	11.8 ± 0.1	1.13 ± 0.27	17.4 ± 0.9	1.83 ± 0.27
G5-EG_{12}-fucose	9.7 ± 0.4	1.21 ± 0.22	11.9 ± 0.2	1.37 ± 0.22	16.9 ± 0.4	1.79 ± 0.41

5. Protein production and characterisation[3, 9]

DC-SIGN was expressed from *E. coli* and purified by sepharose-mannose affinity chromatography as described previously.[3, 9] The concentration was determined the absorbance at 280 nm using a monomer extinction coefficient of 70400 $M^{-1}cm^{-1}$. The protein was characterised by high resolution mass spectrometry (HR-MS, Figure SA) and DLS (Figure SB). The calculated molecular weight of DC-SIGN based on its amino acid sequence is 39197.22.

HR-MS calculated: 39197.22, found: 39201.53.
DLS D_h: 14.0 ± 2.0 nm

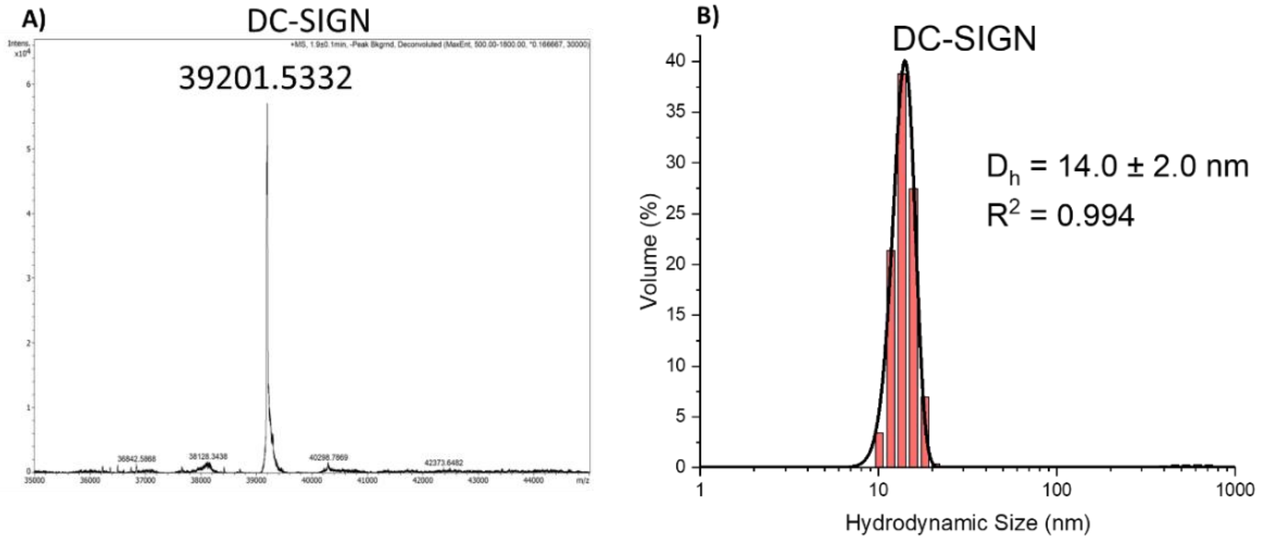

Figure S8. A) Deconvolved HRMS spectra of the monomeric extracellular domain of DC-SIGN (wild type; WT, DC020), B) Volume population hydrodynamic size distribution histogram fitted with lognormal Gaussian distribution for the extracellular domain of DC-SIGN (WT, DC020).

The production and purification of site-specific Atto-643 labelled lectin was performed on a mutant lectin with a site-specific cysteine mutation at Q274C in DC-SIGN as described previously.[3, 9] The protein was characterised by high resolution mass spectrometry (HR-MS, Figure SA) and DLS (Figure SB). The successful Atto-643 labelling was confirmed from an increase of molecular weight of ~882 Da, corresponding to the MW of Atto-643 dye, as observed in the HR-MS spectrum (Figure SA). The labelling efficiency was calculated from the abundance of the labelled peak area to that of the total lectins (labelled + unlabelled), giving ~82% per monomer for DC-SIGN (Figure SA).

DC020Q274C HR-MS calculated [M + 2Ca^{2+}]: 39252.38, found: 39254.47.
D020Q274C-atto634: HR-MS calculated [M + Ca^{2+}]: 40140.30, found: 40136.36.
D_h: 14.4 ± 2.6 nm
Labelling Efficiency: 82% (per monomer)

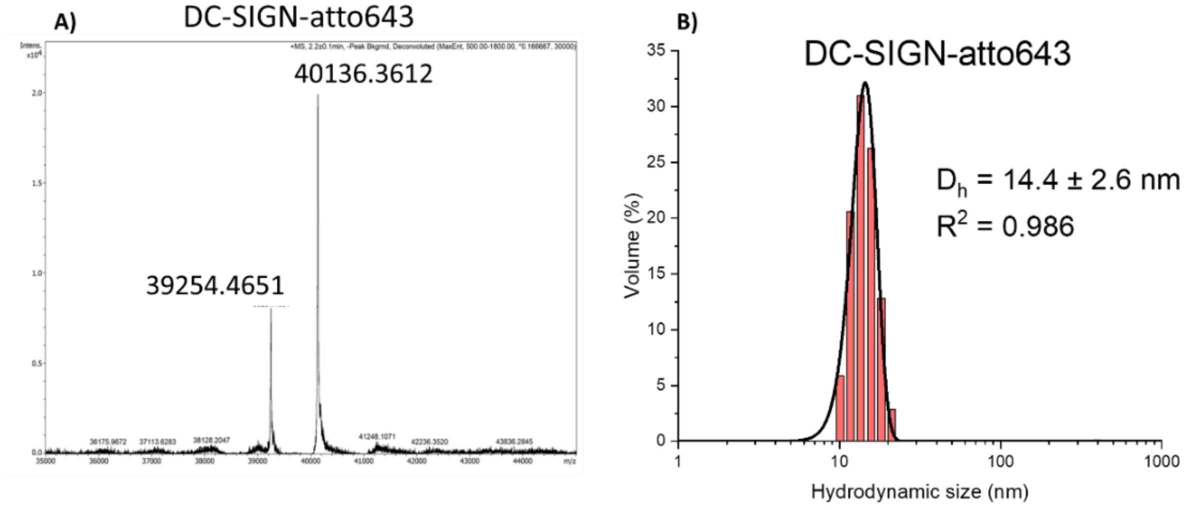

Figure S9. A) Deconvolved HRMS spectra of the monomeric extracellular domain of DC-SIGN-atto643 (DC020Q274C-atto643), B) Volume population hydrodynamic size distribution histogram fitted with lognormal Gaussian distribution for the extracellular domain of DC-SIGN-atto643 (DC020Q274C-atto643)

6. Determination of D_h-PGR relationship for G5-EG$_n$-glycan-DC-SIGN

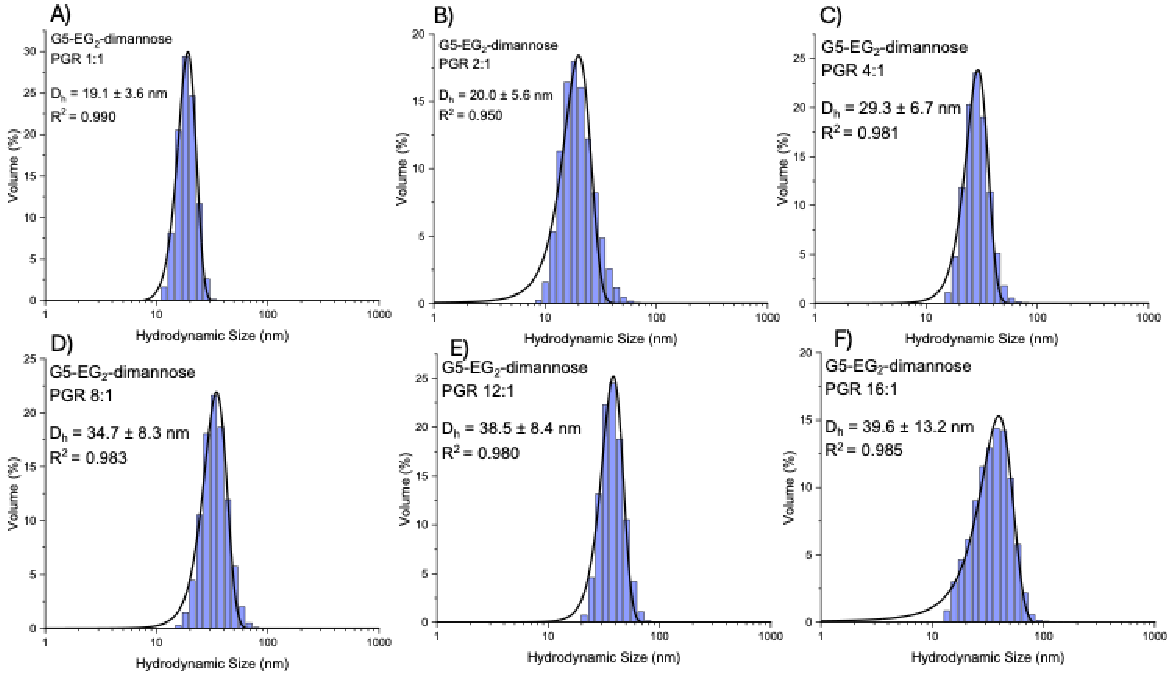

Figure S10. Volume population hydrodynamic size distribution histograms fitted with Gaussian fits for the binding of G5-EG$_2$-DiMan (LGMR 1000, 20 nM) with DC-SIGN at varying protein: G5 molar ratios (PGRs) in binding buffer of (A) 1:1, (B) 2:1, (C) 4:1, (D) 8:1, (E) 12:1, and (F) 16:1 (D_h values given as mean ± ½ FWHM).

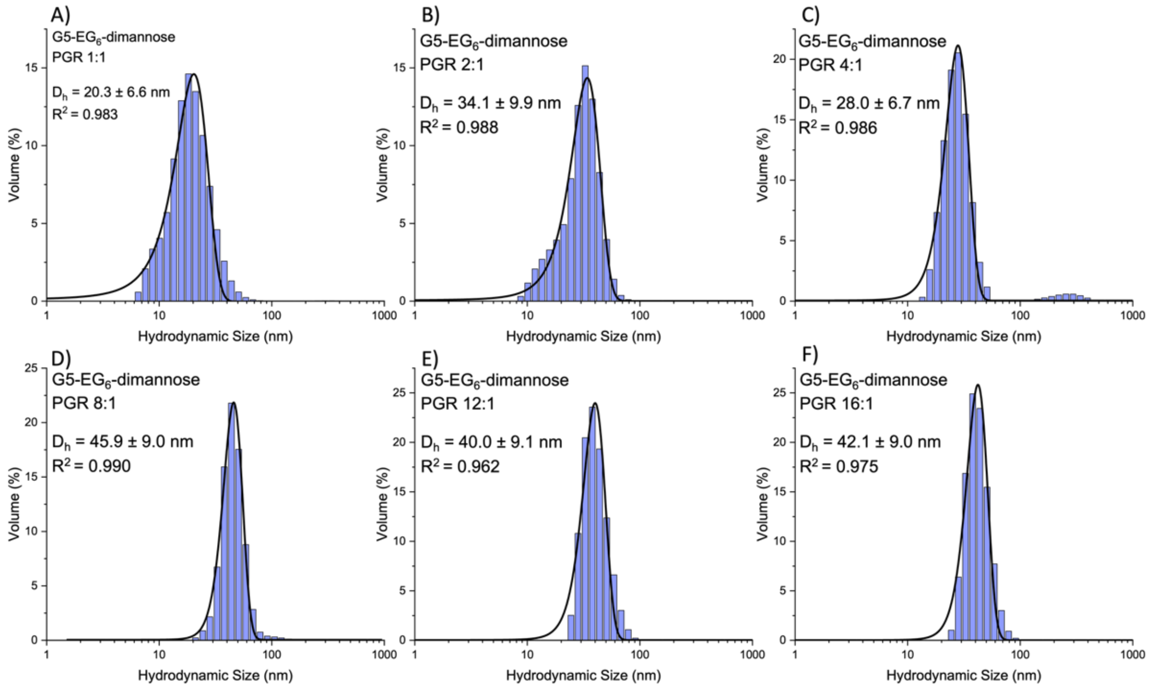

Figure S11. Volume population hydrodynamic size distribution histograms fitted with Gaussian fits for the binding of G5-EG$_6$-DiMan (LGMR 1000, 20 nM) with DC-SIGN at varying protein: G5 molar ratios (PGR) in binding buffer of (A) 1:1, (B) 2:1, (C) 4:1, (D) 8:1, (E) 12:1, and (F) 16:1 (D_h values given as mean ± ½ FWHM).

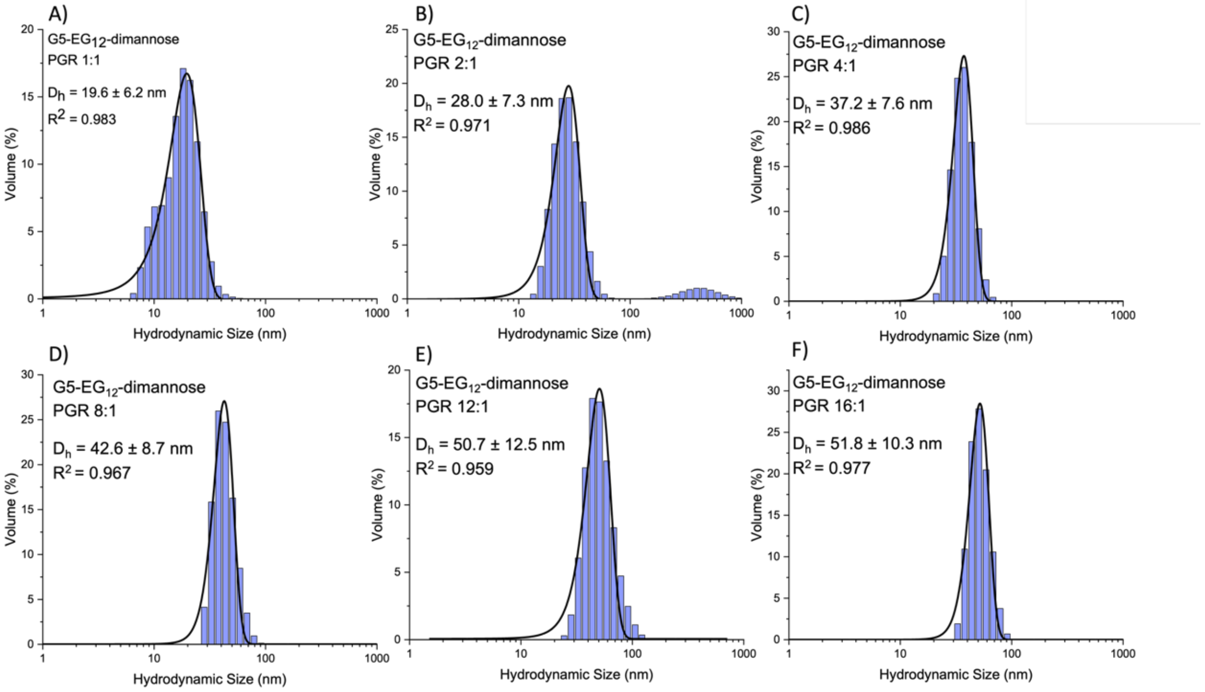

Figure S12. Volume population hydrodynamic size distribution histograms fitted with Gaussian fits for the binding of G5-EG_{12}-DiMan (LGMR 1000, 20 nM) with DC-SIGN at varying protein: G5 molar ratios (PGR) in binding buffer of (A) 1:1, (B) 2:1, (C) 4:1, (D) 8:1, (E) 12:1, and (F) 16:1 (D_h values given as mean ± ½ FWHM).

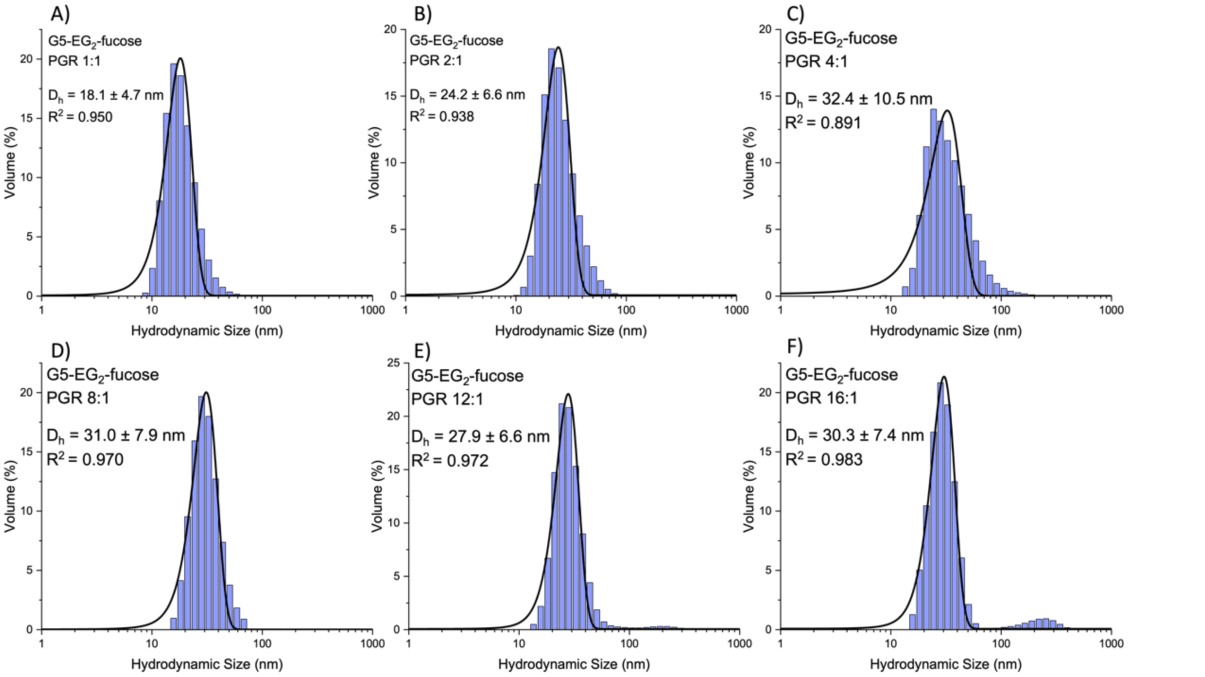

Figure S13. Volume population hydrodynamic size distribution histograms fitted with Gaussian fits for the binding of G5-EG_2-fucose (LGMR 1000, 20 nM) with DC-SIGN at varying protein: G5 molar ratios (PGR) in binding buffer of (A) 1:1, (B) 2:1, (C) 4:1, (D) 8:1, (E) 12:1, and (F) 16:1 (D_h values given as mean ± ½ FWHM).

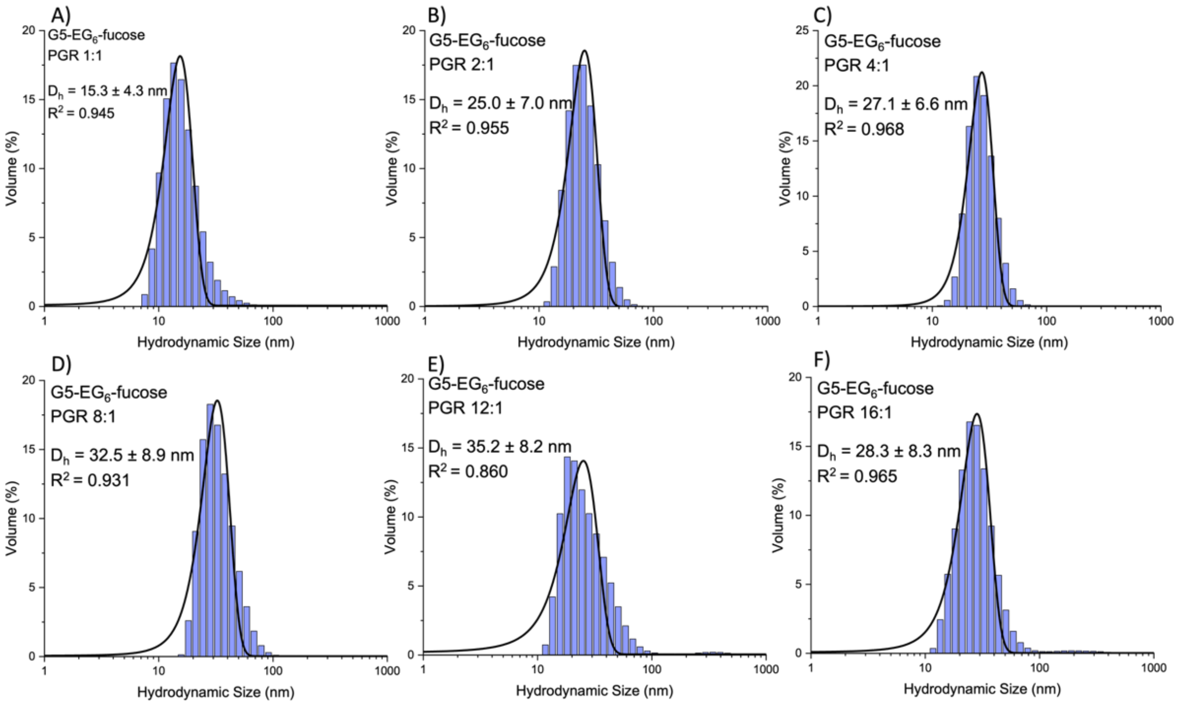

Figure S14. Volume population hydrodynamic size distribution histograms fitted with Gaussian fits for the binding of G5-EG_6-fucose (LGMR 1000, 20 nM) with DC-SIGN at varying protein:G5 molar ratios (PGR) in binding buffer of (A) 1:1, (B) 2:1, (C) 4:1, (D) 8:1, (E) 12:1, and (F) 16:1 (D_h values given as mean ± ½ FWHM).

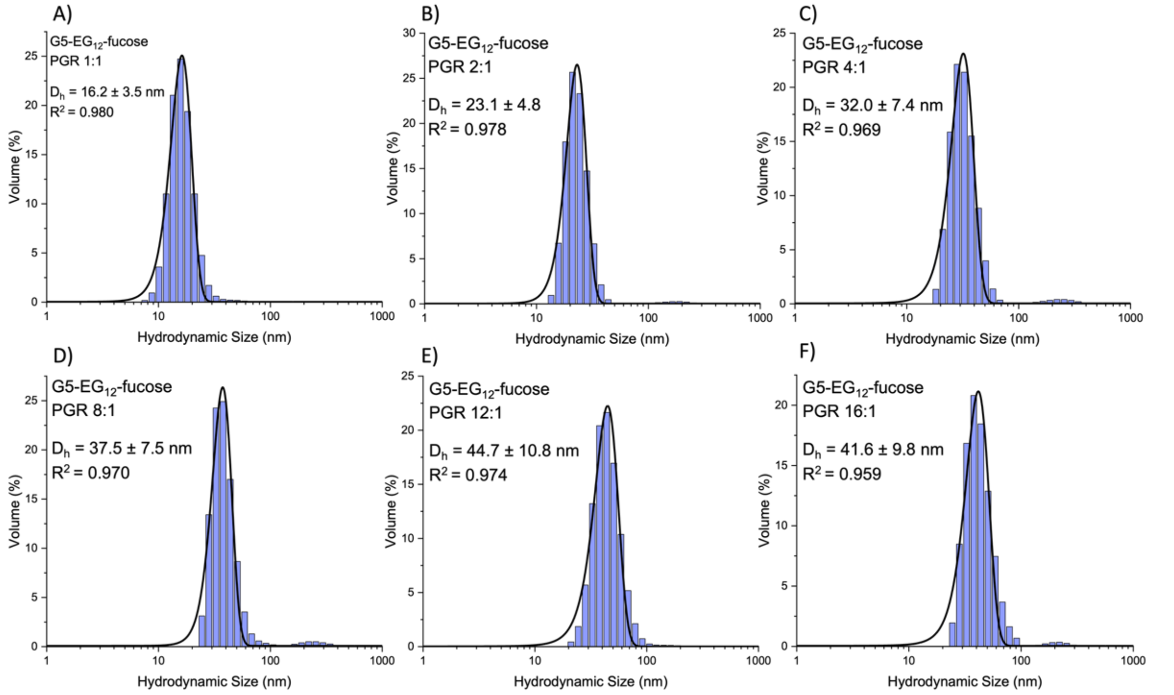

Figure S15. Volume population hydrodynamic size distribution histograms fitted with Gaussian fits for the binding of G5-EG_{12}-fucose (LGMR 1000, 20 nM) with DC-SIGN at varying protein:G5 molar ratios (PGR) in binding buffer of (A) 1:1, (B) 2:1, (C) 4:1, (D) 8:1, (E) 12:1, and (F) 16:1 (D_h values given as mean ± ½ FWHM).

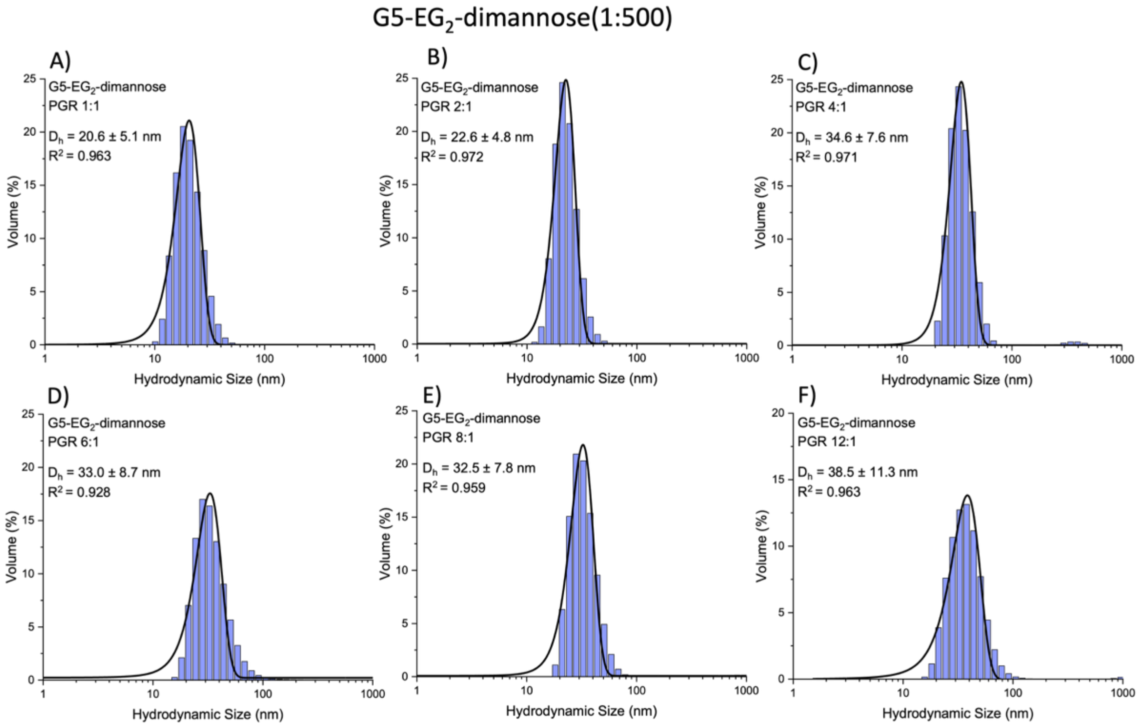

Figure S16. Volume population hydrodynamic size distribution histograms fitted with Gaussian fits for the binding of G5-EG_2-DiMan (LGMR 500, 20 nM) with DC-SIGN at varying protein: G5 molar ratios (PGR) in binding buffer of (A) 1:1, (B) 2:1, (C) 4:1, (D) 8:1, (E) 12:1, and (F) 16:1 (D_h values given as mean ± ½ FWHM).

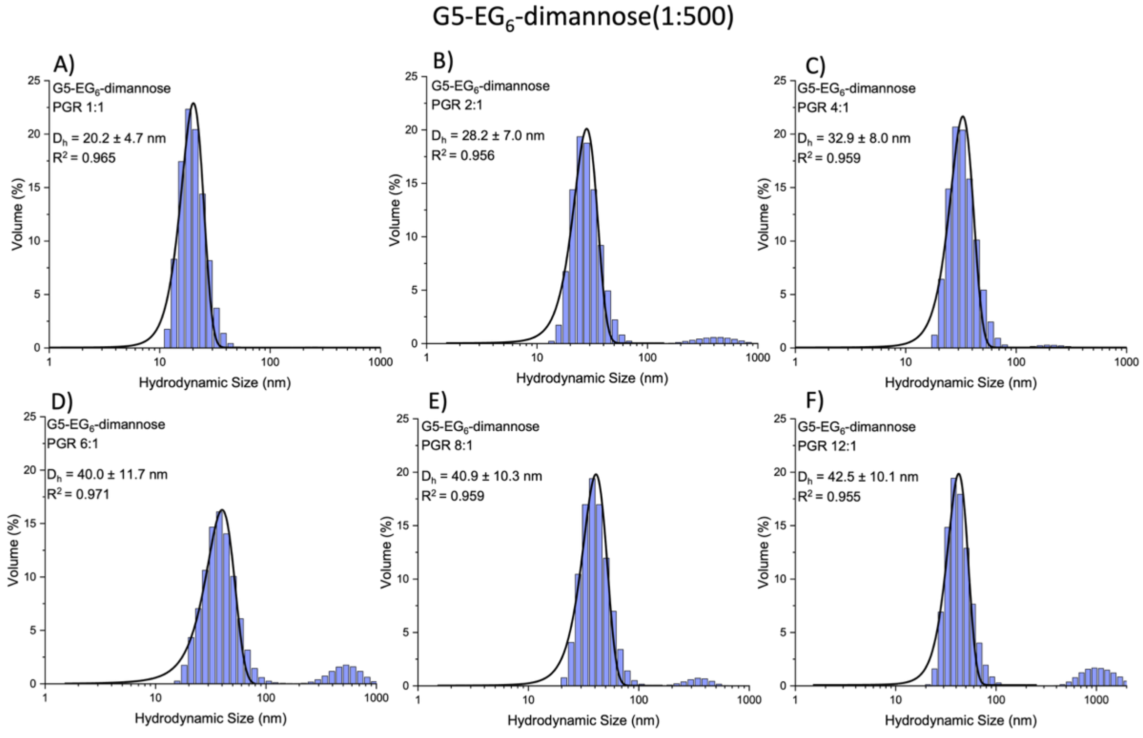

Figure S17. Volume population hydrodynamic size distribution histograms fitted with Gaussian fits for the binding of G5-EG_6-DiMan (LGMR 500, 20 nM) with DC-SIGN at varying protein:G5 molar ratios (PGR) in binding buffer of (A) 1:1, (B) 2:1, (C) 4:1, (D) 8:1, (E) 12:1, and (F) 16:1 (D_h values given as mean ± ½ FWHM).

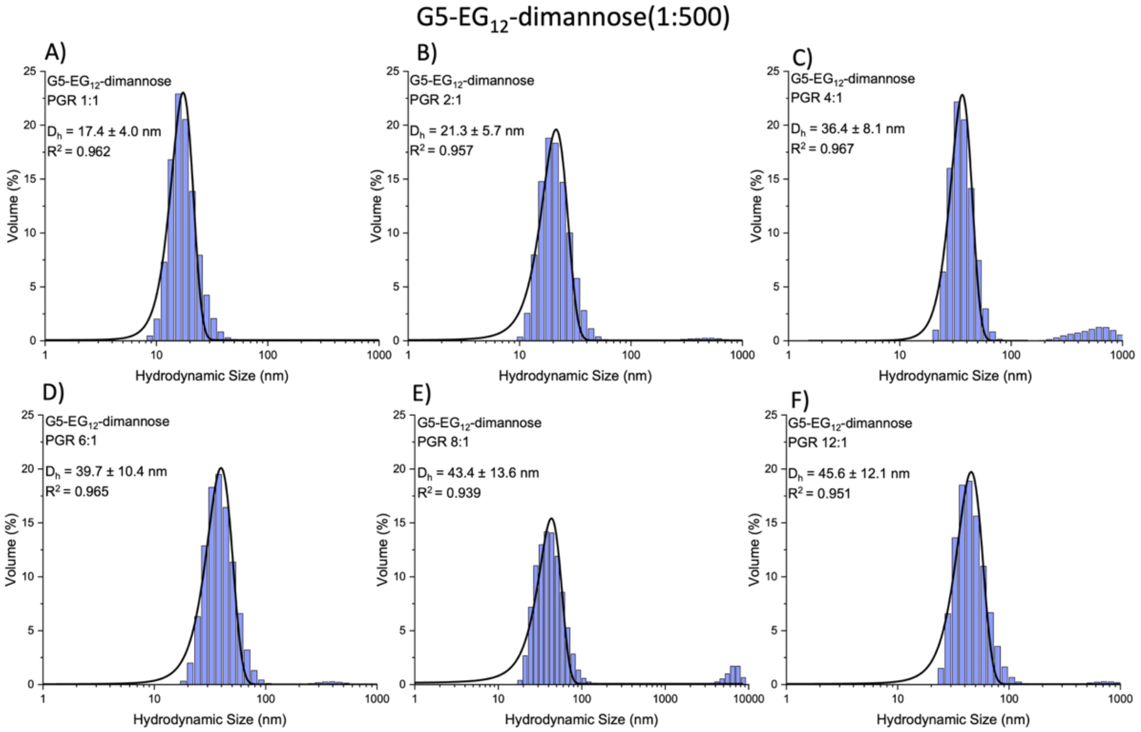

Figure S18. Volume population hydrodynamic size distribution histograms fitted with Gaussian fits for the binding of G5-EG_{12}-DiMan (LGMR 500, 20 nM) with DC-SIGN at varying protein:G5 molar ratios (PGR) in binding buffer of (A) 1:1, (B) 2:1, (C) 4:1, (D) 8:1, (E) 12:1, and (F) 16:1 (D_h values given as mean ± ½ FWHM).

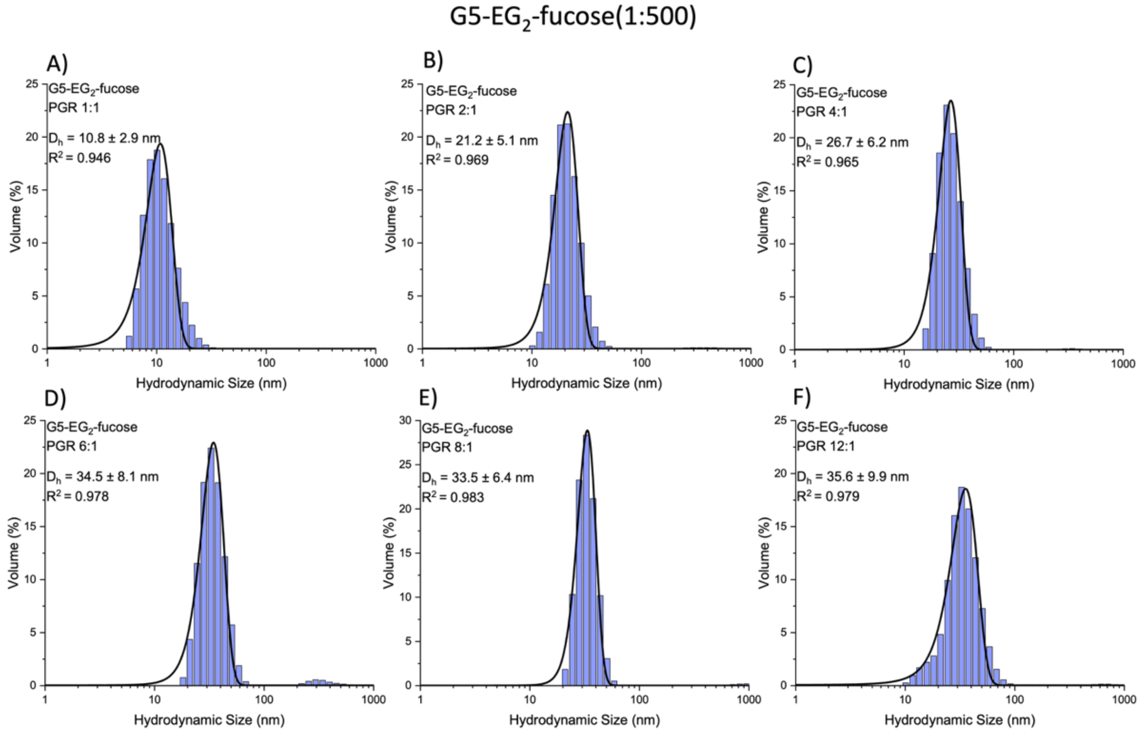

Figure S19. Volume population hydrodynamic size distribution histograms fitted with Gaussian fits for the binding of G5-EG_2-fucose (LGMR 500, 20 nM) with DC-SIGN at varying protein: G5 molar ratios (PGR) in binding buffer of (A) 1:1, (B) 2:1, (C) 4:1, (D) 8:1, (E) 12:1, and (F) 16:1 (D_h values given as mean ± ½ FWHM).

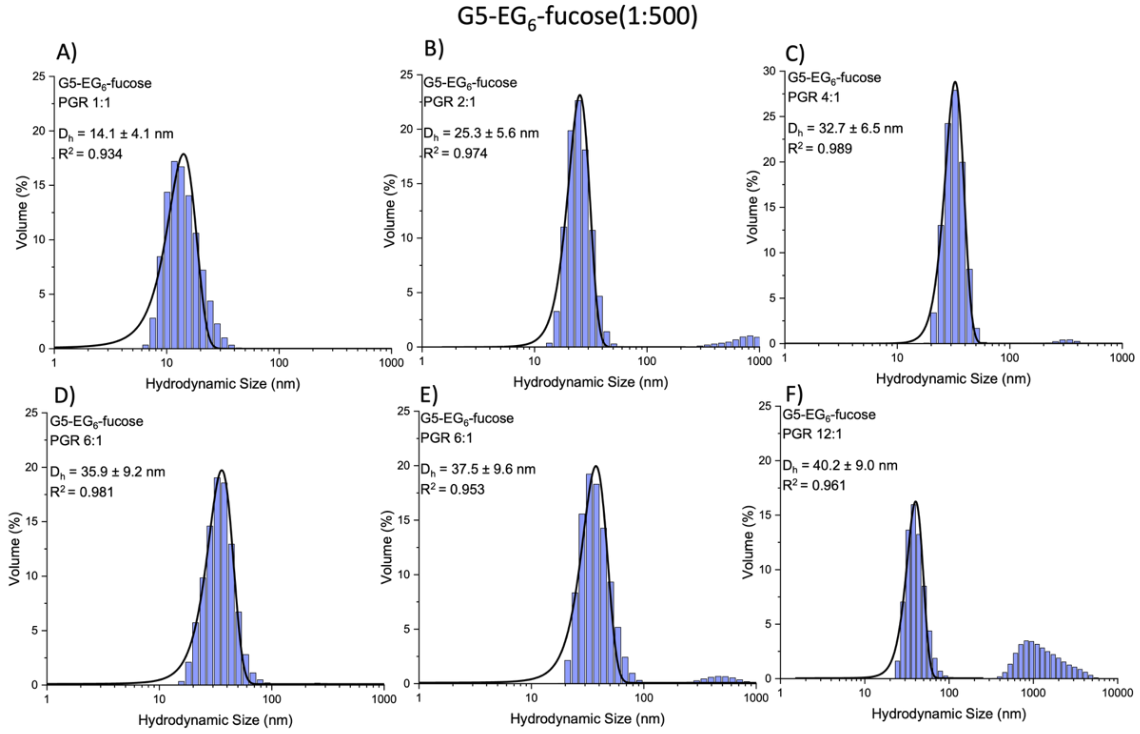

Figure S20. Volume population hydrodynamic size distribution histograms fitted with Gaussian fits for the binding of G5-EG$_6$-fucose (LGMR 500, 20 nM) with DC-SIGN at varying protein:G5 molar ratios (PGR) in binding buffer of (A) 1:1, (B) 2:1, (C) 4:1, (D) 8:1, (E) 12:1, and (F) 16:1 (D_h values given as mean ± ½ FWHM).

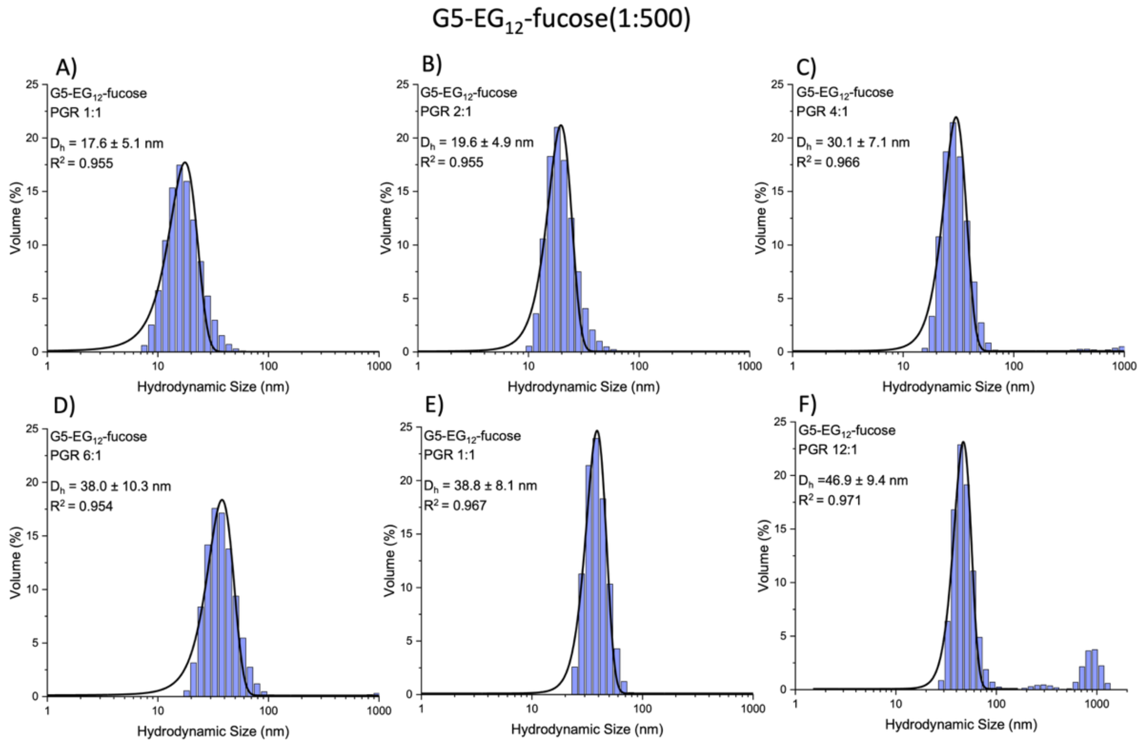

Figure S21. Volume population hydrodynamic size distribution histograms fitted with Gaussian fits for the binding of G5-EG$_{12}$-fucose (LGMR 500, 20 nM) with DC-SIGN at varying protein:G5 molar ratios (PGR) in binding buffer of (A) 1:1, (B) 2:1, (C) 4:1, (D) 8:1, (E) 12:1, and (F) 16:1 (D_h values given as mean ± ½ FWHM).

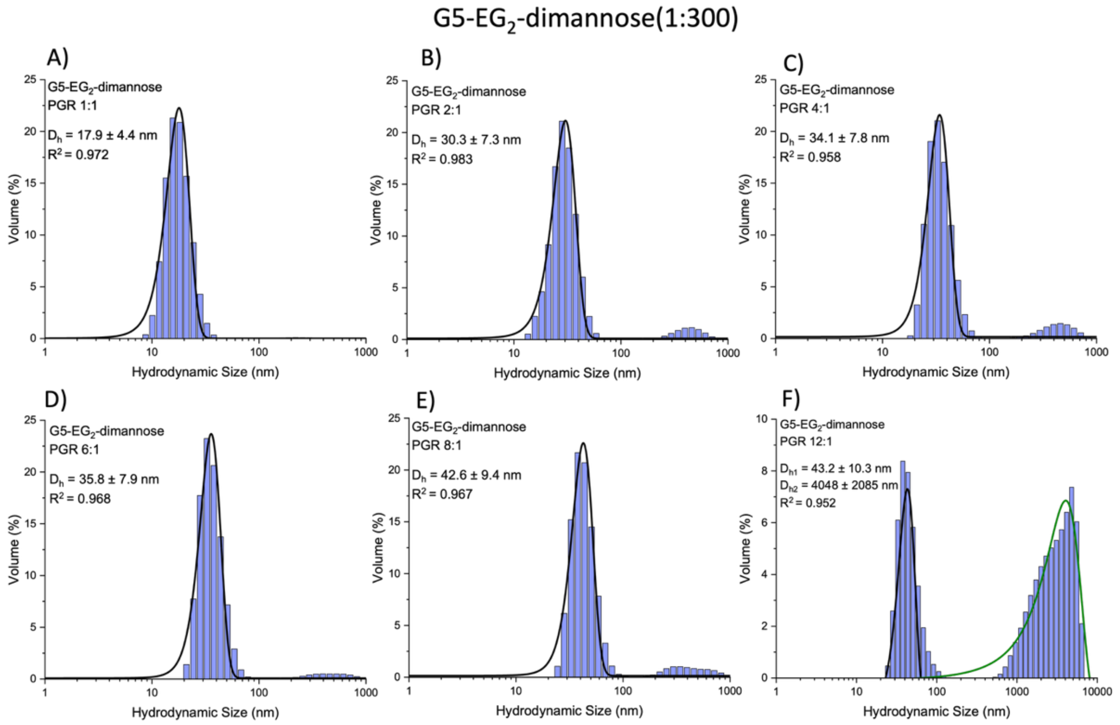

Figure S22. Volume population hydrodynamic size distribution histograms fitted with Gaussian fits for the binding of G5-EG2-DiMan (LGMR 300, 20 nM) with DC-SIGN at varying protein:G5 molar ratios (PGR) in binding buffer of (A) 1:1, (B) 2:1, (C) 4:1, (D) 8:1, (E) 12:1, and (F) 16:1 (D_h values given as mean ± ½ FWHM).

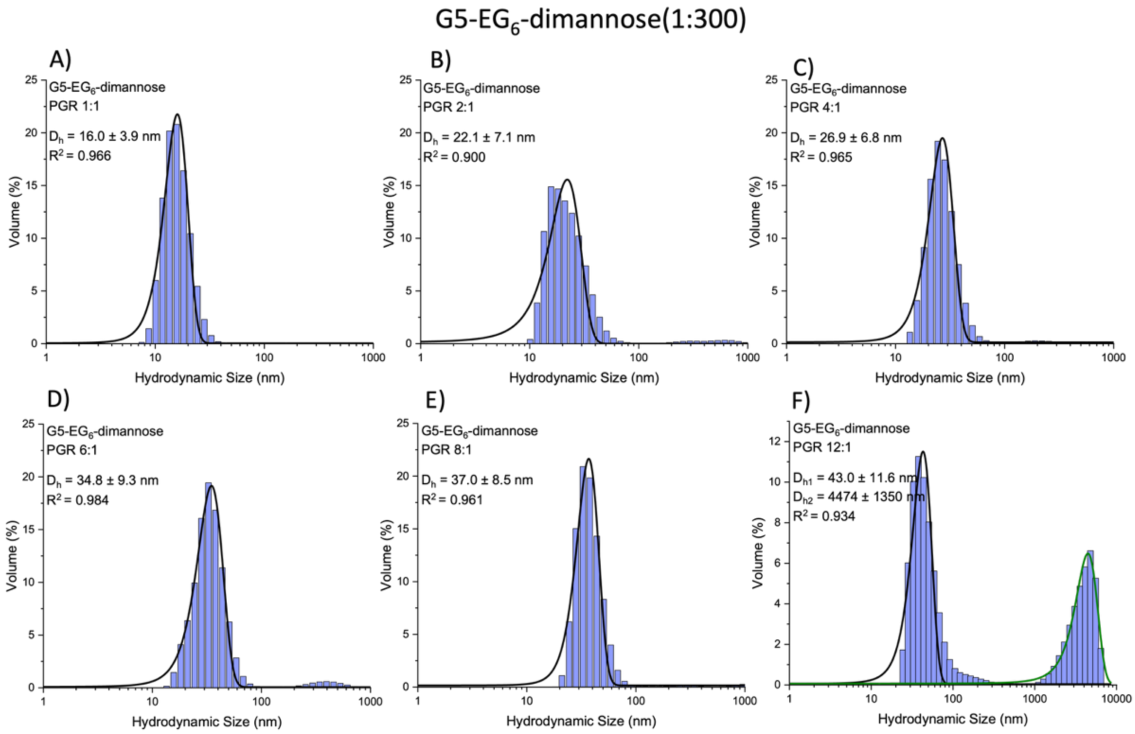

Figure S23. Volume population hydrodynamic size distribution histograms fitted with Gaussian fits for the binding of G5-EG6-DiMan (LGMR 300, 20 nM) with DC-SIGN at varying protein:G5 molar ratios (PGR) in binding buffer of (A) 1:1, (B) 2:1, (C) 4:1, (D) 8:1, (E) 12:1, and (F) 16:1 (D_h values given as mean ± ½ FWHM).

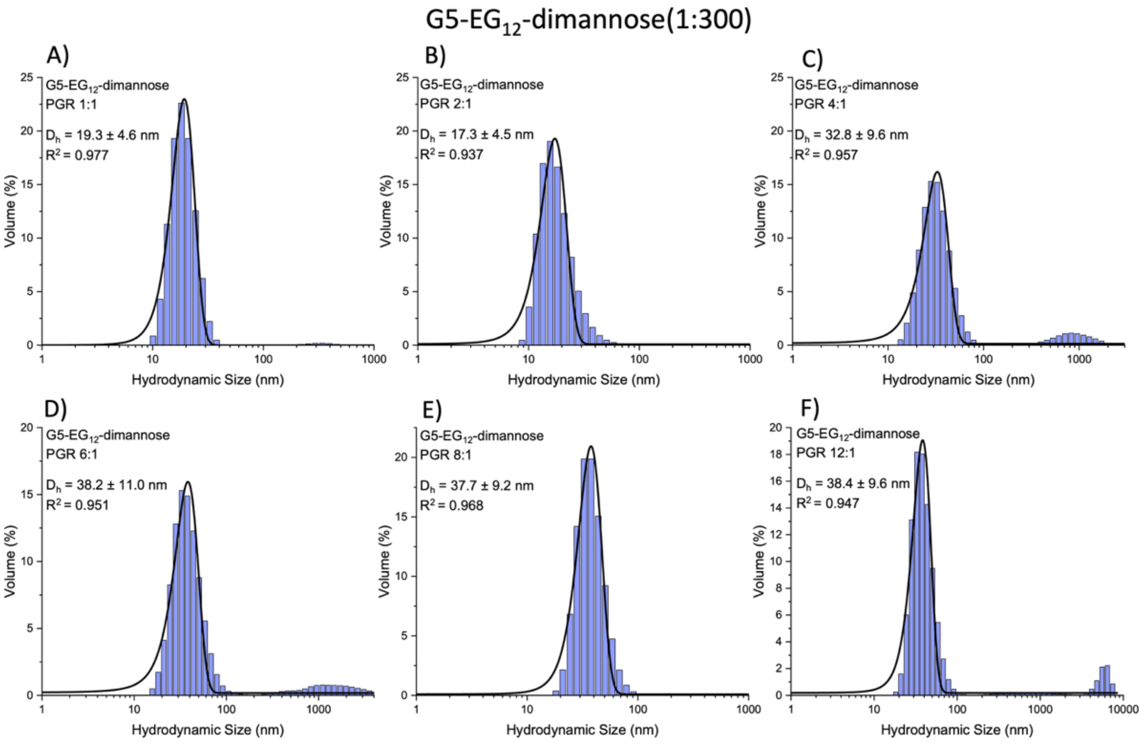

Figure S24. Volume population hydrodynamic size distribution histograms fitted with Gaussian fits for the binding of G5-EG_{12}-DiMan (LGMR 300, 20 nM) with DC-SIGN at varying protein: G5 molar ratios (PGR) in binding buffer of (A) 1:1, (B) 2:1, (C) 4:1, (D) 8:1, (E) 12:1, and (F) 16:1 (D_h values given as mean ± ½ FWHM).

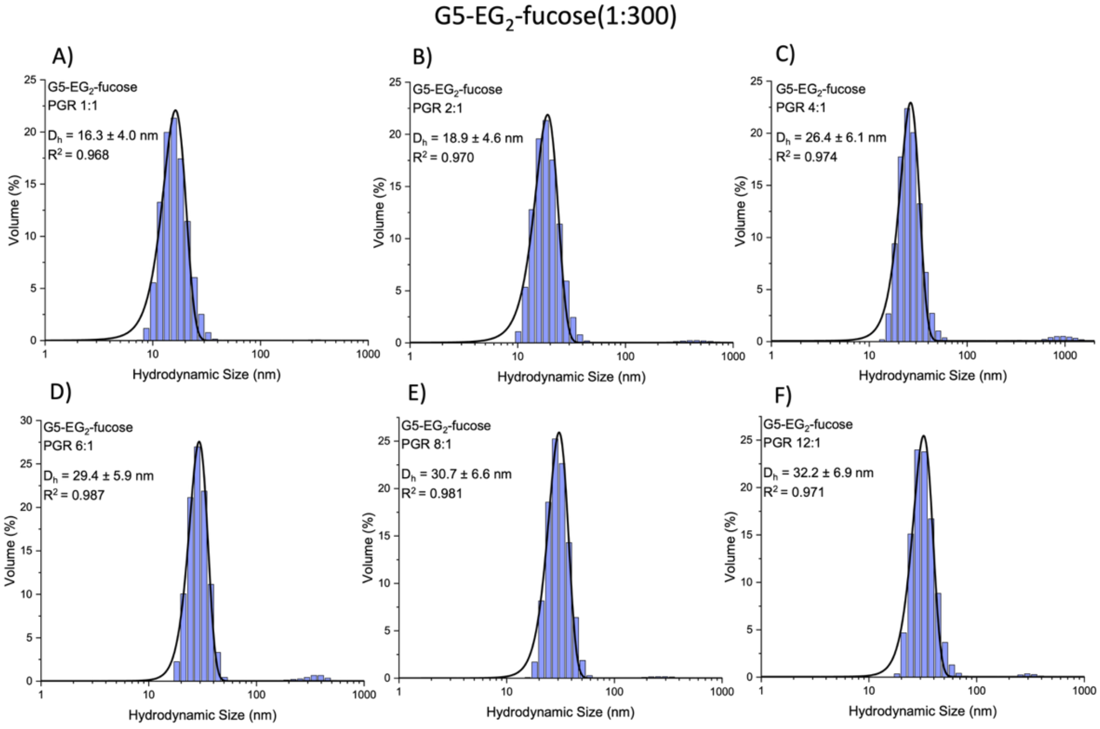

Figure S25. Volume population hydrodynamic size distribution histograms fitted with Gaussian fits for the binding of G5-EG_2-fucose (LGMR 300, 20 nM) with DC-SIGN at varying protein:G5 molar ratios (PGR) in binding buffer of (A) 1:1, (B) 2:1, (C) 4:1, (D) 8:1, (E) 12:1, and (F) 16:1 (D_h values given as mean ± ½ FWHM).

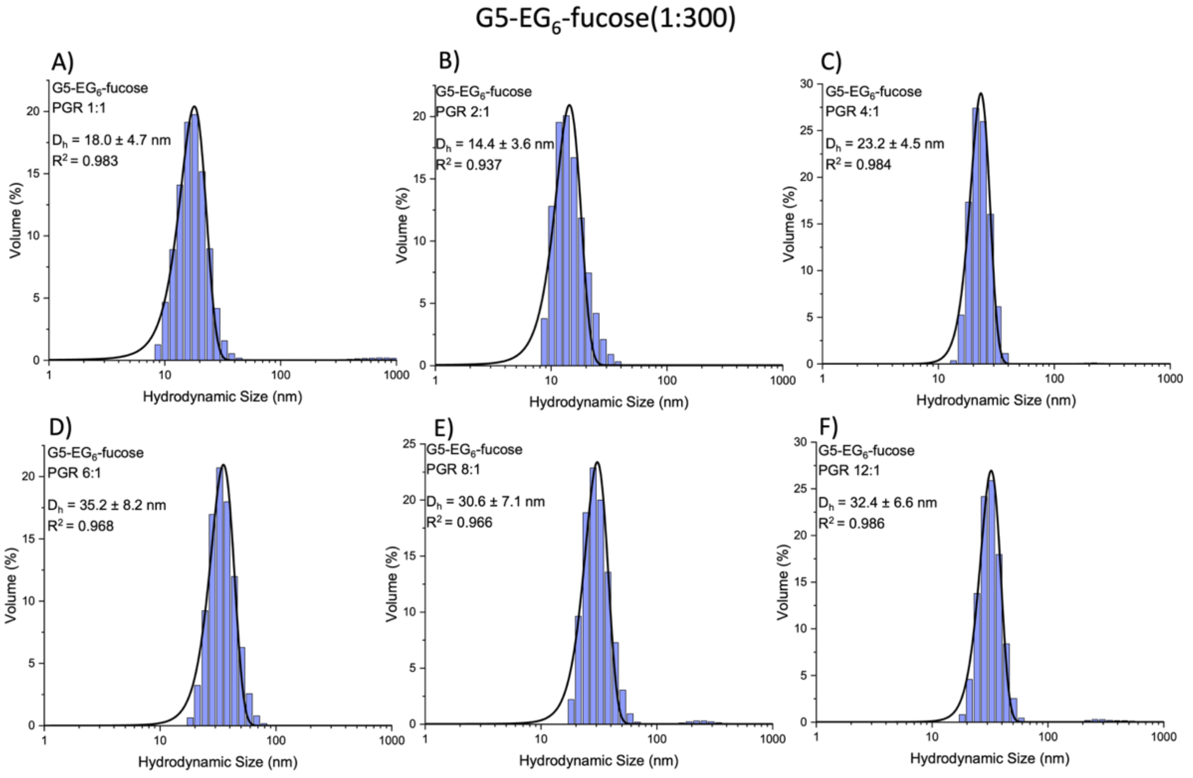

Figure S26. Volume population hydrodynamic size distribution histograms fitted with Gaussian fits for the binding of G5-EG6-fucose (LGMR 300, 20 nM) with DC-SIGN at varying protein:G5 molar ratios (PGR) in binding buffer of (A) 1:1, (B) 2:1, (C) 4:1, (D) 8:1, (E) 12:1, and (F) 16:1 (D_h values given as mean ± ½ FWHM).

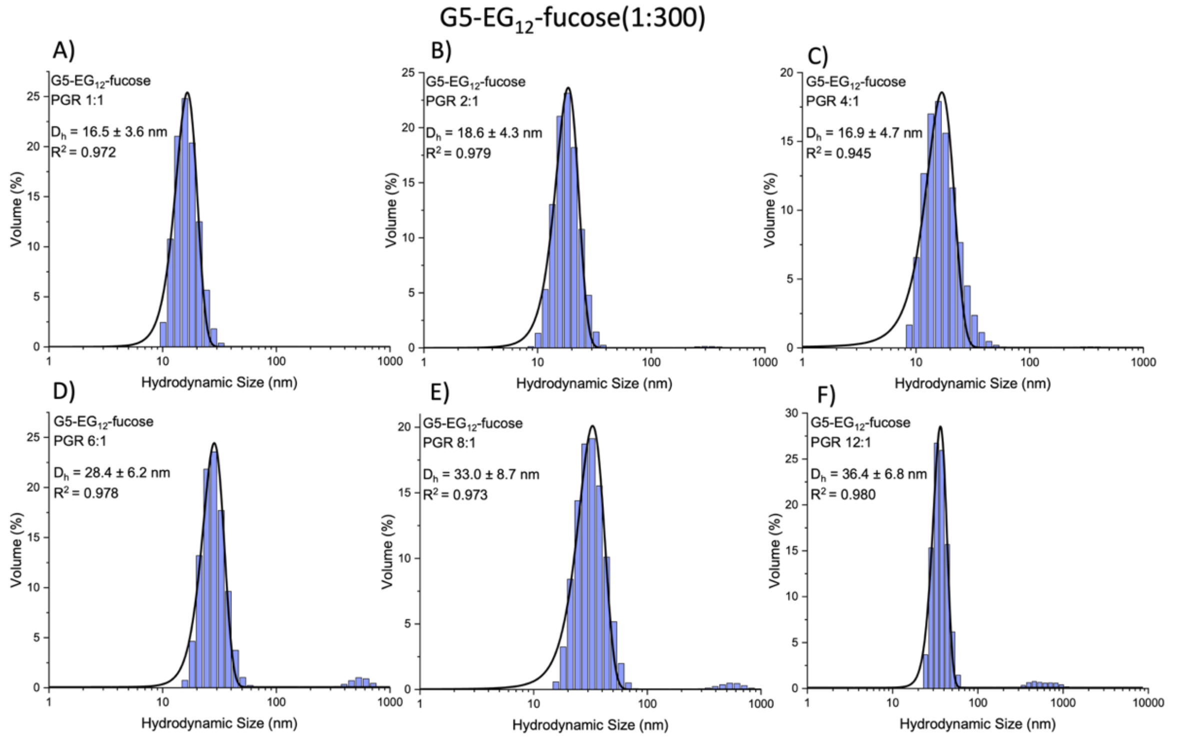

Figure S27. Volume population hydrodynamic size distribution histograms fitted with Gaussian fits for the binding of G5-EG12-fucose (LGMR 300, 20 nM) with DC-SIGN at varying protein: G5 molar ratios (PGR) in binding buffer of (A) 1:1, (B) 2:1, (C) 4:1, (D) 8:1, (E) 12:1, and (F) 16:1 (D_h values given as mean ± ½ FWHM).

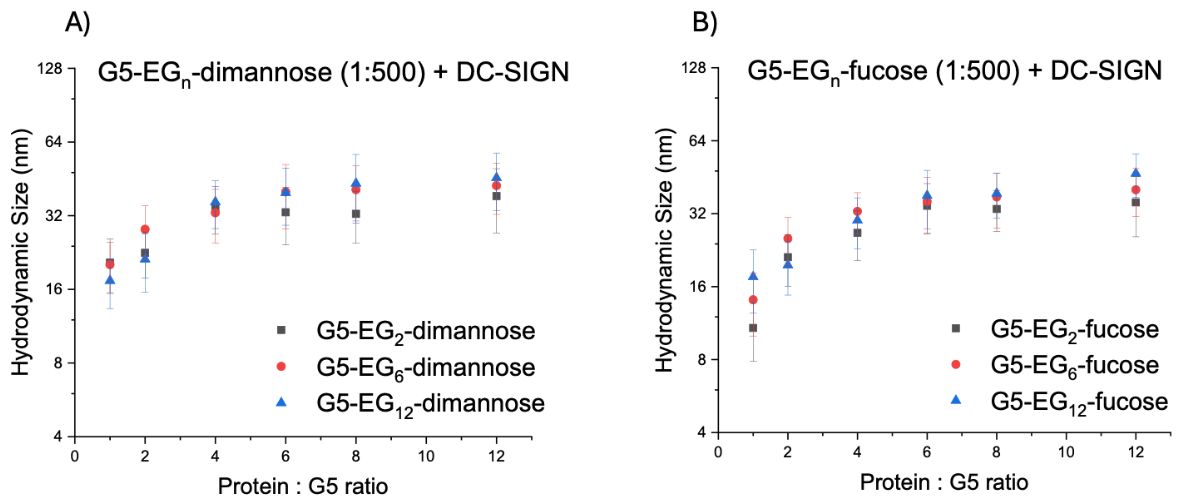

Figure S28. Hydrodynamic diameter (D_h, volume population) - PGR relationship for A) G5-EGn-DiMan (LGMR 500) with DC-SIGN and B) G5-EGn-fucose (LGMR 500) with DC-SIGN.

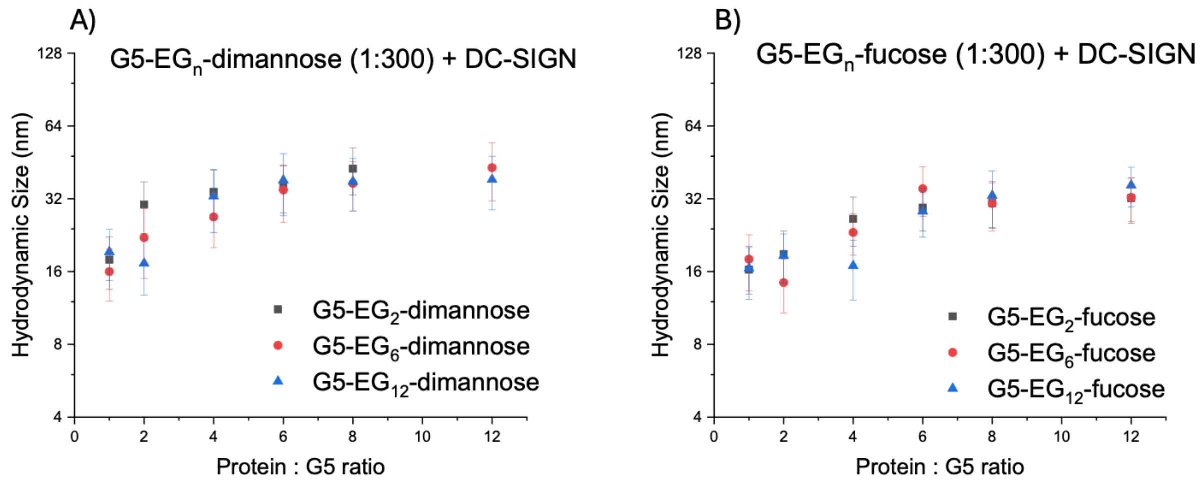

Figure S29. Hydrodynamic diameter (D_h, volume population) - PGR relationship of monolayer species for A) G5-EGn-DiMan (LGMR 300) with DC-SIGN and B) G5-EGn-fucose (LGMR 300) with DC-SIGN.

7. QE vs C plots and data tables for G5-glycans binding to DC-SIGN

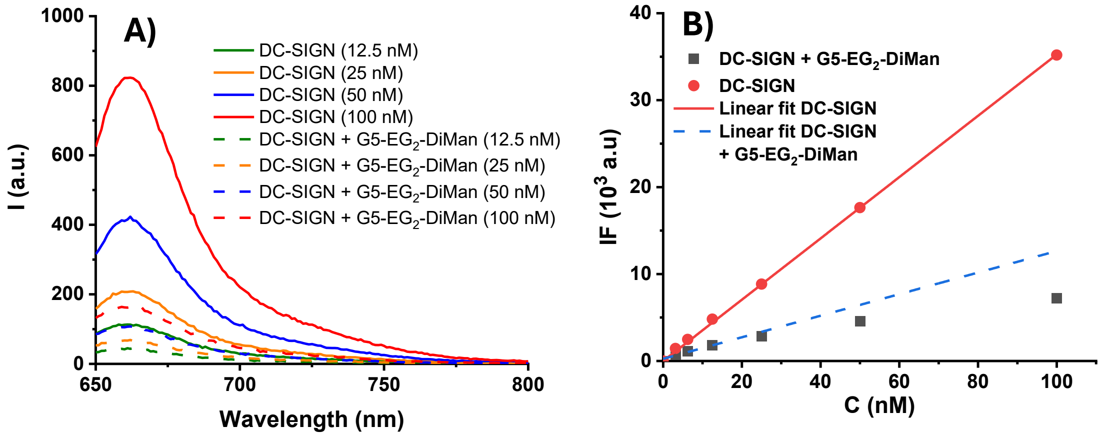

Figure S30. A) Fluorescence spectra of varying concentrations of Atto-643 labelled DC-SIGN in the absence (solid lines) and presence (broken lines) of one molar equivalent of G5-EG2-DiMan. **B)** Relationship between the integrated fluorescence and concentration in the absence (red dots) and presence (black squares) of one molar equivalent of G5-EG$_2$-DiMan. In the absence of G5-EG$_2$-DiMan, the fluorescence is fitted well by a linear relationship, but after addition of 1 mol. equiv. of G5-EG$_2$-DiMan, the relationship deviates increasingly more from linear with the increasing concentration, indicating a greater proportion of DC-SIGN is bound to G5-ED$_2$-DiMan and get quenched with the increasing concentration.

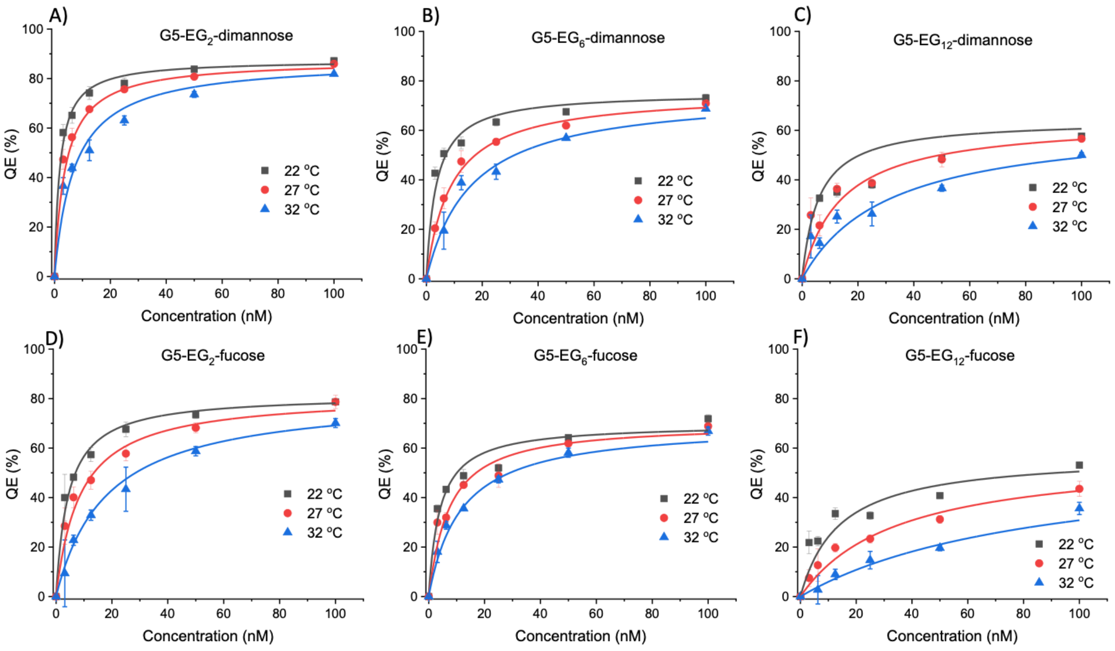

Figure S31. The quenching efficiency (QE) - concentration relationship for DC-SIGN binding to G5-glycans made in 1000 LGMR at 22 °C (grey), 27 °C (red) and 32°C (blue) for **A**) G5-EG2-DiMan, **B**) G5-EG6-DiMan, **C**) G5-EG12-DiMan, **D**) G5-EG2-fucose, **E**) G5-EG6-fucose, and **F**) G5-EG12-fucose. Error bars represent the standard deviations (SDs) of duplicate experiments at each concentration.

Table S4. Summary of the fitting parameters of DC-SIGN binding to G5-EGn-glycans prepared at LGMR of 1000. Errors represent the standard deviations observed by the fitting.

G5-EGn-Glycan	Temperature (°C)	K_d (nM)	QE_{max} (%)	R^2
G5-EG2-DiMan	22	1.9±0.4	87.5±0.5	1
	27	3.8±03		1
	32	7.1±0.7		0.999
G5-EG6-DiMan	22	3.5±0.7	75.3±1.6	0.999
	27	8.9±1.5		0.998
	32	16.0±1.5		1
G5-EG12-DiMan	22	5.8±1.0	64.2±3.4	0.955
	27	14.0±6.1		1
	32	30.9±17.1		0.994
G5-EG2-fucose	22	4.4±0.2	81.6±0.7	0.999
	27	8.5±0.8		0.997
	32	17.9±1.1		0.999
G5-EG6-fucose	22	3.9±0.6	69.8±2.1	0.999
	27	7.1±0.6		0.997
	32	11.5±1.1		0.999
G5-EG12-fucose	22	13.9±3.4	57.6±3.0	0.992
	27	34.8±6.4		0.987
	32	86.6±15.1		0.987

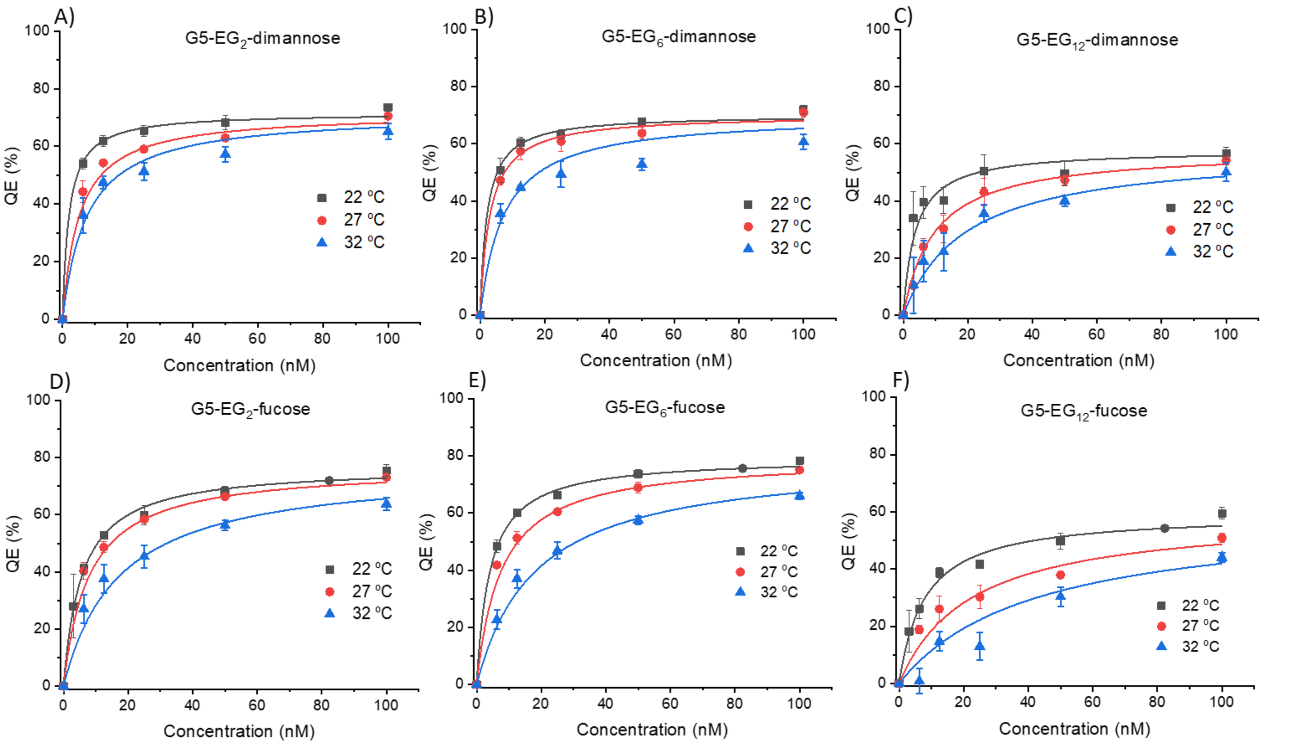

Figure S32. The quenching efficiency (QE) - concentration relationship for DC-SIGN binding to G5-glycans made in a LGMR of 500 at 22 °C (grey), 27 °C (red) and 32°C (blue) for A) G5-EG_2-DiMan, B) G5-EG_6-DiMan, C) G5-EG_{12}-DiMan, D) G5-EG_2-fucose, E) G5-EG_6-fucose, and F) G5-EG_{12}-fucose. Error bars represent the standard deviations (SDs) of duplicate experiments at each concentration.

Table S5. Summary of the fitting parameters of DC-SIGN binding to G5-EG_n-glycans (LGMR = 500). Errors represent the standard deviation as observed by the fitting.

G5-EG_n-Glycan	Temperature (°C)	K_d (nM)	QE_{max} (%)	R^2
G5-EG_2-DiMan	22	2.0±0.5	71.6±1.3	0.999
	27	5.1±0.6		0.999
	32	7.7±1.4		0.996
G5-EG_6-DiMan	22	2.1±0.7	70.2±1.4	0.999
	27	3.0±0.5		0.999
	32	7.3±0.6		0.996
G5-EG_{12}-DiMan	22	3.7±0.8	50.9±1.2	0.996
	27	9.6±1.3		0.999
	32	19.1±2.0		0.996
G5-EG_2-fucose	22	5.6±0.4	77.0±1.2	1
	27	7.9±0.5		1
	32	17.2±1.9		0.998
G5-EG_6-fucose	22	4.1±0.6	79.4±1.2	0.999
	27	7.6±0.6		0.999
	32	18.2±2.1		0.999
G5-EG_{12}-fucose	22	7.9±1.9	59.4±3.2	0.995
	27	22.1±4.5		0.989
	32	41.8±10.1		0.987

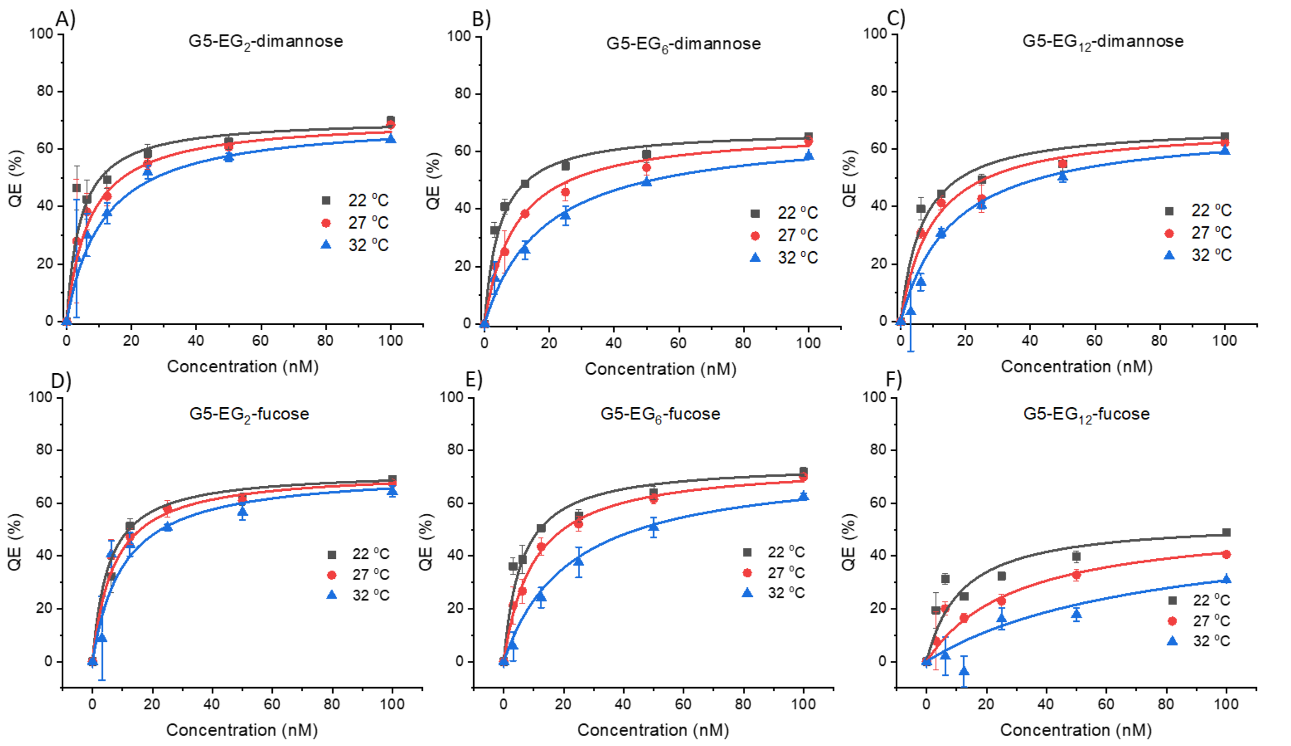

Figure S33. The quenching efficiency - concentration relationship for DC-SIGN binding to G5-glycans made in a LGMR of 300 at 22 °C (grey), 27 °C (red) and 32°C (blue) for A) G5-EG_2-DiMan, B) G5-EG_6-DiMan, C) G5-EG_{12}-DiMan, D) G5-EG_2-fucose, E) G5-EG_6-fucose, and F) G5-EG_{12}-fucose. Error bars represent the standard deviations (SDs) of duplicate experiments at each concentration.

Table S6. Summary of the fitting parameters of DC-SIGN binding to G5-EG_n-glycans (LGMR 300). Errors represent the standard deviation as observed by the fitting.

G5-EGn-Glycan	Temperature (°C)	K_d (nM)	QE*max*	R^2
G5-EG_2-DiMan	22	4.3±0.8	70.1±1.0	0.997
	27	7.1±0.9		0.999
	32	11.2±1.4		1
G5-EG_2-DiMan	22	3.8±0.5	67.8±1.1	0.999
	27	7.2±1.0		0.998
	32	16.2±1.7		1
G5-EG_2-DiMan	22	4.7±0.4	68.5±0.9	0.999
	27	9.3±0.5		0.999
	32	20.0±1.4		1
G5-EG_2-fucose	22	5.4±1.0	72.3±0.8	1
	27	7.1±1.1		1
	32	10.1±1.0		0.998
G5-EG_6-fucose	22	6.0±0.6	67.8±1.1	0.999
	27	9.9±1.5		0.998
	32	22.4±3.3		1
G5-EG_{12}-fucose	22	11.6±2.6	53.7±0.03	0.994
	27	30.0±5.7		0.994
	32	74.0±9.0		0.998

8. Van't Hoff plots for G5-EG$_n$-glycans binding to DC-SIGN thermodynamics

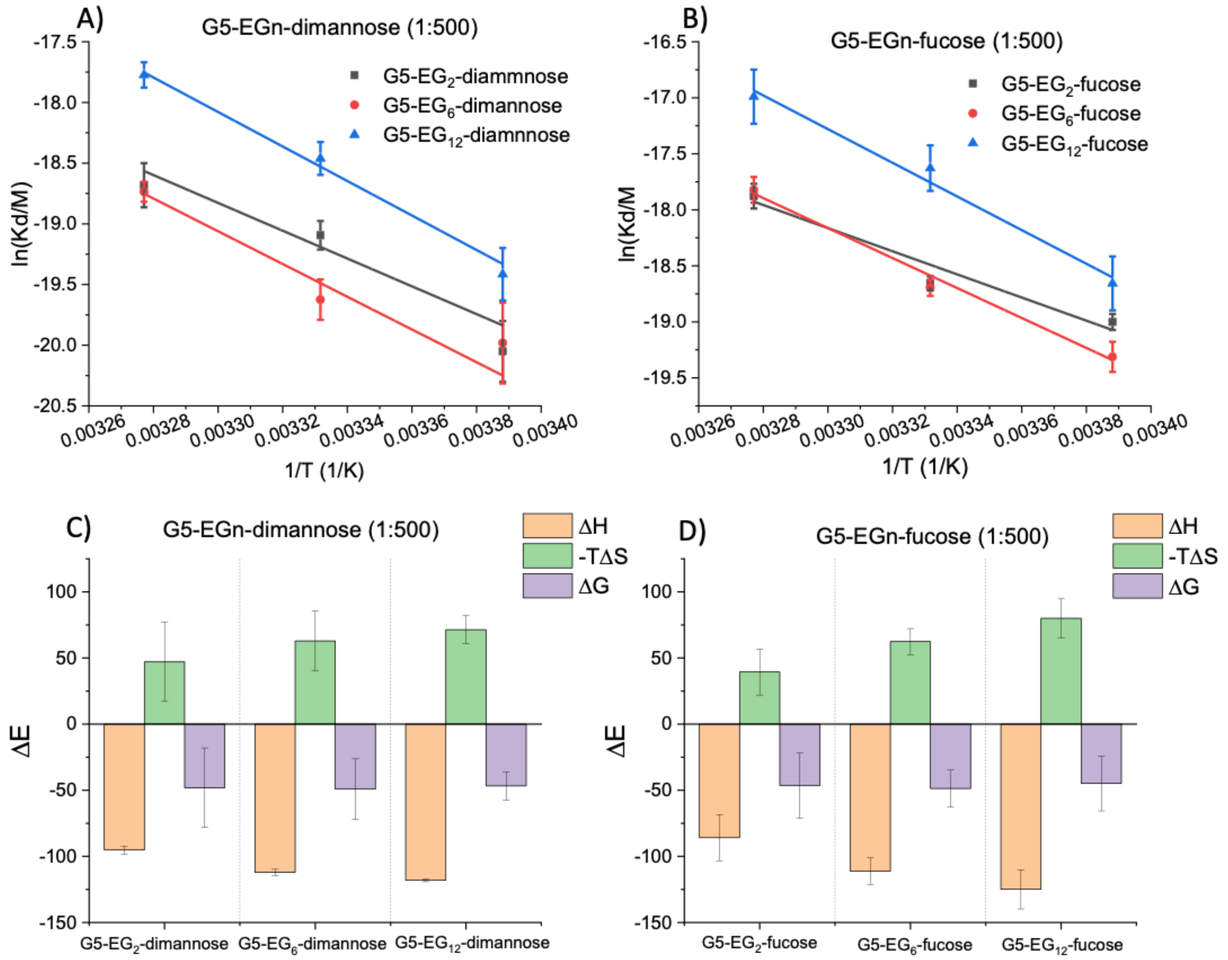

Figure S34. van't Hoff analyses of the ln(K_d)–(1/T) relationships for DC-SIGN binding with G5-EG$_n$-glycans prepared at a LGMR of 500. **A**) G5-EG$_2$-DiMan (grey), G5-EG$_6$-DiMan (red), G5-EG$_{12}$-DiMan (blue) or **B**) G5-EG$_2$-fucose (grey), G5-EG$_6$-fucose (red), G5-EG$_{12}$-fucose (blue). **C**) Comparison of the standard (T = 298 K) enthalpy (orange), entropy (green), and Gibbs free energy (purple) changes of G5-EG$_n$-DiMan (LGMR 500) binding with DC-SIGN. **D**) Comparison of the standard (T = 298 K) enthalpy (orange), entropy (green), and Gibbs free energy (purple) changes of G5-EG$_n$-Fucose (LGMR 500) binding with DC-SIGN. SDs represent fitting errors.

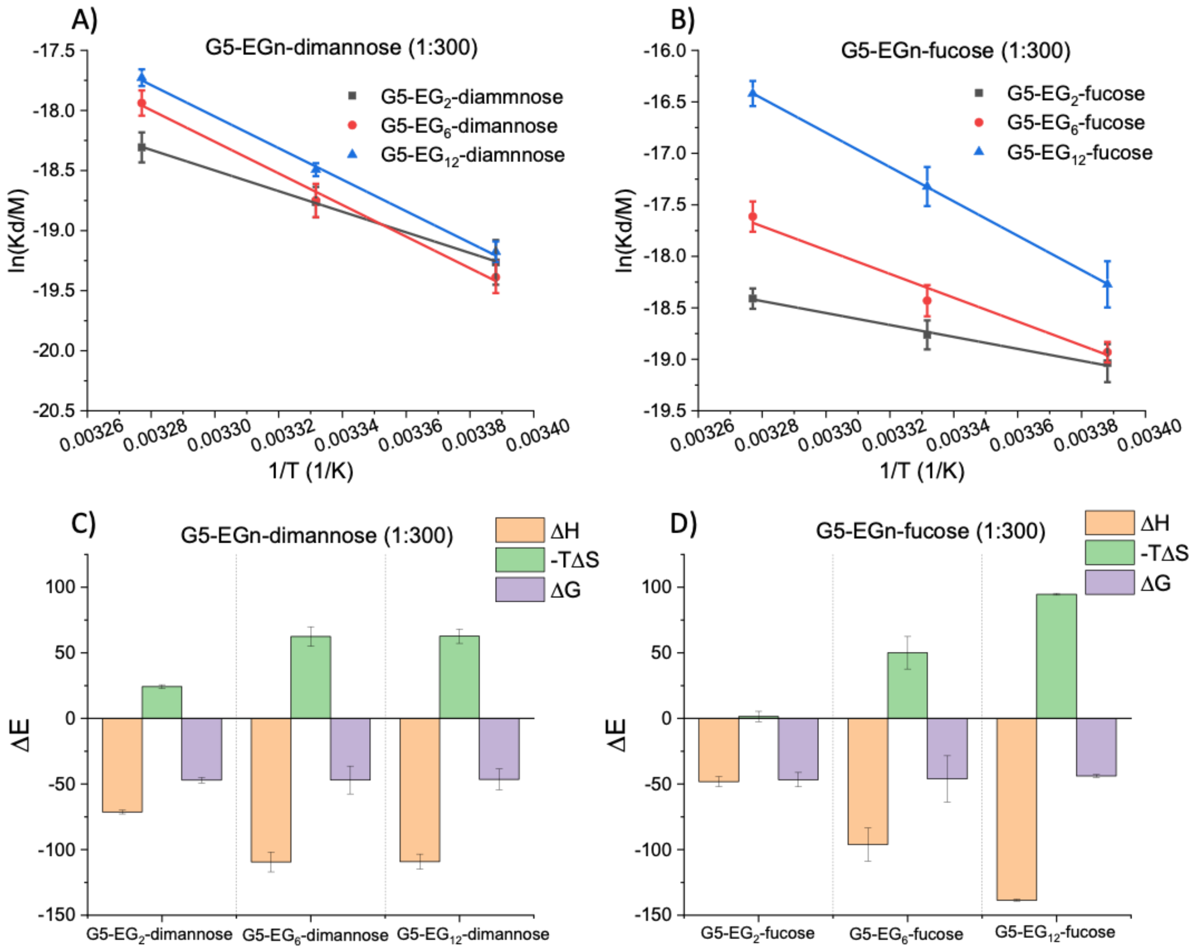

Figure S35. van't Hoff analyses of the ln(K_d)–(1/T) relationships for DC-SIGN binding with G5-EG_n-glycans prepared at a LGMR of 300. **A**) G5-EG_2-DiMan (grey), G5-EG_6-DiMan (red), G5-EG_{12}-DiMan (blue) or B) G5-EG_2-fucose (grey), G5-EG_6-fucose (red), G5-EG_{12}-fucose (blue). C) Comparison of the standard (T = 298 K) enthalpy (orange), entropy (green), and Gibbs free energy (purple) changes of G5-EG_n-DiMan (LGMR 300) binding with DC-SIGN. **D**) Comparison of the standard (T = 298 K) enthalpy (orange), entropy (green), and Gibbs free energy (purple) changes of G5-EG_n-Fucose (LGMR 300) binding with DC-SIGN. SDs represent fitting errors.

9. Unprocessed viral inhibition and cytotoxicity data

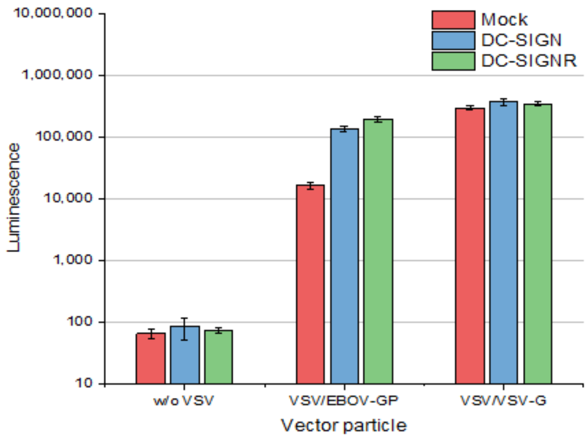

Figure S36. In the absence of inhibitors and after transfection with DC-SIGN (blue) or DC-SIGNR (green) in 293T cells, entry of VSV/EBOV-GP is increased 10-fold, reflected in the luciferase activity/ luminescence, compared to cells transfected with empty plasmid (mock, red). Entry of the control VSV/VSV-G is not augmented by DC-SIGN/R transfection. When no VSV particle (encoding the luciferase gene) is present, luminescence is very low (>1000 fold lower than the positive controls), confirming that cellular luminescence arises from infectious entry of VSV vector particles.

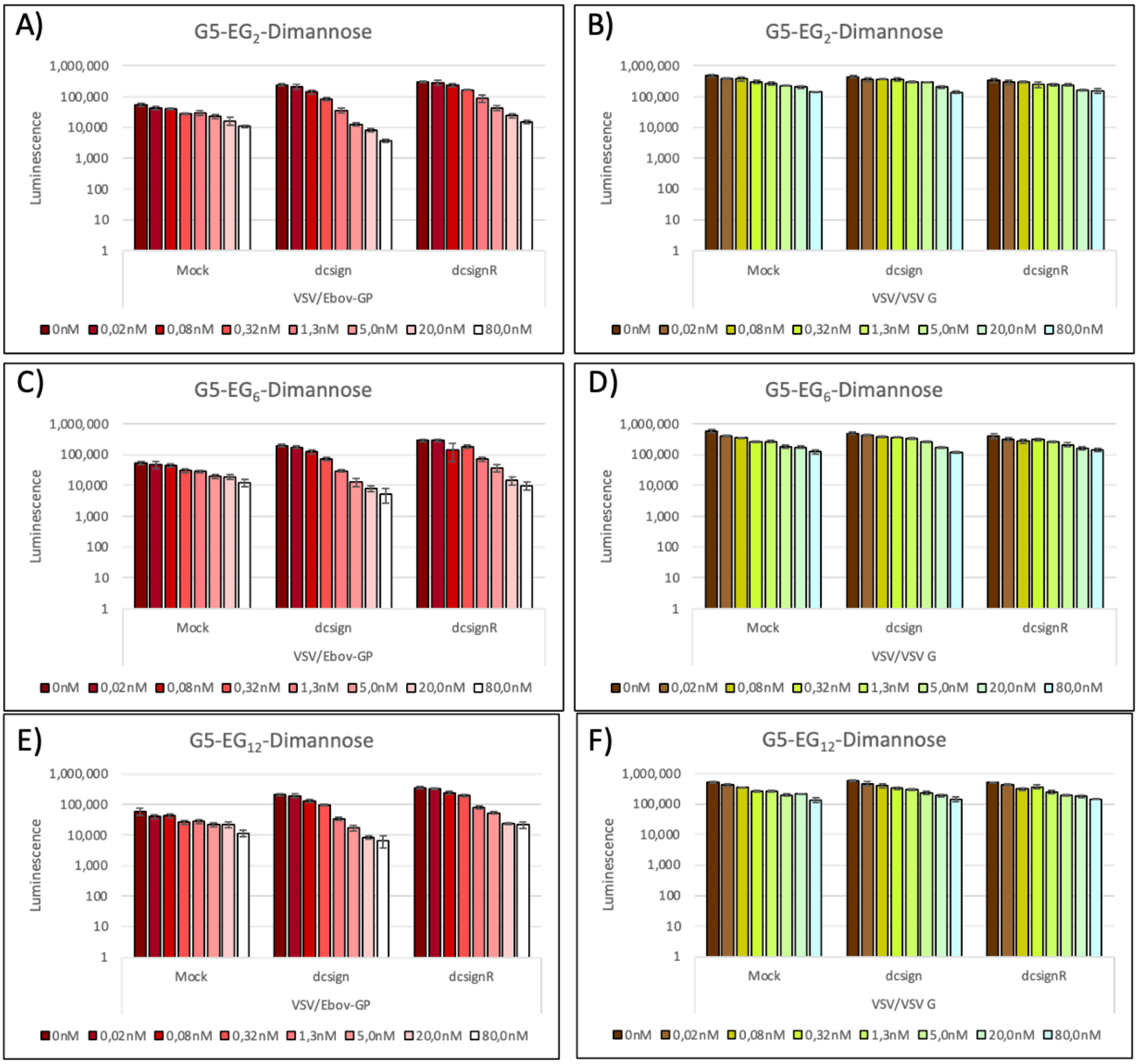

Figure S37. Comparison of cellular luciferase activities of 293T cells after being transfected with empty plasmid (mock) or DC-SIGN- or DC-SIGNR-encoding plasmid, pre-incubated with varying concentrations of G5-EG$_n$-DiMan and inoculated with VSV reporter particles bearing the EBOV-GP (**A, C, E**,) or the control VSV-G (**B, D, F**). Luciferase activities in cell lysates were measured at 24 hours post-infection. The results of a single experiment performed with technical quadruplicates are shown and were confirmed in a separate experiment. Error bars indicate the SDs of the quadruplicate samples.

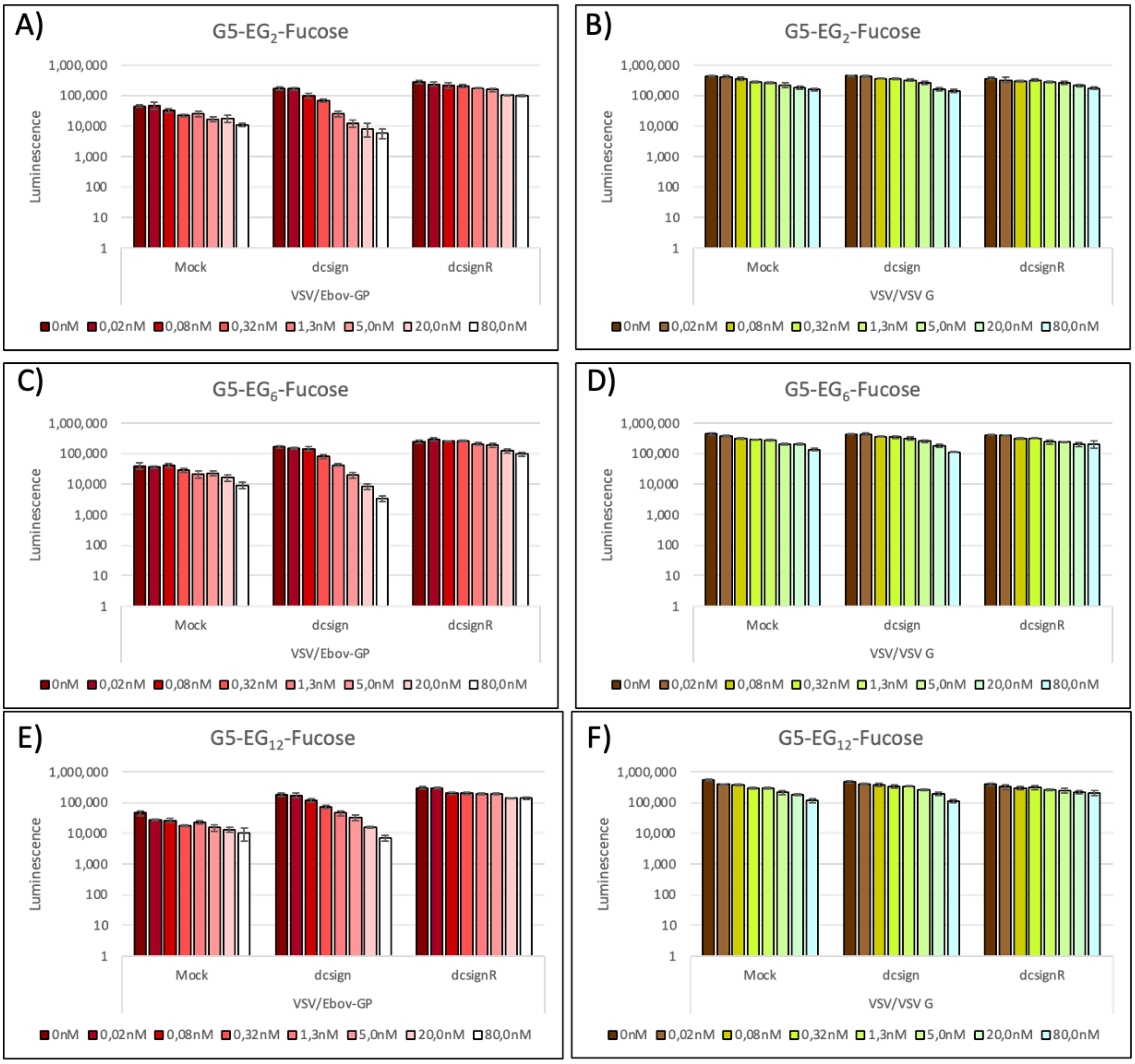

Figure S38. Comparison of cellular luciferase activities of 293T cells after being transfected with empty plasmid (mock) or DC-SIGN- or DC-SIGNR-encoding plasmid, pre-incubated with varying concentrations of G5-EG$_n$-fucose and inoculated with VSV reporter particles bearing the EBOV-GP (**A, C, E**) or the VSV-G control (**B, D, F**). Luciferase activities in cell lysates were measured after 24 hours post-transduction. The results of a single experiment performed with technical quadruplicates are shown and were confirmed in a separate experiment. Error bars indicate the SDs of the quadruplicate samples.

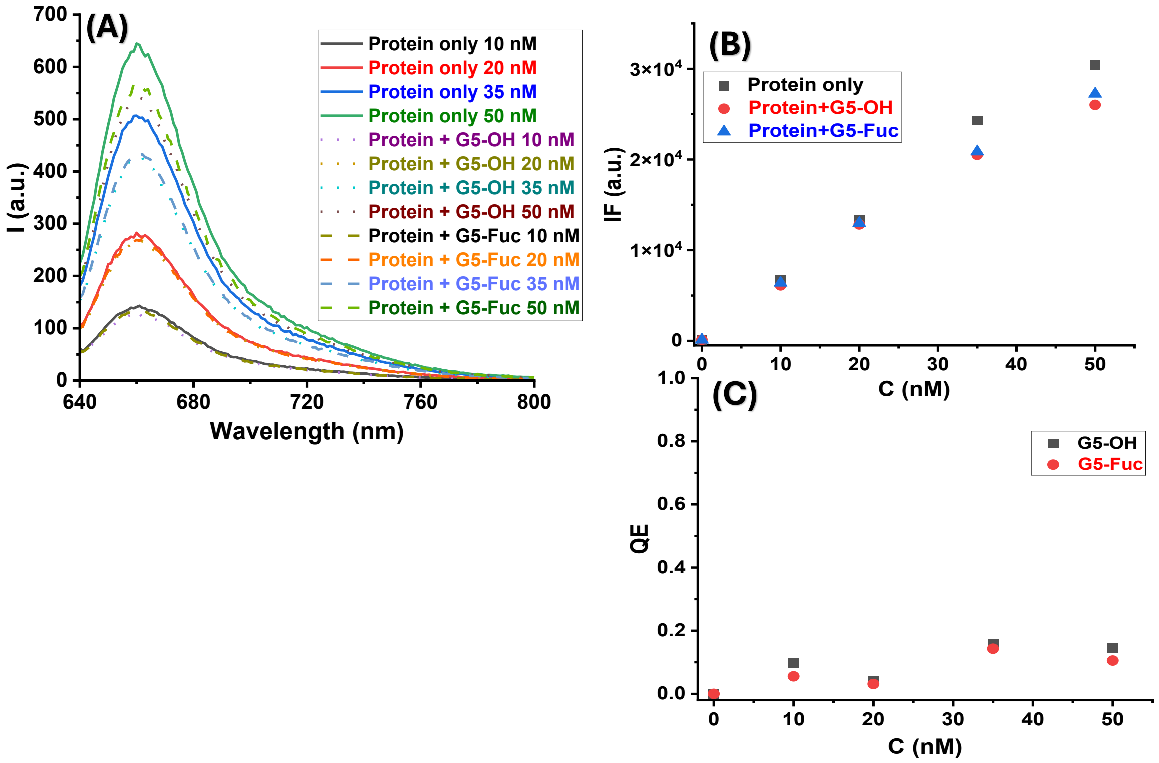

Figure S39. Probing DC-SIGNR interactions with G5-Fuc *via* GNP NSET quenching assay. Both G5-Fuc and G5-OH negative control were prepared at a LGMR of 500, the same as those used in blocking viral cellular infection studies, using LA-EG$_2$-Fuc or LA-EG$_2$-OH control ligand (LA-EG$_2$-OH has the same chemical structure as LA-EG$_2$-Fuc, except replacing the terminal Fuc group in the latter with an OH group).

(**A**) Fluorescence spectra of varying concentration of Atto-643-labeled DC-SIGNR in the absence (solid lines) and in the presence of equal molar amount of the G5-OH negative control (dotted lines) or G5-Fuc (broken lines). (**B**) Comparison of integrated fluorescence intensity (IF) of Atto-643-labeled DC-SIGNR only (black squares), DC-SIGNR + G5-OH control (red dots) and DC-SIGNR + G5-Fuc (blue triangles) at different concentrations. (C) Comparison of quenching efficiency (QE) for Atto-643-labeled DC-SIGNR interaction with the G5-OH negative control (black squares) and G5-Fuc (red dots) at different concentrations.

While low levels of QEs were observed for DC-SIGNR after mixing with G5-Fuc, their QEs were the same or even smaller than those of the G5-OH control. The G5-OH control, coated with LA-EG$_2$-OH ligand, does not bind to DC-SIGN/R, and is effective in resisting nonspecific interactions with proteins (Ning et al., *JACS Au*, 2024, **4**, 3295). Thus, the small QEs observed here are due to GNP's inner filter effect. This result indicates no detectable specific interactions between G5-Fuc and DC-SIGNR under our experimental conditions. It explains the lack of inhibition for G5-EG$_n$-Fuc against DC-SIGNR mediated viral infections above.

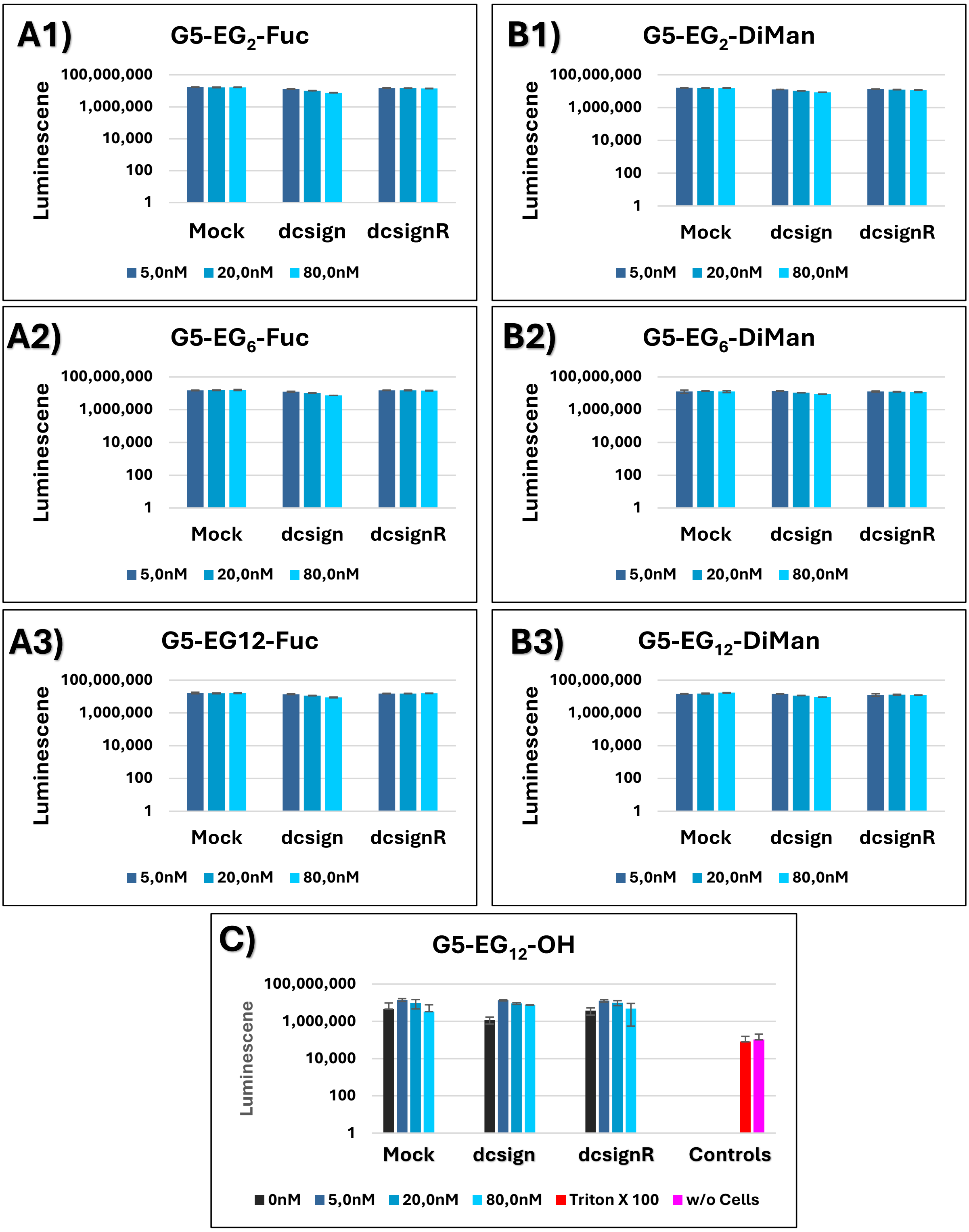

Figure S40. Evaluation of G5-EG$_n$-Fuc/DiMan cytotoxicity toward 293T cells measured by Cell Titer-Glo Assay.[11] Cells treated with the detergent Triton X100 served a positive control for cytotoxicity which gave the same value as without cells. No dose-dependent reduction of cell viability (indicated by luminescence) was observed for G5-EG$_n$-Fuc (**A1-A3**) or G5-EG$_n$-DiMan (**B1-B3**) at the highest concentration used in antiviral tests, suggesting no measurable cytotoxicity. In fact, their cell viabilities are comparable to those treated with the G5-EG$_{12}$-OH control (**C**, G5 coated with LA-EG$_{12}$-OH ligand known to be noncytotoxic, see Ning et al., *JACS Au*, 2024, 4, 3295). The results of a single experiment performed with technical quadruplicates are shown. Error bars indicate standard deviations.

10. Supporting references

1. J. Ramos-Soriano, J. J. Reina, B. M. Illescas, N. de la Cruz, L. Rodríguez-Pérez, F. Lasala, J. Rojo, R. Delgado and N. Martín, *Journal of the American Chemical Society*, 2019, **141**, 15403-15412.
2. D. Budhadev, E. Poole, I. Nehlmeier, Y. Y. Liu, J. Hooper, E. Kalverda, U. S. Akshath, N. Hondow, W. B. Turnbull, S. Pohlmann, Y. Guo and D. J. Zhou, *Journal of the American Chemical Society*, 2020, **142**, 18022-18034.
3. Y. Guo, I. Nehlmeier, E. Poole, C. Sakonsinsiri, N. Hondow, A. Brown, Q. Li, S. Li, J. Whitworth, Z. J. Li, A. C. Yu, R. Brydson, W. B. Turnbull, S. Pohlmann and D. J. Zhou, *Journal of the American Chemical Society*, 2017, **139**, 11833-11844.
4. X. Ning, D. Budhadev, S. Pollastri, I. Nehlmeier, A. Kempf, I. Manfield, W. B. Turnbull, S. Pöhlmann, A. Bernardi, X. Li, Y. Guo and D. Zhou, *JACS Au*, 2024, **4**, 3295-3309.
5. R. Basaran, D. Budhadev, A. Kempf, I. Nehlmeier, N. Hondow, S. Pöhlmann, Y. Guo and D. Zhou, *Nanoscale*, 2024, DOI: 10.1039/D4NR00484A.
6. X. Ji, X. Song, J. Li, Y. Bai, W. Yang and X. Peng, *Journal of the American Chemical Society*, 2007, **129**, 13939-13948.
7. A. K. Saha and C. F. Brewer, *Carbohydrate Research*, 1994, **254**, 157-167.
8. H. D. Hill, J. E. Millstone, M. J. Banholzer and C. A. Mirkin, *ACS Nano*, 2009, **3**, 418-424.
9. Y. Guo, W. Bruce Turnbull and D. Zhou, in *Methods in Enzymology*, ed. B. Imperiali, Academic Press, 2018, vol. 598, pp. 71-100.
